# A systematic assessment of chemical, genetic, and epigenetic factors influencing the activity of anticancer drug KP1019 (FFC14A)

**DOI:** 10.18632/oncotarget.21416

**Published:** 2017-09-30

**Authors:** Upendarrao Golla, Swati Swagatika, Sakshi Chauhan, Raghuvir Singh Tomar

**Affiliations:** ^1^ Laboratory of Chromatin Biology, Department of Biological Sciences, Indian Institute of Science Education and Research (IISER), Bhopal 462066, India

**Keywords:** anticancer, KP1019, histones, transcriptomics, metal homeostasis

## Abstract

KP1019 ([trans-RuCl_4_(1H-indazole)_2_]; FFC14A) is one of the promising ruthenium-based anticancer drugs undergoing clinical trials. Despite the pre-clinical and clinical success of KP1019, the mode of action and various factors capable of modulating its effects are largely unknown. Here, we used transcriptomics and genetic screening approaches in budding yeast model and deciphered various genetic targets and plethora of cellular pathways including cellular signaling, metal homeostasis, vacuolar transport, and lipid homeostasis that are primarily targeted by KP1019. We also demonstrated that KP1019 modulates the effects of TOR (target of rapamycin) signaling pathway and induces accumulation of neutral lipids (lipid droplets) in both yeast and HeLa cells. Interestingly, KP1019-mediated effects were found augmented with metal ions (Al^3+^/Ca^2+^/Cd^2+^/Cu^2+^/Mn^2+^/Na^+^/Zn^2+^), and neutralized by Fe^2+^, antioxidants, osmotic stabilizer, and ethanolamine. Additionally, our comprehensive screening of yeast histone H3/H4 mutant library revealed several histone residues that could significantly modulate the KP1019-induced toxicity. Altogether, our findings in both the yeast and HeLa cells provide molecular insights into mechanisms of action of KP1019 and various factors (chemical/genetic/epigenetic) that can alter the therapeutic efficiency of this clinically important anticancer drug.

## INTRODUCTION

Cancer is an extremely diverse disease characterized by uncontrolled proliferation of abnormal cells that expands into surrounding tissues. The major factors for oncogenic transformation and cancer development include not only genetic changes in the proto-oncogenes, tumor suppressor genes, and DNA repair genes [1, 2], but also the aberrant pattern in epigenetic mechanisms such as DNA methylation and histone modifications [3, 4]. In addition, errors in cell division, DNA damage, substantial exposure to environmental toxic chemicals, and radiation can cause cancer. Although there are more than 200 anticancer drugs listed by the National Cancer Institute in the USA (http://www.cancer.gov/cancertopics/alphalist), combating cancer is troublesome due to their distinctive genetic and phenotypic heterogeneity, failure in early detection, inefficient chemotherapy treatment and its associated severe adverse effects, and development of drug-resistance. The failure to decipher and target the multiple underlying pathways of molecular carcinogenesis is a major challenge for anticancer drug development. Hence, recent efforts are being made towards developing additional therapeutic molecules targeting the plethora of cancer mechanisms to achieve higher therapeutic efficiency (specific to cancer cells) with lesser or no adverse effects [5].

The clinical success of platinum-based drugs (cisplatin, oxaliplatin, carboplatin) as potential candidates for the treatment of several cancers has highlighted the importance of metal-based therapeutics in medicine. Over the past decade, ruthenium-based complexes have emerged as leading candidates for the next-generation anticancer therapeutics, and many are in the pipeline. The Keppler group has developed many ruthenium (III) based co-ordination complexes, of which KP1019 (indazolium [*trans*-RuCl_4_(1H-indazole)_2_]) has demonstrated most promising anticancer activity with minimal side-effects in the undergoing clinical trials [6]. During pre-clinical development, KP1019 has shown activity against a wide variety of cancer cell lines, advanced and metastatic solid malignancies, without dose-limiting toxicity [7]. Strikingly, Heffeter et al. reported that KP1019 activity is not hampered by conventional mechanisms of intrinsic drug resistance and it has very less probability of acquiring insensitivity during therapy. Accordingly, KP1019 was found effective against cancer cell lines that were resistant to other anticancer drugs such as doxorubicin, thus emerging as a promising anticancer drug of choice for the treatment of drug-resistant tumors [8].

In addition to its anticancer property, KP1019 was found effective against β-amyloid aggregate induced neurotoxicity *in vitro*, thus might be used for the treatment of Alzheimer's disease, a neurodegenerative disorder [9]. Considering the therapeutic potential of KP1019, its sodium salt KP1339 (sodium [*trans*-RuCl_4_(1Hindazole)_2_]) was developed to improve its solubility. It has shown promising results in phase-I trials against a variety of solid tumors [10], non-small cell lung cancer, sarcoma and colorectal cancers [11]. Several other efforts have been made to improve KP1019 stability, solubility and cytotoxic activity by changing the functional groups to Osmium [12], developing the CF3 derivatives [13], using lipiodol-based emulsions [14], polymer-based micelles [15], and biodegradable poly-(lactic acid) based nanoparticles [16].

The multiple oxidation states, hydrolysis behavior, and slow ligand exchange rates of ruthenium makes KP1019 a promising candidate for anticancer drug development. Ru (III) complexes including KP1019 coordinate with serum proteins (albumin, and transferrin) through their accessible histidine residues [17] and are believed to be then specifically transported to the solid tumor and retained there for longer hours [18]. Later, it was dissected that the cellular uptake of KP1019 occurs by both transferrin-dependent and transferrin-independent mechanisms [19]. Additionally, KP1019 is widely believed to be a ‘prodrug’ that gets activated by ‘reduction’ to highly reactive Ru (II) selectively in the hypoxic environment of cancer cells [6]. However, recent findings in contrast to the redox activation hypothesis, showed that ruthenium was present in its +III oxidation state (Ru^3+^) after treatment with ruthenium complexes (KP1019 and KP1339) in yeast cells [20] and tissues (tumor/liver/kidney) of a mouse bearing tumors of SW480 colon cancer cells [21]. However, the redox potential of ruthenium was found to be reduced in the presence of biologically relevant reductants (ascorbic acid, glutathione) and thereby modulating the activity of KP1019 under physiological conditions [1, 22, 23]. Since the proposed mechanisms including ‘transferrin-mediated uptake’ and ‘redox activation’ for selective accumulation of KP1019 in tumors remain highly debatable, there is a need for further clarifications on the oxidation state and speciation pattern of these ruthenium compounds in cellular environments.

In addition to its strong affinity for plasma proteins, cell fractionation analysis upon KP1019 treatment evidenced strong distribution of ruthenium to nuclei, which is rich in DNA, histone proteins, and chromatin regulators [19, 20]. Subsequently, KP1019 has been shown to bind with histone H3 [24], DNA-modeling nucleotides [22], DNA [25], and is capable of creating inter-strand crosslinks, resolution of which can produce double strand breaks [25]. Further evidence suggests that KP1019 increases the rate of recombination and mutations in yeast, activates DNA damage response wherein DNA repair occurs via mismatch repair, base excision repair, translesion synthesis (TLS), and recombination [26]. In addition, a recent study indicates that KP1019 treatment causes DNA-dependent cell cycle arrest in pre-anaphase, and leads to accumulation of large-budded yeast cells with abnormal nuclear position (spans bud neck) [27]. In our early attempts, we have shown that KP1019 causes osmotic stress, leading to the activation of Hog1 (p38) MAP kinase in yeast [28]. We have also demonstrated that KP1019 can interact specifically with histone H3, and evict histones from the nucleosome *in vitro*, and is found to be more effective in the absence of chromatin/histone modifying enzymes [24].

Despite the success of KP1019 and current wealth of information largely available on its chemical characterization *in vitro*, the genetic targets, various cellular pathways and epigenetic events selectively targeted to mediate its anticancer activity *in vivo* are mostly unknown. The present study is thus aimed to decipher the molecular mechanisms, genetic targets and critical residues of conserved histone H3/H4 that are required to mediate KP1019-induced cytotoxicity through genome-wide transcriptomics, chemical-genetics approach, and functional screening of synthetic yeast histone H3/H4 mutant library, respectively. Interestingly, comprehensive analysis of our results indicated that KP1019 alters metal ion homeostasis, lipid homeostasis, and can modulate the target of rapamycin (TOR) pathway in addition to its already characterized effects on cell cycle and DNA damage. Furthermore, we report for the first time that cytotoxic potential of KP1019 can be modulated (enhanced/repressed) in the presence of various metal ions, reductants, ethanolamine (ETA) and also by substitution mutations on the histone H3/H4 residues *in vivo*. Here, we majorly employed budding yeast, a robust model organism which has been implicated in biomedical research over the years to decipher the conserved genetic targets and mode of action of bioactive molecules including anticancer drugs, pollutants and toxicants [29, 30]. Interestingly, we could mimic some of the findings obtained with yeast in human cervical cancer cells (HeLa) that correlate well with the selective cytotoxicity of KP1019, and perhaps also its sodium salt, KP1339 in tumor cells.

## RESULTS

### Functional enrichment analysis of KP1019 transcriptome

**Figure 1:**
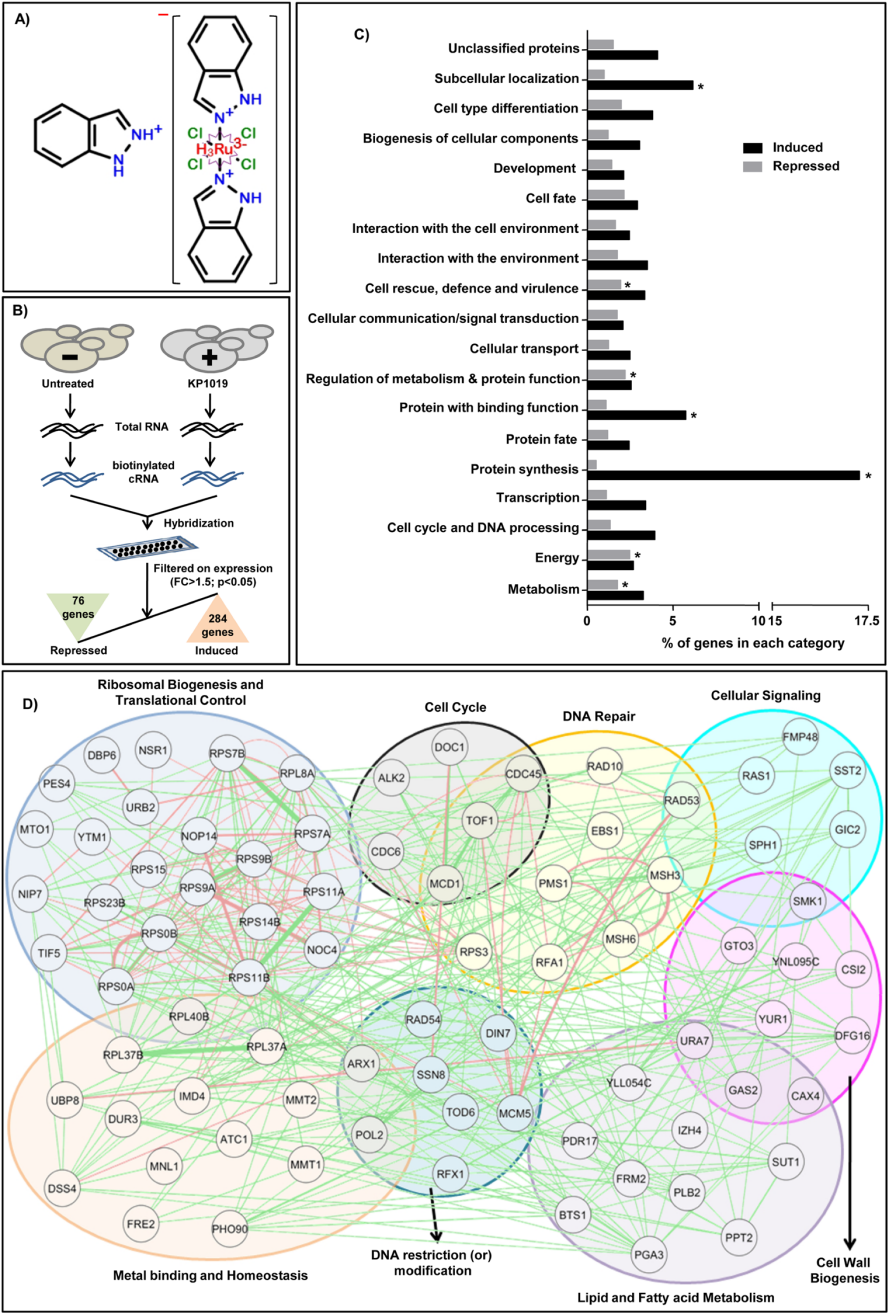
Global transcriptomics analysis upon KP1019 treatment **(A)** Chemical structure of KP1019 (Indazolium trans-[tetrachlorobis(1H-indazole)ruthenate (III)]). **(B)** Schematic representation of the procedure followed for the microarray analysis of yeast cells after KP1019 treatment. Total RNAs were extracted from wild-type cells (W1588-4C) that were left untreated (DMSO control) or treated with KP1019 (50μg/ml; 3h), and then hybridized to yeast genome GeneChip arrays according to standard Affymetrix protocol. KP1019 treatment leads to significant upregulation of 284 genes and downregulation of 76 genes (P<0.05; fold change>1.5). **(C)** Functional classification of KP1019 transcriptome according to MIPS (The Munich Information Center for Protein Sequences) showed significant (^*^P<0.05) enrichment of genes coding for cellular rescue/defense, metabolism, energy, and protein synthesis, etc. **(D)** Regulatory network analysis by *GENEMANIA* tool showed the reported genetic (green) and protein interactions (pink) exist among different genes induced by KP1019. The genes were clustered based on their functional role in various cellular processes including cell cycle, DNA repair, cell signaling, ribosomal biogenesis, lipid and metal homeostasis.

KP1019 (Figure 1A) is a lead ruthenium-based anticancer molecule undergoing phase-II clinical trials [31]. In line with earlier reports, KP1019 exhibited dose-dependent cytotoxicity as indicated by the decreased growth of wild-type yeast cells (Supplementary Figure 1). To understand the effects of KP1019 on different cellular processes, we performed global transcriptome analysis of wild-type yeast cells treated with a sublethal dose (50μg/ml) of KP1019 for 3h. Interestingly, KP1019 treated yeast cells showed significant upregulation of 284 genes and downregulation of 76 genes (p≤0.05; fold change≥1.5) (Figure 1B, Supplementary Table 2). To investigate the cellular processes targeted by KP1019, the differentially expressed genes (DEG’s) were classified according to MIPS (Munich Information Center for Protein Sequences) functional categories [32]. We found that the processes related to energy, metabolism, protein synthesis, proteins with binding function, subcellular localization, cell rescue and defense were enriched significantly in KP1019 transcriptome (Figure 1C). Consequently, our functional enrichment analysis showed that genes belonging to cell cycle checkpoint, DNA damage repair, ribosomal biogenesis and translational control were significantly enriched in induced transcriptome (Supplementary Table 3), whereas the genes involved in cell morphogenesis, cytokinesis, transcription, and protein binding were enriched in repressed transcriptome of KP1019 (Supplementary Table 4). The transcriptional induction of DNA damage repair genes (*RNR1/RNR3/HUG1/RAD10/RAD53/RAD54*) and repression of cytokinesis, cell morphogenesis genes (*DSE3/DSE4/EGT2*) by KP1019 were in accordance with earlier studies [24, 27], and hence validated our transcriptome data (Supplementary Table 3, Supplementary Table 4). Further to understand the regulatory associations between different DEGs, we used *GENEMANIA* tool and constructed functional interaction network of KP1019 induced genes that were clustered based on their role in various biological processes such as cell cycle, cellular signaling, cell wall biogenesis, DNA modification, DNA repair, metal homeostasis, lipid and fatty acid metabolism, ribosomal biogenesis and translational regulation (Figure 1D). Altogether, our functional enrichment analysis of KP1019 transcriptome revealed several cellular processes that were targeted to exhibit its cytotoxicity.

### Intact cell wall integrity (CWI) pathway is essential for KP1019 tolerance

**Figure 2:**
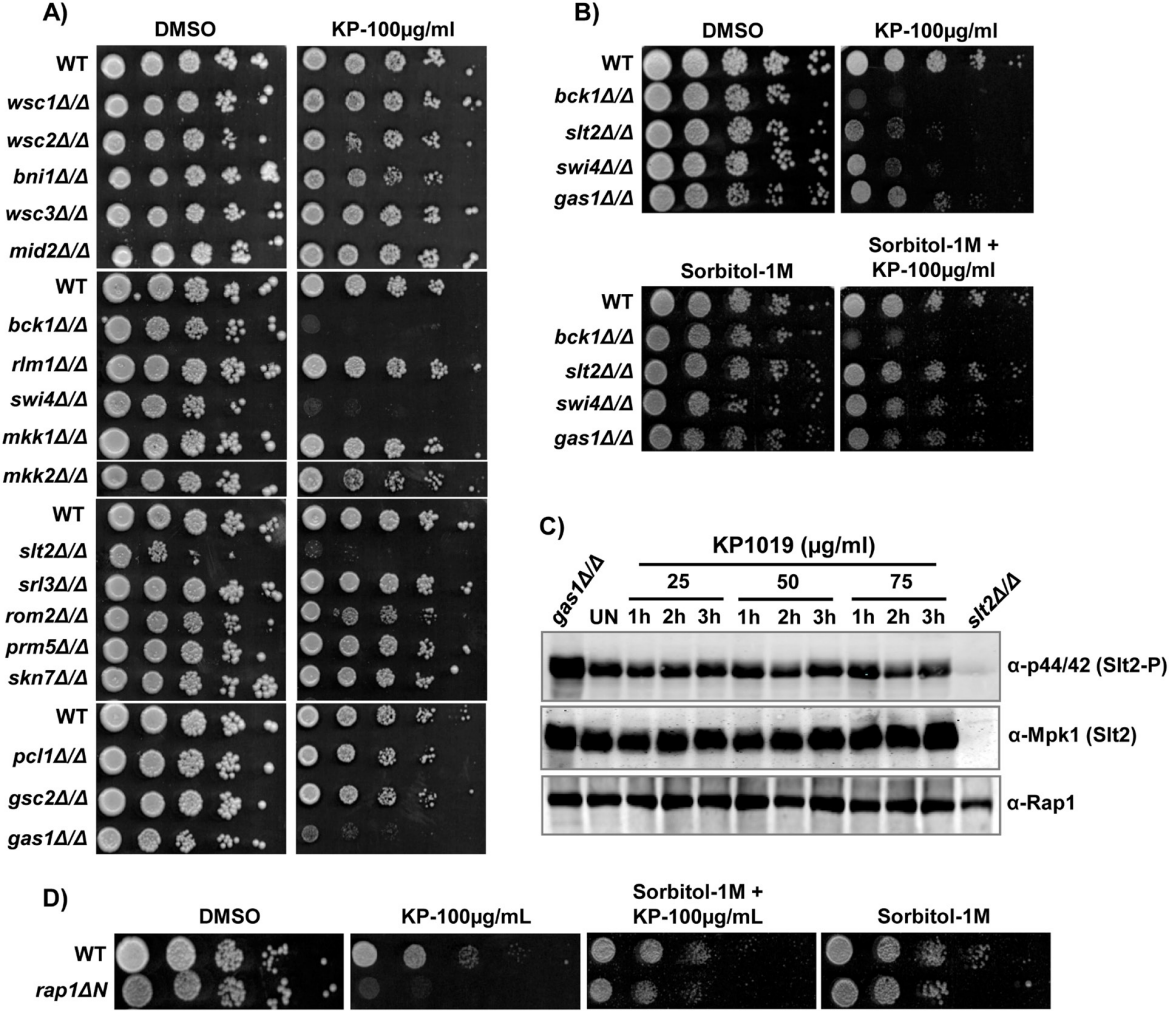
KP1019 mediates its cytotoxicity through cell wall integrity (CWI) pathway **(A** and **B)** CWI pathway null mutants exhibited sensitivity to KP1019 (A) and supplementation of sorbitol as an osmotic stabilizer rescues the KP1019 sensitivity (B). Ten-fold serial dilutions of WT (BY4743) and indicated CWI pathway mutants were spotted onto SC-agar plates supplemented without (DMSO) or with KP1019 (100μg/ml) and sorbitol (1M) in alone or the combination. The plates were imaged after 48h. **(C)** KP1019 treated cells do not activate Slt2 (Mpk1) MAP kinase. The exponentially growing wild-type (BY4743) yeast cells were left untreated or treated with different doses of KP1019 (25, 50, and 75μg/ml) for indicated time points and the whole-cell protein extracts were subjected to immunoblotting analysis using indicated antibodies. Anti-Mpk1 and Anti-Rap1 signals were served as the control for checking Slt2 levels and protein loading respectively. The gas1Δ cells that exhibit higher levels of phosphorylated Slt2 due to defective cell wall were used as a positive control. **(D)** Loss of Rap1 N-terminal region leads to increased KP1019 sensitivity. Ten-fold serial dilutions of WT (*RAP1*) and *rap1ΔN* mutant cells were spotted onto SC-agar plates supplemented without (DMSO) or with KP1019 (100μg/ml) and sorbitol (1M) in alone or the combination. The plates were imaged after 48h.

Earlier, we have shown that KP1019 activates stress responsive Hog1 (p38) MAP kinase of High Osmolarity Glycerol (HOG) pathway [28]. Since HOG and CWI pathways cooperate with each other for relieving various stress conditions [33] and transcriptional induction of cell wall biogenesis genes by KP1019 (Figure 1D), we were encouraged to assess the role of CWI signaling pathway in its tolerance. Interestingly, our growth assay results showed that the null mutants of MAPKK kinase (*BCK1*), MAP kinase (*SLT2*), a transcriptional activator (*SWI4*), and glucanosyltransferase (*GAS1*) were found sensitive to KP1019 (Figure 2A, Supplementary Figure 2A), thereby indicating the prime role of intact CWI pathway in its tolerance. To reason the sensitivity of CWI mutants to KP1019, we supplemented growth medium with sorbitol, an osmotic stabilizer. Notably, osmotic stabilization of growth media with sorbitol significantly relieved the KP1019 effects (Figure 2B). Thus, it indicates that the sensitivity of CWI pathway null mutants might be credited to osmotic perturbation by KP1019. Further to test whether KP1019 activates CWI pathway, we performed western blotting analysis using an anti-p44/42 antibody that detects the dually phosphorylated Slt2 (Thr190/Tyr192), a central MAP kinase of CWI pathway [34]. Surprisingly, we failed to observe significant activation of Slt2 MAP kinase upon KP1019 treatment (Figure 2C). The levels of both phosphorylated (α-p44/42) and unphosphorylated (α-Mpk1) forms of Slt2 kinase were found similar in both untreated and KP1019 treated cells (Figure 2C). Hence, the differential activity of KP1019 towards the activation of these stress-responsive MAPKs Hog1 and Slt2 might be credited to their distinct functional role in cellular apoptosis and growth [35]. Furthermore to confirm that indeed KP1019 targets CWI pathway, we screened mutants of Rap1, a Repressor/Activator site binding Protein, for KP1019 tolerance (Supplementary Figure 2B). Intriguingly, *rap1ΔN* cells that have defective cell wall [36] were found to be KP1019 sensitive, which was relieved upon sorbitol supplementation (Figure 2D). Collectively, our results indicate that KP1019 may affect directly or indirectly the cell wall and intact CWI pathway is required for its tolerance.

### KP1019 treatment alters Ca^2+^ ion homeostasis, increases lipid droplet accumulation, and Ethanolamine (ETA) supplementation remediates its cytotoxicity

**Figure 3:**
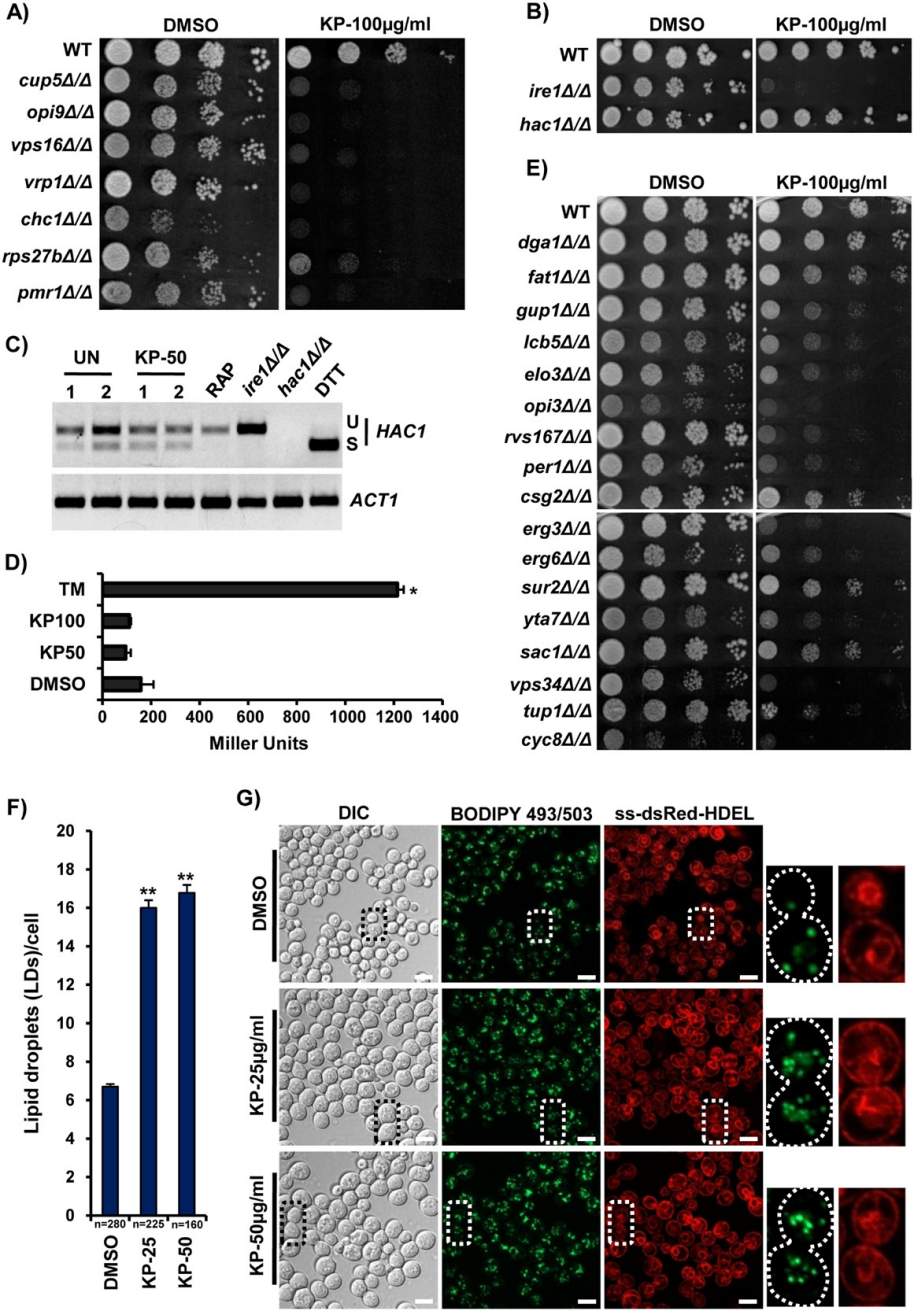
KP1019 treatment causes lipid droplets (LDs) accumulation **(A** and **B)** The gene products involved in Ca^2+^ ion homeostasis (A) and Ire1 kinase involved in unfolded protein response (UPR) are required for KP1019 tolerance (B). Ten-fold serial dilutions of WT and indicated mutants were spotted in absence or presence of KP1019 and the growth was monitored after 48h. **(C)** KP1019 does not induce maturation of *HAC1* mRNA. Total RNAs isolated from the wild-type (W1588-4C) cells that were left untreated or treated with KP (50μg/ml; 3h), rapamycin-RAP (200nM; 3h), and dithiothreitol-DTT (2mM; 1h) were reverse transcribed to cDNA. The maturation of *HAC1* was assessed by detecting levels of both the unspliced (U) and spliced (S) forms of *HAC1* mRNA using intron-specific splicing primers. Cells treated with DTT, an ER stress inducer were served as a positive control, whereas *ire1Δ* cells were served as a negative control. *hac1Δ* cells were used to check the specificity of primers used. *ACTIN* levels were used as control. **(D)** KP1019 does not alter UPRE activity. The wild-type (BY4741) yeast cells harboring *UPRE::lacZ* reporter plasmid were grown to exponential phase and then left untreated (DMSO solvent control) or treated with KP1019 (50, 100μg/ml) for 3h and Tunicamycin-TM (1μg/ml; 1h). β-galactosidase activity was measured using ONPG as a substrate and represented the results in terms of Miller units. The results are Mean±SEM, from two independent experiments each performed in triplicate. ^*^P<0.05 considered significant compared to DMSO control (Student's t-test). **(E)** KP1019 alters lipid homeostasis, and the null mutants of genes involved in lipid biosynthesis and metabolism are sensitive to KP1019. Ten-fold serial dilutions of WT and indicated mutants were spotted in absence or presence of KP1019 (100μg/ml) and the growth was monitored after 48h. **(F)** Quantification of KP1019-induced LDs formation in yeast cells. The wild-type (BY4743) yeast cells were grown to exponential phase and left untreated (DMSO control) or treated with KP1019 (25, 50μg/ml) for 3h, then stained with BODIPY 493/503 dye to visualize the LDs under ApoTome microscope. The LDs stained with BODIPY 493/503 in ApoTome images were counted and represented as the number of LDs per cell (Mean ± SEM; n=280 for DMSO, 225 for KP25, and 160 for KP50). ^**^P<0.001 (compared to DMSO control) were considered significant (Student's t-test). **(G)** KP1019 treatment induces accumulation of LDs in yeast cells without altering ER architecture. Wild-type yeast cells harboring ss-DsRed-HDEL reporter (stains both cortical and nuclear ER) were grown to exponential phase and left untreated (DMSO control) or treated with KP1019 (25, 50μg/ml) for 6h, then stained with BODIPY 493/503 dye to visualize the LDs. Cells treated with KP1019 had shown increased cell size and the number of LDs (compared to control) with retaining the intact cortical and nuclear (‘bowtie’ shaped) ER architecture. The representative images from three independent experiments were shown. Scale bar represents 5μm.

Recently, KP1019 has been shown to inhibit ATP-dependent translocation of calcium (Ca^2+^) ion *in vitro* by Sarco-endoplasmic reticulum Ca^2+^-ATPase (SERCA) pumps, which plays a critical role in intracellular calcium ion homeostasis [37]. To gain further insights into the mode of action of KP1019, we performed growth assay with null mutants of genes involved in Ca^2+^ ion homeostasis to identify genetic targets that are essential for KP1019 tolerance (Supplementary Figure 3A). Interestingly, our results showed that the null mutants of *PMR1* (Golgi membrane P-type ATPase ion pump), *CUP5* (Vacuolar-ATPase proton pump), *CHC1* (Clathrin heavy chain), *VRP1/OPI9* (Verprolin), *RPS27B* (Ribosomal protein of the small subunit), and *VPS16* (member of Vacuolar protein sorting complex) were found sensitive to KP1019 (Figure 3A). The null mutants of *CHC1/CUP5/OPI9/PMR1/RPS27B/VPS16/VRP1* were reported to have increased accumulation of calcium ions [38], and their sensitivity to KP1019 was validated further by growth curve analysis (Supplementary Figure 3B). So, we propose that KP1019 treatment alters intracellular calcium ion homeostasis. The importance of Ca^2+^ ion levels in endoplasmic reticulum (ER) functioning [39] and ER-mediated effects of KP1339, a sodium salt of KP1019 [40], encouraged us to ascertain whether KP1019 has any effect on ER or not. We performed growth assay with the null mutants of *IRE1* and *HAC1*, the prime regulators and mediators known for activation of unfolded protein response (UPR)/ER-stress response genes in yeast [41], and found only *ire1Δ* cells to be hypersensitive to KP1019 (Figure 3B). This further instilled in us to check the activation of UPR upon KP1019 treatment by checking the splicing of *HAC1* mRNA and expression of *UPRE-lacZ* (a reporter of UPR signaling) as indicated earlier [39]. Surprisingly, KP1019 treated cells failed to show increased levels of matured (spliced form) *HAC1* mRNA (Figure 3C) and β-galactosidase activity (Figure 3D). Hence, our results indicated that the sensitivity of *ire1Δ* cells to KP1019 might be due to their defective cell wall [42], depleted levels of membrane lipid component inositol [43], or credited to the indispensable role of Ire1 in lipid homeostasis [44] rather than activation of UPR in ER. In corroboration to this, KP1019 induced transcriptome showed the enrichment of genes involved in lipid and fatty acids (FAs) homeostasis (Figure 1D). To determine the possible effects of KP1019 on lipid homeostasis, we then performed growth assay with 147 null mutants of genes involved in lipids/FAs metabolism for KP1019 tolerance (Supplementary Figure 4). Interestingly, our results showed that the loss of genes involved in endocytosis (*RVS167*), GPI-anchor biosynthetic process (*PER1*), lipid/FAs transport (*FAT1, GUP1*), lipid/FAs metabolism (*CSG2, ELO3, LCB5, OPI3, SUR2, VPS34*), phosphate metabolism (*SAC1, YTA7*), sterol biosynthetic process (*ERG3, ERG6*), and transcriptional repression (*CYC8, TUP1*) results in moderate to severe sensitivity to KP1019 (Figure 3E, Supplementary Figure 5A), confirming that KP1019 mediates its toxicity through alteration of lipid/FAs metabolism and thus lipid homeostasis. Further to test the effect of KP1019 on lipid metabolism, we looked at the profile of intracellular neutral lipid stores (lipid droplets) using a cell-permeable lipophilic fluorescent dye BODIPY 493/503 that specifically stains the storage neutral lipids, lipid droplets (LDs) [45]. Notably, our results showed that KP1019 treatment leads to accumulation of intracellular lipid stores as evidenced by a drastic increase in the number of LDs per cell (Figure 3F, Supplementary Figure 5B) and hence indicates that KP1019 alters cellular lipid homeostasis. Although we showed that KP1019 does not induce ER stress (Figure 3D), the accumulation of LDs and sensitivity of lipid homeostasis genes in ER to KP1019 demonstrates that it might interfere with the structure and functions of ER. As lipid homeostasis is primarily controlled by the ER, we tested the effect of KP1019 on ER architecture using cells harboring ss-dsRed-HDEL reporter that stains both the cortical and nuclear ER [46]. To our surprise, the cells exhibited accumulation of LDs with retaining the intact architecture of both the cortical and nuclear (‘bowtie’ shaped) ER even after 6h treatment with KP1019 (Figure 3G). Hence, we propose that KP1019 alters lipid homeostasis without affecting the architecture of ER.

**Figure 4:**
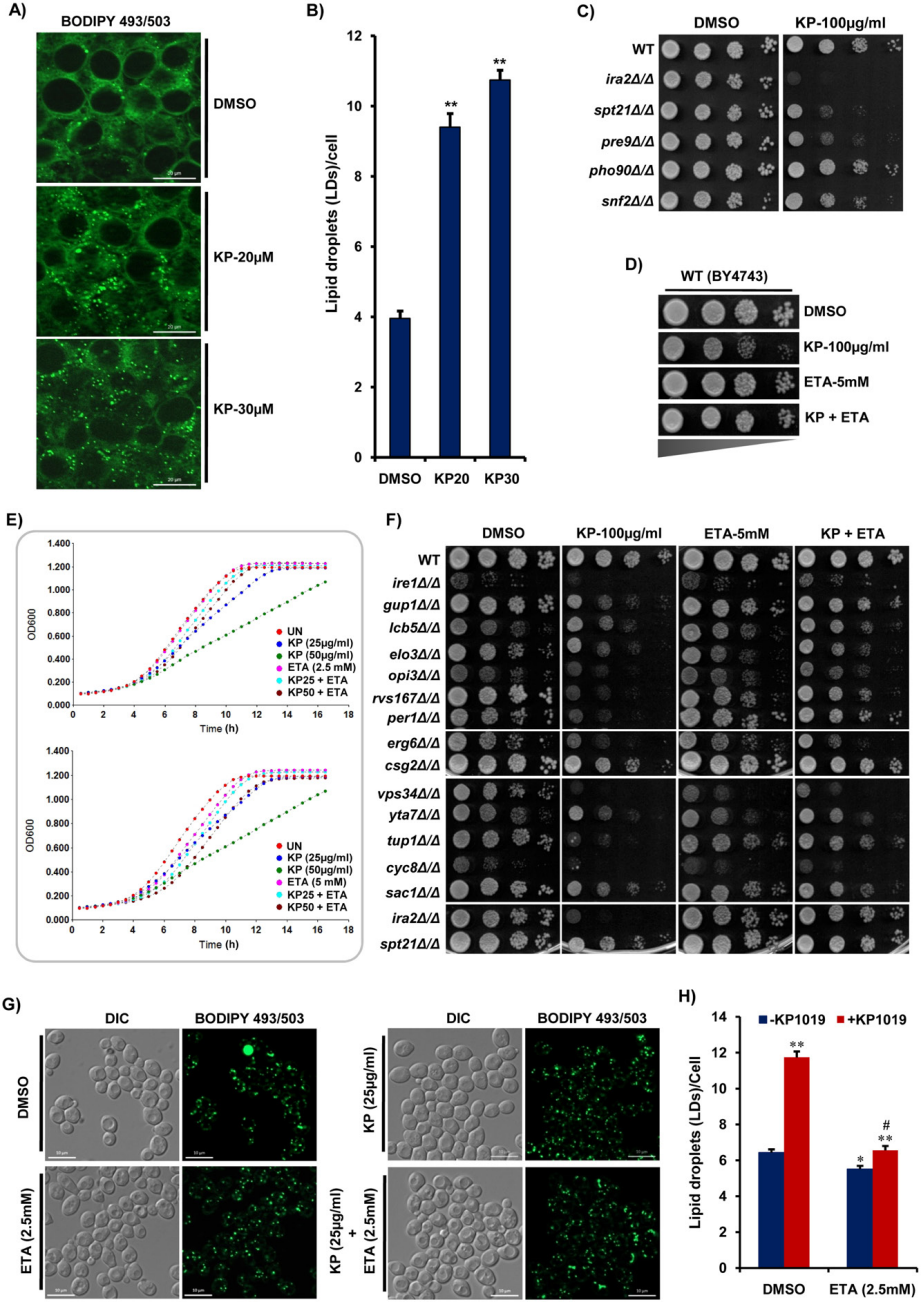
Supplementation of ethanolamine (ETA) suppresses KP1019-induced LDs accumulation and toxicity **(A** and **B)** Human cervical cancer (HeLa) cells exhibit increased LDs upon KP1019 treatment. HeLa cells treated with either DMSO (solvent control) or KP1019 (20, 30μM) were stained with BODIPY 493/503 dye and LDs were visualized using ApoTome microscope. The representative images from two independent experiments were shown and scale bar represents 20μm (A). The LDs stained with BODIPY 493/503 dye in (A) were counted and represented as number of LDs per cell (n=414; Mean ± SEM). ^**^P<0.001 (compared to DMSO control) were considered significant (Student's t-test) (B). **(C)** KP1019 is more effective in the mutants with higher lipid content. Ten-fold serial dilutions of WT and indicated mutants (with increased lipid content) were spotted in absence or presence of KP1019 (100μg/ml) and the growth was monitored after 48h. **(D** and **F)** Ethanolamine (ETA) supplementation neutralizes the KP1019-induced cytotoxicity. Ten-fold serial dilutions of wild-type (WT) BY4743 cells (D) and indicated KP1019 sensitive null mutants of lipid homeostasis (F) were spotted in the absence (DMSO control) or presence of KP1019 (100μg/ml) and ETA (5mM) in alone and combination. The growth was monitored after 48h. **(E)** KP1019-induced toxicity is suppressed in the presence of ETA. The exponentially growing wild-type (BY4743) cells were treated with either DMSO solvent (control) or indicated doses of KP1019 (KP) and ETA in alone or combination. The growth was monitored in terms of absorbance (OD600) for indicated period using a plate reader. **(G** and **H)** ETA supplementation remediates KP1019-induced LDs accumulation. The exponentially growing wild-type (BY4743) yeast cells were treated with either DMSO (solvent control) or KP1019 (25μg/ml) and ETA (2.5mM) in alone and combination for 3h. The cells were stained with BODIPY 493/503 dye and LDs were visualized using ApoTome microscope. The representative images from two independent experiments were shown and scale bar represents 10μm (G). The LDs stained with BODIPY 493/503 dye in (G) were counted and represented as the number of LDs per cell (n=150; Mean ± SEM). ^*^P<0.01, ^**^P<0.001 (compared to DMSO control) and ^#^P<0.001 (compared to only KP1019 treated) were considered significant (Student's t-test) (H).

Considering the conservation of lipid homeostasis pathways of yeast with mammalian models [47], we attempted to test the effect of KP1019 on intracellular lipid stores in HeLa cell lines. Amazingly, our results showed that KP1019 treatment induces the accumulation of LDs in HeLa cells too, similar to that of its effect in yeast cells (Figure 4A, 4B). These findings reasoned us to ask whether the LDs and enzymes involved in their formation play any role in KP1019 tolerance. So, we tested the effect of KP1019 on the quadruple null mutant (*dga1Δlro1Δare1Δare2Δ*) which can't synthesize the neutral lipids and therefore lacks LDs [49]. Our results showed that the effect of KP1019 on LDs deficient quadruple null mutant is same as that of wild-type cells (Supplementary Figure 5C). This motivated us to check the effect of KP1019 on the mutant cells (*ira2Δ, pre9Δ, pho90Δ, snf2Δ, spt21Δ*) that are known to have higher lipid content [50]. We found that *ira2Δ* cells are hypersensitive, whereas *pre9Δ* and *spt21Δ* exhibited mild sensitivity to KP1019 (Figure 4C), indicating that KP1019 effects are enhanced in the mutants with altered lipid homeostasis. Consequently, the null mutants of *IRA2* and *SPT21* showed a significant increase in LDs accumulation compared to wild-type untreated cells and the formation of LDs was further enhanced in these mutants (*ira2Δ, pre9Δ*, and *spt21Δ*) after KP1019 treatment similar to that of wild-type cells (Supplementary Figure 6).

The identification of *OPI3*, which catalyzes phosphatidylcholine (PC) biosynthesis via the Kennedy pathway, and *VPS34*, which synthesizes phosphatidylinositol (PI)-3-phosphate, here as genetic targets required for KP1019 tolerance (Figure 3E) encouraged us to check the effect of ethanolamine (ETA) supplementation on its activity. ETA is a precursor utilized by the Kennedy pathway for *de novo* synthesis of phosphatidylethanolamine (PE) and thus rescues most of the detrimental effects associated with low levels of phospholipids PE and PC [51]. Amazingly, ETA supplementation neutralized the toxic effects of KP1019 on wild-type yeast cells (Figure 4D, 4E) and the null mutants of lipid homeostasis (Figure 4F). In addition to the remediating effects on growth, ETA supplementation significantly decreased the accumulation of LDs induced by KP1019 treatment in yeast cells (Figure 4G, 4H), thus inferring the modulation of phospholipids synthesis and utilization by KP1019. Taken together, our results obtained both in yeast and HeLa cells strongly indicate that KP1019 treatment alters lipid homeostasis (biosynthesis, metabolism, and transport), and dietary ETA might interfere with its effectiveness.

### KP1019 antagonizes the effects of Rapamycin, an inhibitor of TOR (Target of Rapamycin) pathway

**Figure 5:**
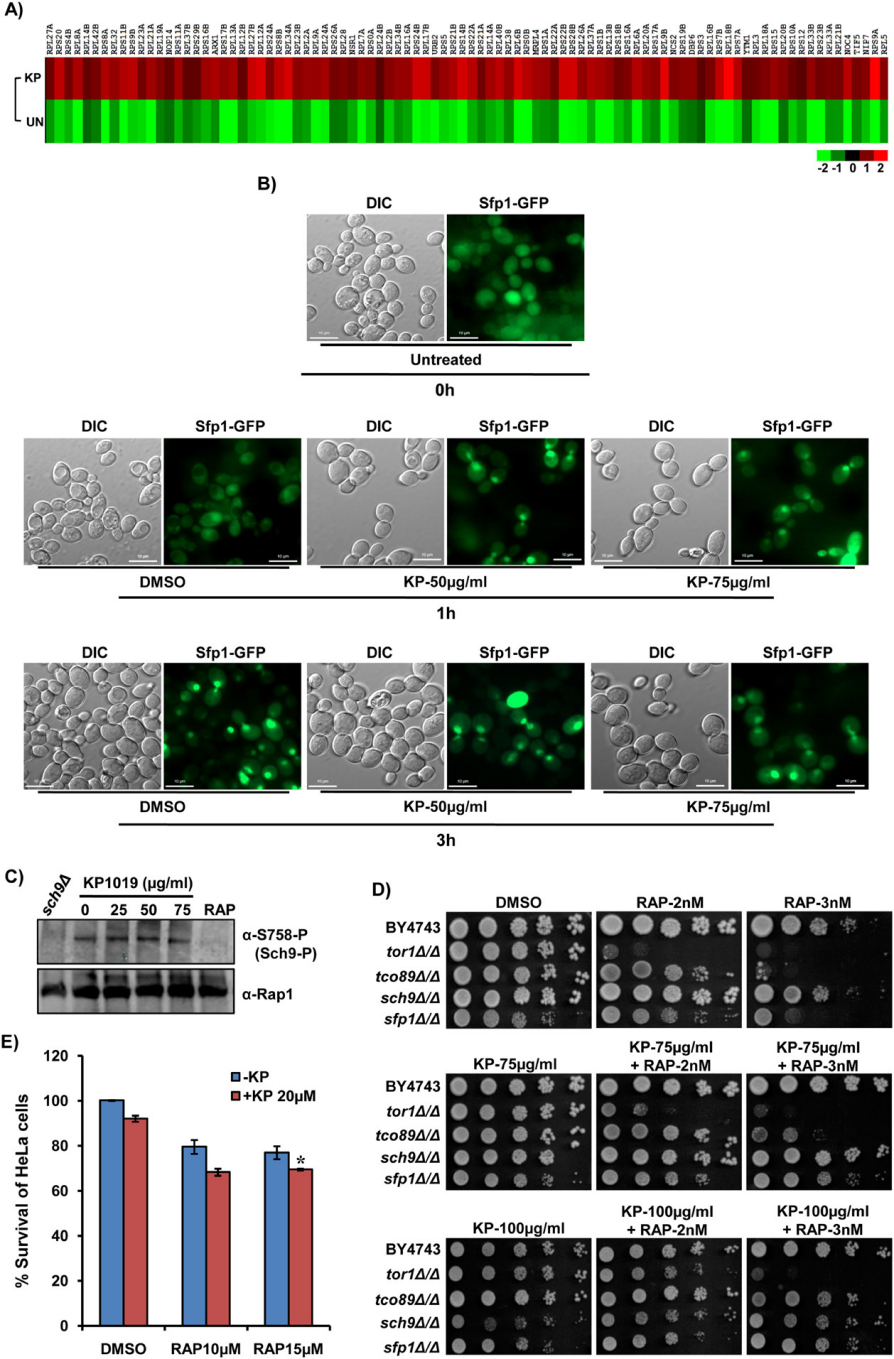
KP1019 treatment induces ribosomal biogenesis genes and modulates the effect of Rapamycin, an inhibitor of TOR pathway **(A)** Gene expression profile (heat map) of ribosomal biogenesis and translational control genes upon KP1019 (50μg/ml) treatment. The average normalized expression values (log_2_) for each gene from two independent experiments were illustrated by red-green color scale. **(B)** KP1019 treatment induces the early nuclear localization of Sfp1, a critical regulator of ribosomal biogenesis genes. Wild-type (BY4743) cells harboring *GFP*-tagged Sfp1 were grown to early exponential phase (OD600: 0.8-1) and left untreated (DMSO) or treated with KP1019 (50, 75μg/ml). The localization of Sfp1 was monitored after 1h and 3h of KP1019 treatment using ApoTome microscope. The KP1019 treated cells known to have nuclei spanning bud neck (‘bowtie’ phenotype) and the bowtie-shaped Sfp1-*GFP* signals upon KP1019 treatment indicates its nuclear localization. The representative images from three independent experiments were shown. Scale bar represents 10μm. **(C)** TOR pathway is functional/active in cells treated with KP1019 as indicated by Sch9 phosphorylation. The exponentially growing wild-type (BY4743) yeast cells were left untreated or treated with KP1019 (25, 50, and 75μg/ml) and rapamycin (200nM; RAP) for 3h. The whole-cell protein extracts were subjected to immunoblotting analysis of Sch9 phosphorylation using Anti-S758-P antibody. Anti-Rap1 signals were served as the control for checking protein loading. The *sch9Δ* and RAP-treated cells were used as negative controls. **(D)** KP1019 antagonizes the effects of rapamycin (RAP), an inhibitor of the TOR pathway. Ten-fold serial dilutions of wild-type (BY4743) and indicated TOR pathway null mutants were spotted onto SC-agar plates supplemented with DMSO or KP1019 and RAP at an indicated doses in alone or the combination. The plates were imaged after 72h. **(E)** The cytotoxicity of KP1019 in HeLa cells was slightly enhanced in combination with RAP, an immunosuppressant. The viability of HeLa cells treated with KP019 (20μM) and RAP (10, 15μM) in alone or combination was analyzed by MTT assay, and the results were represented as % survival (compared to DMSO control). The values are Mean±SEM, from two independent experiments each performed in duplicate. ^*^P<0.05 considered significant compared to DMSO control (Student's t-test).

The transcription of ribosomal protein (RP) genes is controlled by TOR pathway in accordance with the growth of cells, and rapidly downregulated in response to a variety of environmental stress stimuli that inhibits the cellular growth such as heat shock, nutrients starvation, and osmotic stress [52–54]. Despite the fact that KP1019 inhibits the growth of yeast cells (Supplementary Figure 1) and induce osmotic stress [28], our genome-wide transcriptome analysis of wild-type yeast cells treated with a sublethal dose of KP1019 showed upregulation of several RP genes (Figure 5A), and accordingly exhibited significant functional enrichment of genes involved in ribosomal biogenesis and translational control (Figure 1D, Supplementary Table 3). As Sfp1 is localized to the nucleus under optimal growth conditions and majorly regulates the transcriptional induction of RP genes, first we checked its localization upon KP1019 treatment using cells harboring GFP-tagged Sfp1 [55]. As expected, Sfp1-GFP is localized to the nucleus in exponentially growing wild-type cells that were left untreated for 3h (Figure 5B). In contrast, our microscopy results showed that Sfp1-GFP is localized to the nucleus within 1h treatment of early exponential phase cells with KP1019 compared to untreated (DMSO control) cells (Figure 5B). Hence, our findings indicate that the early nuclear localization of stress-responsive Sfp1 is in agreement with the transcriptional induction of RP genes upon KP1019 treatment. Since the nuclear localization of Sfp1 and transcriptional induction of RP genes is strongly correlated with the active TOR pathway signaling [56, 57], we then speculated that TOR pathway is functional in the cells stressed with KP1019. To check our hypothesis, we tested the levels of phosphorylated Sch9, a well-known substrate of yeast TOR complex 1 (TORC1), by immunoblotting analysis using Anti-S758-P antibody (gifted by Robbie J. Loewith) [58]. Interestingly, the levels of phosphorylated Sch9 were found to be similar in both untreated and KP1019 treated cells (Figure 5C), thus indicating that TORC1 is active and unaffected by the treatment with growth-inhibiting doses of KP1019.

Since TOR is active in KP1019 treated cells, we then performed genetic screening with the null mutants of TOR pathway to test its role in providing KP1019 tolerance and found none of them sensitive to KP1019 treatment (Supplementary Figure 7A). Moreover, the null mutants of TOR pathway showed activation of Rad53 kinase (Supplementary Figure 7B), increase in the cell size and formation of LDs (Supplementary Figure 7C, Supplementary Figure 7D) similar to that of wild-type cells upon KP1019 treatment. Therefore, our results illustrate that functional TOR signaling is not essential for exhibiting the toxic effects of KP1019. Further to understand the effects of KP1019 under TOR inactive condition, we performed growth assay with TOR pathway null mutants in the presence of both KP1019 and rapamycin (RAP), an allosteric inhibitor of Tor1 kinase and TOR pathway activation [59]. Intriguingly, we observed that the cytotoxic effects of RAP induced by TOR inactivation were suppressed by KP1019 in a dose-dependent fashion (Figure 5D), which is in accordance with our above results. This suggests that KP1019 interferes directly/indirectly with the processes affected due to inactivation of TOR by RAP. Moreover, the resultant effect upon co-treatment with RAP and KP1019 in liquid media is found to be dependent on their dose ratio since we observed both the additive (Supplementary Figure 7E) and slight antagonistic activity (Supplementary Figure 7F) with their different dose combinations. Given that RAP and other mTOR inhibitors are in clinical testing as anticancer agents, we were motivated to check the outcome of RAP and KP1019 co-treatment on the HeLa cells survival using MTT assay. Surprisingly, the combination of RAP and KP1019 showed a mild additive cytotoxic effect as indicated by the significant decrease in mean % survival of HeLa cells compared to only KP1019 treatment (Figure 5E). Altogether, these findings demonstrated that KP1019 do not require functional TOR signaling for mediating its cytotoxicity and its cytotoxicity potential can be modulated in the presence of mTOR inhibitors.

### KP1019 alters metal homeostasis, and the presence of various metal ions differentially modulates the KP109-induced cytotoxicity

**Figure 6:**
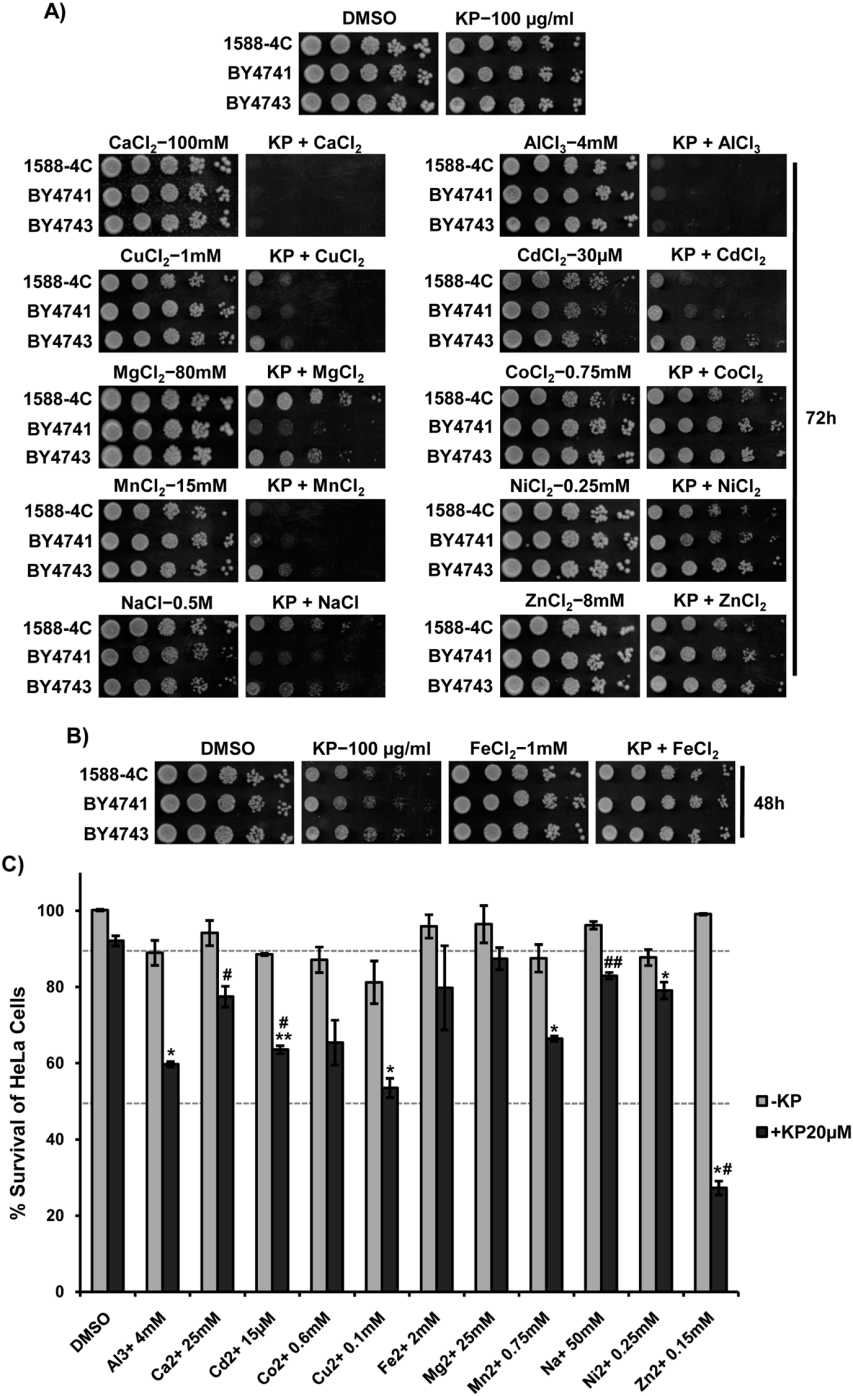
KP1019 alters metal homeostasis, and its cytotoxicity potential is modulated significantly in the presence of different metal cations **(A** and **B)** The effect of KP1019 was enhanced in the presence of various metal cations and suppressed by Fe^2+^ ions. Ten-fold serial dilutions of indicated wild-type yeast cells were spotted onto SC-agar plates supplemented with DMSO, indicated metal cation chlorides (A) or iron (Fe^2+^) chloride (B) in alone or combination with KP1019 (100μg/ml). The plates were imaged after indicated time. **(C)** KP1019 exhibit synergetic cytotoxicity with metal cations in human cervical cancer (HeLa) cells. The viability of HeLa cells treated with KP019 (20μM) and metal cation chlorides (Al^3+^, Ca^2+^, Cd^2+^, Co^2+^, Cu^2+^, Fe^2+^, Mg^2+^, Mn^2+^, Na^+^, Ni^2+^, and Zn^2+^) at indicated doses in alone or combination was analyzed by MTT assay, and the results were represented as % survival (compared to DMSO control). The values are Mean±SEM, from two independent experiments each performed in duplicate. ^*^P<0.05, ^**^P<0.01 (compared to only KP1019 treated) and ^#^P<0.05, ^##^P<0.01 (compared to only respective metal treated) were considered significant (two-tailed, paired Students t-test).

In continuation with the earlier report that showed an increase in Cu densities by KP1019 *in vitro* [60], our KP1019 transcriptome also envisaged upregulation of metal homeostasis genes (Figure 1D). Here to check the effect of KP1019 on metal ion homeostasis, we analyzed the growth of yeast cells upon co-treatment with KP1019 and various metal ions. For this, we first performed growth assay with wild-type cells in the presence of different metal cation chlorides to determine their effective dose (Supplementary Figure 8). Next, we monitored the effect of KP1019 and different metal cations (Al^3+^, Ca^2+^, Cd^2+^, Co^2+^, Cu^2+^, Fe^2+^, Mg^2+^, Mn^2+^, Na^+^, Ni^2+^, and Zn^2+^) alone or in combination on the growth of yeast cells by growth assays. Interestingly, the combination of KP1019 with Al^3+^, Ca^2+^, Cu^2+^, Mg^2+^, Mn^2+^, Na^+^, and Cd^2+^ was found to be lethal and exhibited enhanced cytotoxicity (Figure 6A). In contrast, the presence of Fe^2+^ ions neutralized the cytotoxic effects of KP109 on yeast cells (Figure 6B). Our growth curve analysis also confirmed that the toxicity potential of KP1019 is augmented in the presence of abovementioned metal ions except for Fe^2+^, which reduced the KP1019 toxicity (Supplementary Figure 9). These results reasoned us to ask whether ruthenium-based KP1019 interacts with other metal cations or not. For interaction studies, we monitored the characteristic UV-Visible absorption (360 and 420nm) spectra of KP1019 in the absence and presence of metal ions whose combinations adversely affected its toxicity. Notably, the combination of phosphate-buffered (Supplementary Figure 10A) or aqueous (Supplementary Figure 10B) solutions of metal ions (400μM) and KP1019 (65μM) showed changes in neither color nor the UV-Vis absorption spectra after 5h incubation at 37°C. However, the presence of Fe^2+^ (400μM) altered the characteristic absorption spectra of KP1019 in buffered condition (Supplementary Figure 10A) indicating the possible formation of its new species that is not/less toxic to yeast cells (Figure 6B).

Given that KP1019 alters Fe distribution without affecting its cellular content *in vitro* [60], the fate of Fe in yeast cells upon KP1019 treatment is unknown. To test whether KP1019 alters intracellular iron levels, we performed growth assay with null mutants of iron ion homeostasis genes encoding iron transporters and ferric reductases for its tolerance (Supplementary Figure 11A). Our results indicated that KP1019 does not interfere with intracellular iron ion levels as none of the tested mutants were sensitive to KP1019 (Supplementary Figure 11A). Moreover, the supplementation of Fe^2+^ relieves the effect of KP1019 on wild-type and null mutants of an iron-transport system to the same extent (Supplementary Figure 11B). So, it demonstrates that the diminished effects of KP1019 in the presence of iron ions could be accounted to its chemical inactivation (Supplementary Figure 10A) or functional antagonism with Fe rather than their competitive uptake through the iron-transport system (Supplementary Figure 11B). In contrast, our results with real-time analysis of growth in liquid media showed that the loss of Fe transport system (*FET3/FET5*) resulted in partial reduction of KP019 effectiveness compared to wild-type cells (Supplementary Figure 11C), thus revealing the role of iron-transport system in mediating the KP1019-induced toxicity probably through alteration of metal ion homeostasis [61].

Given that KP1019 induces oxidative stress and suppression of its toxicity by antioxidants in human cell lines [23], we asked whether supplementation of antioxidants can rescue KP1019 effects in yeast. In good agreement with the earlier reports, our results showed that the toxic effects of KP1019 were suppressed in the presence of biological antioxidant GSH (reduced glutathione) and its precursor N-acetylcysteine (NAC) *in vivo* (Supplementary Figure 11D, Supplementary Figure 11E). This suggests that KP1019 might be depleting the GSH levels and thus perturbing the redox homeostasis to mediate its toxicity (Supplementary Figure 11D, Supplementary Figure 11E). Although there is no activation of oxidative stress responsive genes including Yap1, the transcriptional induction of environmental stress response and cellular defense genes by KP1019 reasoned us to speculate that its enhanced toxicity with various metal ions could be due to further increase in the oxidative burden of yeast cells (Figure 1C).

For correlating above findings to KP1019 anticancer activity, we tested the effect of KP1019 treatment in combination with metal ions on HeLa cells viability by MTT assay. Interestingly, the cytotoxicity potential of KP1019 was significantly enhanced in the presence of metal ions such as Al^3+^, Cd^2+^, Cu^2+^, Mn^2+^, and Zn^2+^, while Ca^2+^, Co^2+^, Na^+^, Ni^2+^ slightly increased its cytotoxicity compared to only KP1019 treated cells (Figure 6C). Furthermore, the morphology of HeLa cells upon treatment with KP1019 and metal cations in combination showed the apoptotic features compared to their individual treatments (Supplementary Figure 12). Collectively, our findings indicate that KP1019 treatment alters metal ion homeostasis and its anticancer potential is significantly augmented in the presence of various metal cations.

### Histone H3 and H4 residues play a critical role in the regulation of KP1019-induced cytotoxicity

**Figure 7:**
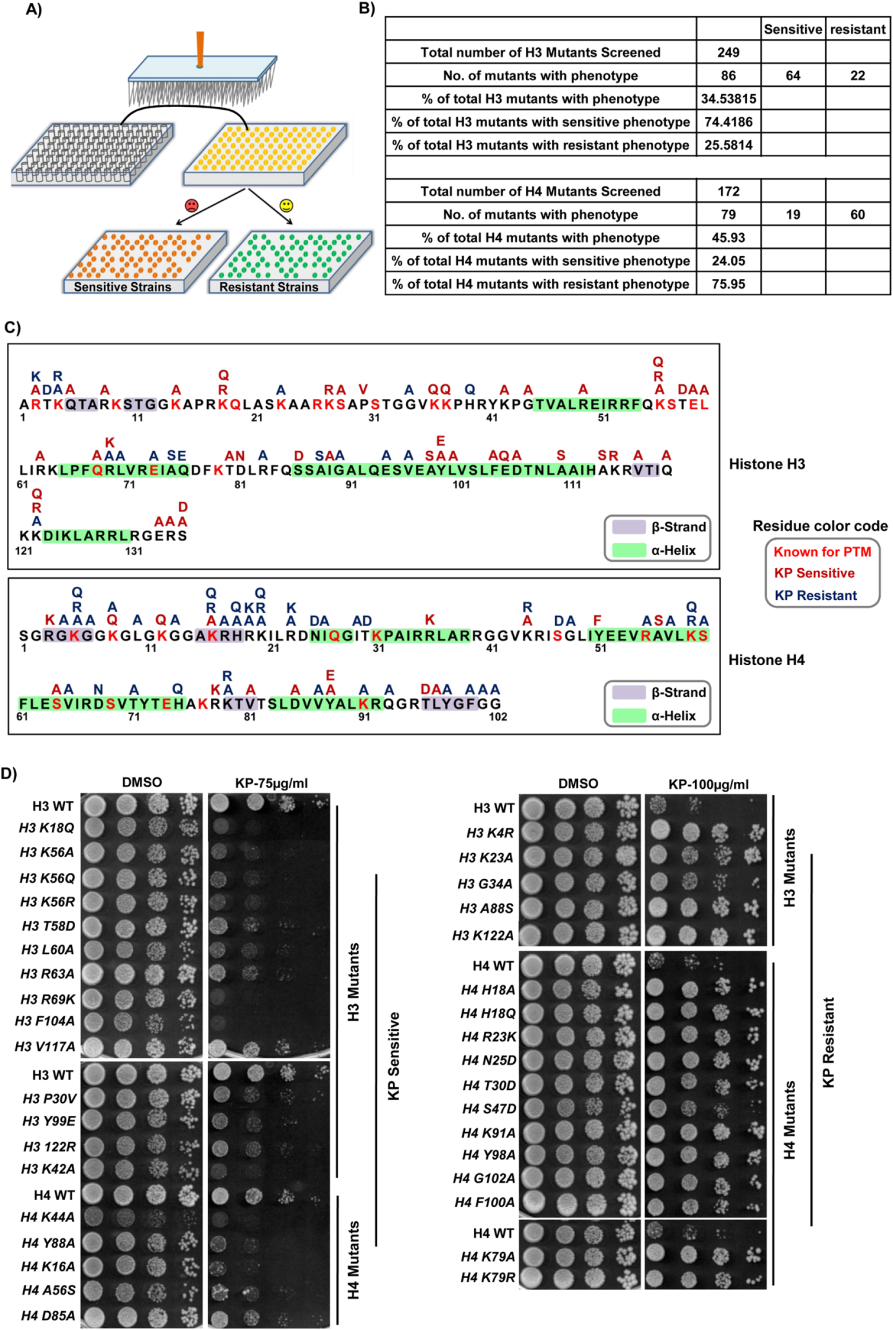
KP1019-induced toxicity is modulated by the mutations, loss or gain of PTMs on histone H3 and H4 residues **(A)** Schematic representation of high-throughput methodology adopted for the screening of yeast synthetic histone H3/H4 mutant library to identify the KP1019 sensitive and resistant mutants. The library mutants were grown in 96-well plates and spotted onto SC-agar plates containing different doses of KP1019. **(B)** Summarization of histone H3/H4 mutant library screening results indicates that KP1019 is highly potent in inducing cytotoxicity in histone H3 mutants whereas ineffective in histone H4 mutants. 34.5% of tested histone H3 mutants and 45.93% of tested histone H4 mutants exhibited phenotype (sensitivity/resistance) to KP1019. **(C)** Substitution mutations indicated above the histone H3, and H4 sequence (yeast) are sensitive (brown) or resistant (blue) to KP1019. The residues that are known for their PTMs (red) and contribution to the secondary structure are indicated within the sequence (based on UniProt database). **(D)** Validation of top-scored mutants those exhibited phenotype to KP1019. Ten-fold serial dilutions of wild-type (WT) and indicated KP1019 sensitive and resistant histone H3/H4 mutants were spotted onto SC-agar plates supplemented with DMSO or KP1019 (75, 100μg/ml). The plates were imaged after 48h.

Histones are highly conserved proteins that undergo a variety of post-translational modifications (PTMs), which are majorly concentrated on amino termini (tails) of histone H3 and H4 additional to some PTMs occur in their structural domains, required for chromatin plasticity and thus coordinating gene regulation mechanisms [62]. Despite the fact that we could not see significant changes in the levels of global histone modifications upon KP1019 treatment earlier [24], here we attempted to analyze the chromatin features of KP1019 transcriptome using ChromatinDB database. Interestingly, we found that the promoters of genes induced by KP1019 treatment have depleted occupancy of all four core histones with the enrichment of transcriptionally active acetylation marks (Supplementary Figure 13A), while its repressed transcriptome does not exhibit enrichment of any chromatin features (Supplementary Figure 13B). This suggests that KP1019 treatment affects the occupancy and PTMs of histones at specific gene promoters rather than entire genome for orchestrating transcriptional regulation. The ability of KP1019 to interact with histone H3 and cause eviction of histones from nucleosome *in vitro* [24], further fuelled our quest for unraveling the role of histone residues and their respective PTMs in translating KP1019-mediated cellular effects. To test this, we employed high-throughput screening of synthetic histone H3 and H4 mutants library derived by Dai et al. [63] for KP1019 tolerance (sensitive/resistant) (Figure 7A). The doses of KP1019 for screening the histone library were obtained by growth assay with wild-type cells. Accordingly, we used 50μg/ml and 100μg/ml of KP1019 for identifying the sensitive and resistant mutants respectively (Supplementary Figure 14A). Remarkably, our high-throughput screening of histone H3/H4 mutants library with KP1019 (Supplementary Figure 14B) led to the identification of 86 (≈34.5%) H3 and 79 (≈46%) H4 mutants that demonstrated altered phenotypes to KP1019 (Supplementary Table 5, Supplementary Table 6), and the results were summarized in Figure 6B. Although we found the total number of KP1019 sensitive (≈74.5% of all hits for H3 and ≈24% of all hits for H4) and resistant (≈25.5% of all hits for H3 and ≈76% of all hits for H4) mutants were almost equal (≈82), it is evident from our results that more robust KP1019 sensitive phenotypes were identified for H3 mutants and resistant phenotypes for H4 mutants relative to H4 and H3 respectively (Figure 7B). So, our data suggest that histone H3 and H4 may have entirely opposing roles in the regulation of KP1019-mediated effects, where H3 has considerably involved in potentiating while H4 in neutralizing its effects. The H3 and H4 residues whose substitutions exhibited phenotype (sensitive/resistant) to KP1019, and that are known for prone to PTMs were represented with a color code and highlighted in the primary sequence of H3 and H4 accordingly (Figure 7C). For quantifying the phenotypic response of mutants to KP1019, we allotted the scores numerically from 2 to 4 with positive or negative values as described in methods section subsequent to validation of KP1019 sensitive (Supplementary Figure 15) and resistant mutants (Supplementary Figure 16). For further confirmation, top-scored mutants that exhibited sensitivity and resistance against KP1019 were validated by growth assays (Figure 7D, Supplementary Figure 17A), and growth curve analysis (Supplementary Figure 17B, 17C). Provided that KP1019 destabilizes the nucleosome *in vitro* and causes DNA damage [24], we attempted to justify the enhanced and decreased effect of KP1019 in sensitive and resistant alleles with regard to aberrant global nucleosome positioning and DNA damage repair pathways, which were assessed by MNase (*Micrococcal Nuclease)* accessibility assay and Rad53-kinase activation respectively. To our surprise, both the KP1019 sensitive (H3-K18Q) and resistant (H3-K4R) mutants exhibited similar global nucleosome architecture (Supplementary Figure 18A) with proper activation of DNA repair checkpoint Rad53-kinase and induction of ribonucleotide reductases (Rnr1/Rnr2) (Supplementary Figure 18B) compared to wild-type cells upon KP1019 treatment. So, our results indicate the need for further studies to understand the detailed mechanisms and factors responsible for enhancing or decreasing the KP1019 induced cytotoxicity in the histone H3/H4 mutants.

### Analysis of structural and functional features of histone H3/H4 residues modulating the KP1019-induced cytotoxicity

**Figure 8:**
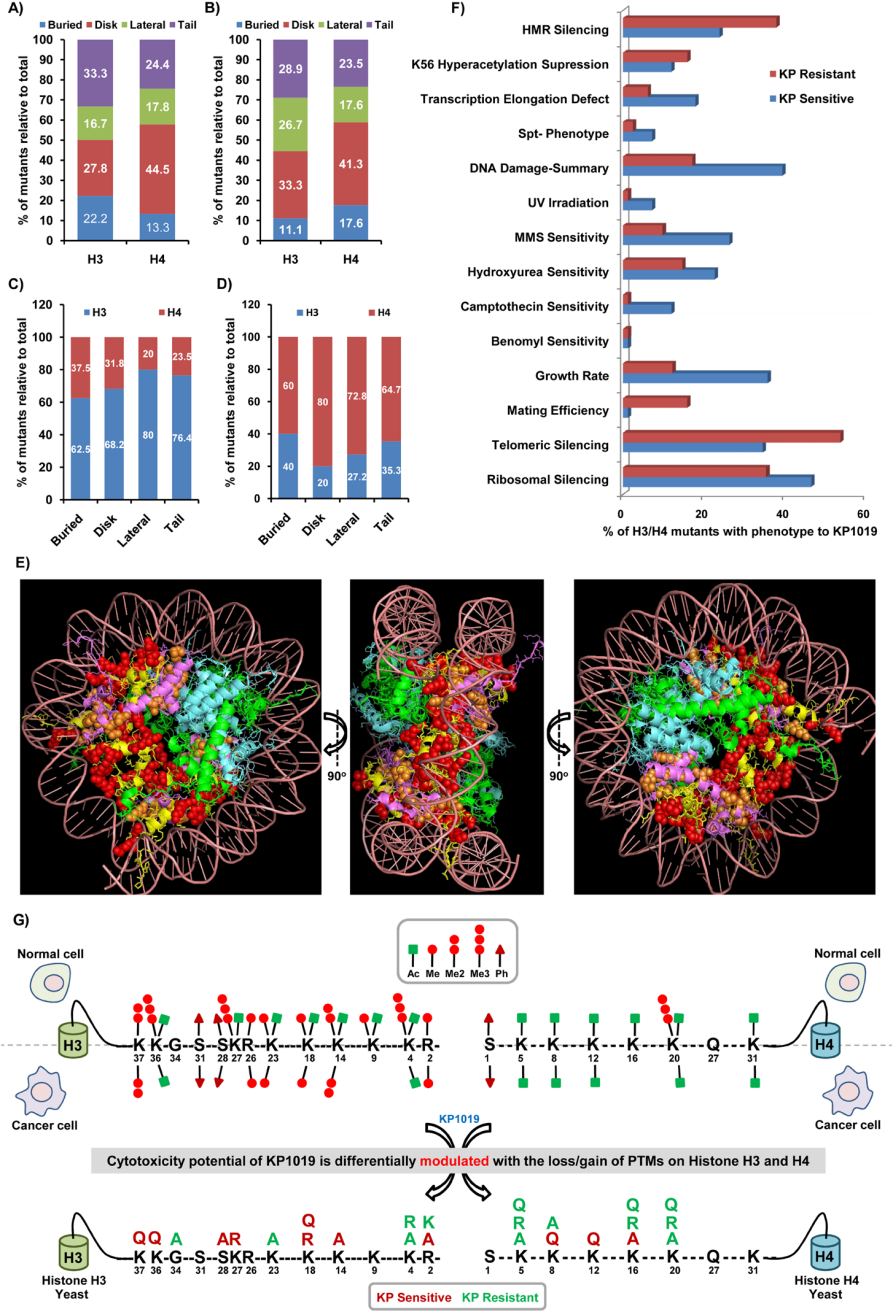
Functional analysis of histone H3/H4 residues that are exhibiting phenotype to KP1019 **(A** and **B)** The % mutants of histone H3 and H4 residues positioned in different structural domains of the nucleosome (buried, disk, lateral, and tail) relative to that of the respective total number of mutants exhibited sensitivity (A) or resistance (B) to KP1019. **(C** and **D)** The % distribution of histone H3 and H4 mutants based on their residues location in different structural domains of the nucleosome (buried, disk, lateral, and tail) relative to that of the total number of mutants (sum of H3 and H4) exhibited sensitivity (C) or resistance (D) to KP1019. **(E)** KP1019 sensitive histone H3 (red) and H4 (ochre) substitution residues are indicated in the 3D structure of yeast nucleosome (PDB ID: 1ID3). The nucleosome consists of all the four core histones H2A (green), H2B (cyan), H3 (yellow), and H4 (pink), and DNA (maroon) wrapped around the histone core. **(F)** The histone H3/H4 residues whose substitutions exhibited phenotype to KP1019 have associated with different functional phenotypes. The functional phenotype information for each of residues was extracted from HistoneHits database, and the number of histone H3/H4 mutants associated with each phenotype was represented as % relative to that of the total number of KP1019 sensitive and resistant mutants. **(G)** The toxicity potential of KP1019 is modulated differentially with the loss/gain of PTMs on histone H3/H4 residues. A hallmark of several cancers is the deregulation of PTMs on histone H3 and H4 tail residues in cancer cells compared to normal cells. The yeast substitution mutants of those homologous residues have exhibited differential sensitivity to KP1019 and thus suggest the possible influence of histone H3/H4 PTMs signatures on the anticancer potential of KP1019 in mammalian cancer cells.

To get functional insights of the histone H3/H4 mutants that displayed differential phenotype to KP1019, we extracted the structural and phenotypic information associated with each of KP1019 sensitive (Supplementary Table 5) and resistant (Supplementary Table 6) histone alleles as annotated in the HistoneHits database [64]. The position of amino acid residues of histones in the nucleosome roughly partitioned into four major geographical domains: buried (protein core that does not exposed to the solvent), disk surface (protein surface that does not contact DNA), lateral (protein surface that contacts DNA), and tail (unstructured in the crystal) [64]. Our detailed analysis of the geographical distribution of histone H3/H4 residues to nucleosome domains revealed that disk and tail residues are the major contributors for KP1019 sensitivity (Figure 8A) and resistance (Figure 8B). Additionally, the relative comparison of histone H3 with H4 mutants indicated that more of lateral and tail residues of histone H3 significantly contributed to KP1019 sensitivity (Figure 8C), while the disk and lateral residues of histone H4 majorly contributed to KP1019 resistance (Figure 8D). It is also evident that the tails of H3 and H4 have antagonistic roles as the loss or gain of PTMs on H3 tail resulted in sensitive phenotype, whereas H4 tail showed resistant phenotype to KP1019 (Figure 8C, 8D). Subsequently, the nucleosomal positioning of histone H3 and H4 residues whose substitution mutation considerably decreased the KP1019 tolerance is illustrated in 3D nucleosome structure (Figure 8E). It further revealed that most of the sensitive (H3/H4) residues present on the exposed surface (disk) and primarily involved in DNA wrapping/unwrapping (lateral), and hence critical for transcriptional regulation (Figure 8E).

Furthermore, we attempted to understand the functional roles of H3 and H4 residues that conferred phenotype to KP1019 by analyzing the phenotypic data for genetic screens of histone mutants accessible at HistoneHits database. Interestingly, our analysis of functional phenotypes associated with the KP1019 responding mutants emphasized that the mutation of residues involved in ribosomal silencing, telomeric silencing, growth rate, HMR locus silencing, transcription elongation defects, and increased sensitivity to DNA damaging agents (Camptothecin, HU, MMS, UV) conferred sensitivity to KP1019, whereas the mutation of residues involved in ribosomal silencing, telomeric silencing, mating efficiency, H3K56 hyperacetylation, and HMR locus silencing conferred resistance to KP1019 (Figure 8F). The mutants that exhibited phenotype to ribosomal silencing assay and KP1019 might provide correlations between anticancer activity of KP1019 and ribosomal synthesis, which affects cell growth and may increase susceptibility to cancer [65]. Considering the significance of histone point mutations, histone SNPs, along with covalent histone modifications in tumorogenesis and tumor development [66, 67], we attempted to find a relationship between the cancer epigenetic signatures/histone code with the altered KP1019 activity. Amazingly, we found a strong correlation between the human cancer specific histone modification signatures with the enhanced or suppressed effects of KP1019 in corresponding yeast mutants (Figure 8G). Although it is too far to relate our yeast histone mutant phenotypes of KP1019 with human cancer epigenomics and therapeutics, our findings strongly indicate that the cytotoxicity potential of KP1019 might be influenced by the histone substitution mutations and PTMs. Hence, future studies are needed to focus in this direction to elucidate the impact of altered epigenetic modifications on the anticancer potential of KP1019.

## DISCUSSION

In this study, we have elucidated the mode of action of KP1019, a ruthenium-based anticancer drug that has entered phase-II clinical trials after exhibiting successful stabilization of several cancers (colon, endometrium, liver, and tongue) in phase-I trials without any major adverse effects [6]. Our systematic findings in yeast and HeLa cells revealed that the cytotoxic potential of KP1019 could be influenced either by chemical (metals/reductants/ETA), genetic, or epigenetic factors. We have attempted to understand its off-target effects along with possible approaches to potentiate and neutralize the effects of KP1019. A global transcriptomics approaches such as microarray facilitates to identify the comprehensive set of genes differentially regulated in response to a condition of interest [68]. Transcriptional profiling of KP1019 treated cells exhibited differential regulation of only 5% of the genome (284 genes induced; 76 genes repressed) specifically. Functional enrichment analysis of KP1019 transcriptome unraveled its probable mode of action and indicated that KP1019 exhibits its effects by affecting several cellular processes such as cell cycle, cellular signaling, DNA damage repair, cell wall biogenesis, metal homeostasis, lipid homeostasis, and ribosomal biogenesis (Figure 1D). Consistent with our KP1019 transcriptome in yeast, recent studies with other ruthenium-based anticancer drugs (RDC11 and RuT_7_) in cancer cells also showed functional enrichment of genes belonging to various biological processes including cell cycle, cell morphology, cell signaling, DNA damage repair, protein synthesis, and ribosomal biogenesis [69, 70]. Hence, we propose that the KP1019 transcriptome profile and associated pathways obtained in yeast can be extrapolated to understand the possible conserved mechanisms through which it exerts anticancer activity in cancer cells.

In this study, we found that the activity of KP1019 was drastically decreased by ETA (Figure 4E), Fe^2+^ (Figure 6B), GSH (Supplementary Figure 11E), and moderately by Sorbitol (Figure 2B). To reason the suppression of KP1019 effects in the presence of various chemical agents (ETA, Fe^2+^, GSH, and Sorbitol), we measured the uptake of KP1019 by ICP-MS (Inductively Coupled Plasma Mass Spectrometry) and UV-Vis Spectrometry. Interestingly, our analysis of intracellular accumulation of KP1019 (in terms of Ru levels) by ICP-MS revealed that only GSH significantly reduced its uptake for suppressing the activity, while other agents (ETA, Fe^2+^, and Sorbitol) do not (Supplementary Figure 19A). The ICP-MS results were supported by UV-Vis estimation of residual KP1019, wherein alteration of its UV-Vis absorption spectrum in the presence of ETA and GSH infers the possible formation of new KP1019 species that is not/less toxic to yeast cells (Supplementary Figure 19B). Our findings indicate the need for further detailed studies to understand both the chemical and functional aspects of KP1019 inactivation by the abovementioned agents.

In budding yeast and mammals, an evolutionarily conserved Ser/Thr protein kinase Target of Rapamycin (TOR) signaling pathway involved in nutrient sensing, is a critical regulator of cell growth as it controls several processes including transcription, translation, and ribosomal biogenesis [71, 72]. Our results confirm that the TOR pathway is functional in KP1019 treated cells as envisaged by transcriptional induction of ribosomal biogenesis and translational control genes, early nuclear localization of stress- and nutrient-sensitive transcriptional factor Sfp1, phosphorylated Sch9, and functional antagonism of KP1019 with the RAP, an inhibitor of TOR pathway (Figure 5). Since TOR signaling activity is known to negatively regulate the Pkc1 kinase of CWI pathway that regulates functions related to cell growth and integrity under nutrient rich conditions [73], our failure to observe the activation of MAP kinase Mpk1 (p44/42) upon KP1019 treatment in yeast cells (Figure 2C) could be credited to the functional TOR pathway (Figure 5). It is worth noted that the anticancer activity of KP1339 (a sodium salt of KP1019) was significantly enhanced by co-administering it with Sorafenib, one of the multi-targeted tyrosine kinase inhibitors [74]. The deregulation of cell growth and cellular signaling including TOR pathway are not only implicated in tumor development, but also in drug resistance against chemotherapy [75]. Hence, the inhibitors of TOR pathway including RAP have underscored as anticancer drugs in addition to their immunosuppressant activity [76]. Despite the conservation of TOR pathway from yeast to mammals, the functional antagonism that was observed between RAP and KP1019 in yeast cells (Figure 5D) could not be mimicked in HeLa cell lines *in vitro* (Figure 5E). Hence, our results do not encourage further studies aimed at elucidating the therapeutic outcome of KP1019 in combination with RAP.

Besides acting as a TOR pathway inhibitor, RAP along with other macrocyclic lactones (Ivermectin, cyclosporine) were shown to potentially inhibit the function of SERCA pumps, thus alters Ca^2+^ ion homeostasis [77]. Interestingly, recent study has shown that KP1019 also inhibit SERCA and interfere with ATP-dependent Ca^2+^ translocation *in vitro* [37]. We demonstrate here *in vivo* that Pmr1, a Ca^2+^/Mn^2+^ P-type ATPase involved in the transport of Ca^2+^ and Mn^2+^ ions to Golgi, is essential for KP1019 tolerance (Figure 3A). Given that Pmr1-dependent transport of Mn^2+^ ions to Golgi is essential for RAP sensitivity [78], we attribute the functional antagonism observed between anticancer drugs RAP and KP1019 in yeast cells to their competitive antagonistic interaction with different P-type ATPase enzymes (Na^+^/K^+^, H^+^/K^+^, and Ca^2+^/H^+^) directly or indirectly (Figure 5D). Given this hypothesis, it will be noteworthy to propose the use of KP1019 in combination with SERCA/P-type ATPase inhibitors, emerging anticancer drugs for achieving the synergetic therapeutic effect against multidrug-resistant tumors [79, 80].

P-type ATPases selectively catalyze the active transport of essential ions like H^+^, K^+^, Mn^2+^, Na^+^, Ca^2+^, Co^2+^, Cu^2+^, and Zn^2+^ to maintain proper gradients across diverse cellular membranes [79]. These trace metal ions are essential for the functioning of various metalloenzymes and are toxic at higher concentration. The tight regulation of ions involved in multifunctional signaling is vital for cellular survival. The derivatives of metal ions are implied nowadays as therapeutic drugs such as antibacterial, antifungal, and anticancer agents [81]. Given that KP1019 interfere with SERCA/P-type ATPase mediated Ca^2+^ translocation *in vitro* [37], we speculated that KP1019 can alter intracellular metal ion homeostasis. Consequently, our results demonstrated that the cytotoxicity potential of KP1019 is augmented in combination with Al^3+^, Ca^2+^, Cd^2+^, Cu^2+^, Mn^2+^, Na^+^, and Zn^2+^ ions (Figure 6). Since many heavy metal ions (Cd^2+^, Cu^2+^, and Zn^2+^) have the ability to affect the activity of P-type ATPase (Mg^2+^/Na^+^, K^+^/Ca^2+^) similar to KP1019 [82], the obtained synergetic toxicity of KP1019 with various metal ions is possibly due to altered homeostasis and/or increased metal ion load in combination. Besides, the affinity of transferrin to transport other metal ions (Al^3+^, Ni^2+^, Mn^2+^, Cr^3+^, Bi^3+^, Ru^3+^, Ti^4+^, V^4+^) in addition to Fe^3+^ [83, 84], and altered ability of KP1019 to bind with apo-transferrin loaded with other metal ions are expected to affect KP1019 uptake and hence its activity. Strikingly, Farah et al. reported that the concentrations and metal load of major elements (Al/Ba/Ca/Cr/Cu/Fe/Mg/Na/Pb/Se/Sr/Zn) were significantly higher in tumor tissues compared to normal tissues of human lung, breast, and liver [85]. To add to the earlier proposed tumor specific transferrin-mediated uptake and redox activation of KP1019, we show that higher metal loads or altered ion homeostasis in tumor also selectively exaggerates KP1019 activity in cancerous cells compared to normal healthy cells.

One of the major hallmarks of a cancer cell is their ability to undergo metabolic reprogramming from respiration to aerobic glycolysis (known as the Warburg effect) [86], for fulfilling the demands of rapidly proliferating cells. It does so either by increasing the uptake of exogenous lipids (dietary) or overactivating the *de novo* lipogenesis to continuously provide cholesterol and fatty acids (FAs) required for cell membrane synthesis, cell cycle progression, cytokinesis, and energy supply [87, 88]. The physiology and metabolic fluxes of cancer cells are similar to that of fermenting yeast cells and most of the lipids metabolizing enzymes critical for cancer cell proliferation are conserved in *S. cerevisiae* [48]. Excessive FAs, lipids, and cholesterol are stored in the form of LDs in almost all eukaryotic cells and higher LDs and stored-cholesteryl ester content in tumors are ascribed as hallmarks of cancer aggressiveness [89]. The recent chemotherapeutics have thus been designed to attenuate the proliferation of cancer cells through manipulating FAs synthesis, metabolism, and diminishing the FAs availability [88]. We demonstrate here that genes involved in lipid homeostasis are critical for KP1019 tolerance (Figure 3E). Furthermore, we show that KP1019 treatment leads to accumulation of LDs in both yeast (Figure 3F, 3G) and mammalian HeLa cells (Figure 4A, 4B). Hence, we speculate that KP1019 inhibits the synthesis of phospholipids (PE; PC) by interfering with FAs utilization. This in turn promotes accumulation of LDs to inhibit the proliferation and induce apoptosis of cancer cells. This is further exemplified by the fact that the effects of KP1019 were remediated in the presence of ETA (Figure 4D, 4E), a precursor utilized by the Kennedy pathway for *de novo* synthesis of phospholipids [51]. From the physiological point of view, the accumulation of LDs occurs when cells are either maintained in medium containing excess FAs/lipoproteins or exposed to stress stimuli leading to cell death [90–92]. Hence, LDs accumulation is considered as a hallmark of apoptosis [93], and this phenomenon is observed in different metabolic diseases [94, 95], induced by various forms of stress stimuli including mitochondrial dysfunction and oxidative stress [93, 96], ER stress [97, 98], osmotic stress [99], inhibition of MYC in cancer cells [100], and activation of TOR pathway [101]. Though our results strongly suggest that KP1019 alters lipid metabolism and induces LDs accumulation, the molecular mechanisms behind its effects are yet to be answered.

Although LDs arise typically from ER and reside in the cytoplasm, emerging evidence suggests that LDs could sequester enzymes, transcription factors, and chromatin components to control their availability in the nucleus [102]. Conversely, several anticancer and epigenetic drugs such as cisplatin, 5-Azacytidine, YC-1, and Entinostat effectively interfered with lipid metabolism to stimulate LDs accumulation [103–106]. The question, then is whether LDs accumulation as a cause or consequence of KP1019 effects on epigenetic events. Although the major target of KP1019 in eukaryotic cells is found to be DNA, our earlier findings showed that KP1019 forms adduct with histone H3 and evicts histones from nucleosome *in vitro*. The essentiality of histones and chromatin modifiers for KP1019 tolerance specify that it mediate its effects by modulating epigenetic events [24]. To assess how mutations and/or loss/gain of PTMs on histones H3/H4 modulate KP1019-mediated signaling and impact its effectiveness, we utilized yeast synthetic histone H3/H4 mutant library for functional screening with KP1019. Remarkably, our results demonstrated that KP1019 effects were majorly mediated through histone H3-dependant functions and neutralized by the histone H4-mediated functions (Figure 7B). These contrasting effects of KP1019 on H3 and H4 might be credited to the opposing roles of H3 and H4 PTMs in regulating nucleosome structure [107]. Moreover, the loss/gain of PTMs and histone code on tails tightly regulate the higher-order chromatin architecture by affecting histone-DNA interaction and thus nucleosome stability [108]. Because the histones in yeast are highly conserved and many residues are modified at the same sites as those found in higher eukaryotes, we attempted to correlate the differential activity of KP1019 observed with histone H3/H4 mutants to epigenetic signatures in different cancers and cell types. For instance, almost all types of cancers exhibited global loss of acetylation (Ac) at Lys16 and trimethylation (Me3) at Lys20 of histone H4 [109], and substitution mutations in these residues neutralized KP1019 effects in yeast, thus accrediting the possible role of these PTMs to cancer selective cytotoxicity of KP1019 (Figure 8G). Besides, the point mutations in histones [110], along with loss/gain of PTMs on Lys4, Lys9, Ser10, Lys14, Lys18, Lys23, Lys27, Gly34, and Lys36 of histone H3 have been linked to tumorogenesis and cancer progression [111–113], and the mutations on these residues influenced KP1019 effects positively/negatively in yeast cells (Figure 8G). Previously, Sharma et al. have observed that cancer cells employ chromatin-mediated dynamic survival strategies by presuming a reversible drug-tolerant state to eradicate the anticancer drugs [114]. Therefore, our findings with the functional screening of histone H3/H4 library against KP1019 may provide information about the critical residues and histone code that guide the cytotoxicity of KP1019 selectively to cancer cells and only to certain cell types. Further studies are needed to decipher how these substitution mutations on histone H3/H4 affect the nucleosome architecture and bring genome-wide alterations in the transcriptome. This will provide clues about the critical epigenetic processes that are involved in manipulating the therapeutic potential of KP1019.

**Figure 9:**
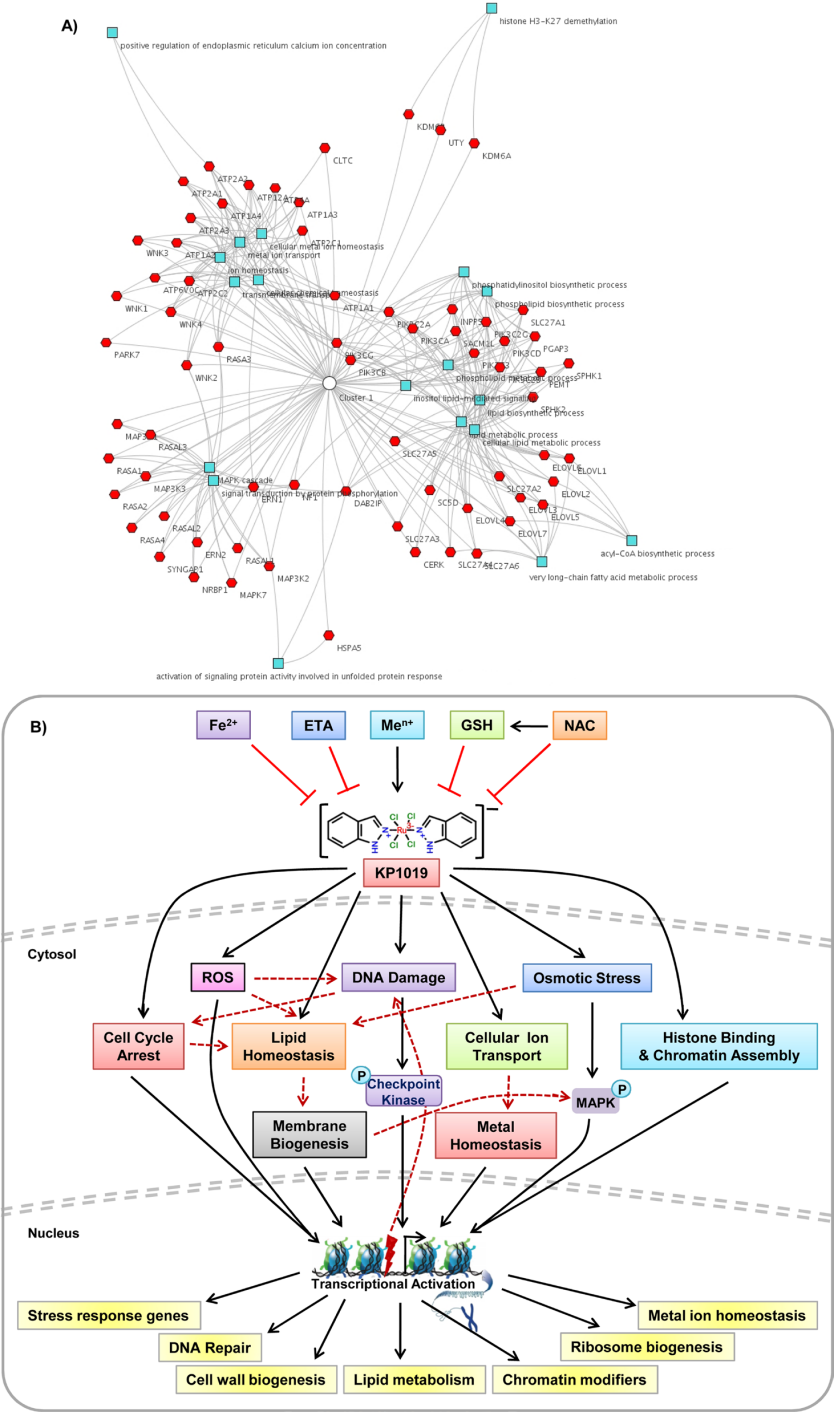
A proposed model depicting the mode of action of KP1019 **(A)** Functional classification of human homologous genes targeted by KP1019 in yeast using ToppCluster tool. **(B)** Model illustrating the mode of action of KP1019. Biologically active KP1019 induces ROS generation, causes DNA damage and thus cell cycle arrest, activates MAP kinase signaling, alters intracellular metal ion and lipid homeostasis, and also affects the chromatin assembly. The cells activate transcriptional response to alleviate KP1019 induced cellular damage. Moreover, the toxicity potential of KP1019 is enhanced in the presence of various metal ions (Me^n+^), whereas suppressed by the supplementation of Fe^2+^ ions, reduced glutathione (GSH), N-acetylcysteine (NAC), and ethanolamine (ETA).

The genetic targets of KP1019 identified here in yeast through chemical-genetics approach were shown in Table 1, and they are found to have multiple counterparts (homologs) in humans implicated in the progression of various cancers, neurological, immunological disorders and diseases (Table 1). We, however couldn't find any of the KP1019 targets to match with that of platinum-based anticancer drug Cisplatin except that of DNA repair [115]. We, thus propose that KP1019 mode of action is different from Cisplatin and can be used in combination for cancer treatment. The functional interactions existing among the human homologs of KP1019 target genes also indicated the similar biological processes that were found in yeast, including metal ion homeostasis, lipid/fatty acid biosynthesis and metabolism, MAPK signaling cascade, UPR, and histone modifiers (Figure 9A). Hence, our findings reveal the importance of unicellular budding yeast in assessing the mode of action of biologically active molecules including anticancer drugs. As budding yeast shares conserved basic cellular processes including metabolic and signaling mechanisms linked to cancer, we speculate that this study findings in yeast and mammalian HeLa cells will facilitate the understanding of diverse cellular targets of KP1019 and mechanisms of its anticancer activity in more complex higher eukaryotes such as humans [116].

**Table 1:** Identification of human homologs of yeast genes (that are required for KP1019 tolerance) and their functional role in the implication of various diseases using YeastMine tool

S.No.	Yeast KP target Gene	Human Homolog	OMIM Disease
1	BCK1	MAP3K1/2/3, NRBP1/2, WNK1/2/3/4	SRXY6, PHA2B, PHA2C, HSAN2A
2	CHC1	CLTC, CLTCL1	
3	CYC8	KDM6A/B, UTY	Kabuki Syndrome 2
4	ELO3	ELOVL1/2/3/4/5/6/7	ISQMR, SCA34, STGD3, SCA38
5	ERG6	COQ3, WBSCR27	
6	FAT1	SLC27A1/2/3/4/5/6	Ichthyosis Prematurity Syndrome
7	GUP1	HHAT, HHATL	
8	IRA2	DAB2IP, NF1, RASA1/2/3/4, RASAL1/2/3, SYNGAP1	NF1, JMML, WTSN, NFNS, BCC1, Parkes Weber Syndrome, MRD5
9	IRE1	ERN1/2	
10	LCB5	CERK, CERKL, SPHK1/2	Retinitis Pigmentosa 26
11	OPI3	PEMT	
12	PER1	PGAP3	HPMRS4
13	PMR1	ATP1A1/2/3/4, ATP2A1/2/3, ATP2C1/2, ATP4A, ATP12A	FHM2, AHC1, AHC2, Dystonia 12, CAPOS, Brody Myopathy, Darier-White Disease, AKV, BCPM
14	PRE9	PSMA4	
15	RPS27B	RPS27, RPS27L	
16	SAC1	INPP5F, SACM1L	
17	SLT2	MAPK7	
18	TUP1	ELP2	
19	VMA3	ATP6V0C	
20	VPS16	VPS16	
21	VPS34	PIK3C2A/B/G, PIK3C3, PIK3CA/B/D/G	Breast Cancer, Colorectal Cancer, CWS5, Gastric Cancer, Hepatocellular Carcinoma, IMD14, Keratosis, LCACC, MCAP, Nevus, Ovarian Cancer
22	VRP1	WIPF1/2/3	Wiskott-Aldrich Syndrome 2
23	YTA7	ATAD2, ATAD2B	

SRXY6, XY Sex Reversal 6; PHA2C, Pseudohypoaldosteronism, Type IIC; PHA2B, Pseudohypoaldosteronism, Type IIB; HSAN2A, Neuropathy, Hereditary Sensory and Autonomic, Type IIA; ISQMR, Ichthyosis, Spastic Quadriplegia, and Mental Retardation; SCA34, Spinocerebellar Ataxia 34; STGD3, Stargardt Disease 3; SCA38, Spinocerebellar Ataxia 38; NF1, Neurofibromatosis, Type I; JMML, Juvenile Myelomonocytic Leukemia; WTSN, Watson Syndrome; NFNS, Neurofibromatosis-Noonan Syndrome; BCC1, Basal Cell Carcinoma, Susceptibility to, 1; MRD5, Mental Retardation, Autosomal Dominant 5; HPMRS4, Hyperphosphatasia With Mental Retardation Syndrome 4; MIGRAINE, FHM2, Familial Hemiplegic, 2; AHC1/AHC2, Alternating Hemiplegia of Childhood 1/2; CAPOS, Cerebellar Ataxia, Areflexia, Pes Cavus, Optic Atrophy, and Sensorineural Hearing Loss; AKV, Acrokeratosis Verruciformis; BCPM, Benign Chronic Pemphigus; CWS5, Cowden Syndrome 5; IMD14, Immunodeficiency 14; LCACC, Lung Cancer Alveolar Cell Carcinoma; MCAP, Megalencephaly-Capillary Malformation-Polymicrogyria Syndrome.

In summary, we proposed a comprehensive model depicting the mode of action and cellular pathways targeted by KP1019 (Figure 9B). The toxic effects of KP1019 were positively influenced by various metal ions (Me^n+^), whereas negatively regulated by ETA, Fe^2+^, and reducing agents (GSH/NAC). KP1019 mediate its toxic effects through cell cycle arrest, DNA damage, oxidative stress, osmotic stress, by altering cellular signaling, metal homeostasis, transport, and lipid homeostasis. We also demonstrate that the efficiency of KP1019 and its effects are dependent on epigenetic signatures and histone code. Altogether, our detailed transcriptomics analysis and genetic targets identified in this study shed light on several cellular processes targeted by KP1019, and aid in understanding the molecular mode of action of this clinically important anticancer drug, and perhaps also its sodium salt (KP1339) that is undergoing clinical trials.

## MATERIALS AND METHODS

### Synthesis of KP1019

The anticancer ruthenium complex KP1019 was synthesized according to the established method [117]. RuCl_3_.3H_2_O was purchased from Sisco Research Laboratories Pvt. Ltd. (India), and indazole was obtained from Sigma-Aldrich (India). The solvents used in the synthesis were procured from Merck (India). The identity of KP1019 was confirmed using electrospray ionisation mass spectrometry (ESI-MS) coupled with liquid chromatography (LC-MS), and UV-Vis spectroscopy (Supplementary Figure 10). The ESI mass spectrum of KP1019 was found similar to that of our earlier observation [24] and other's report [60].

### Cell culture conditions, cell morphology, and cytotoxicity assay

HeLa cells were procured from ATCC (Manassas, VA, USA) and cultured in standard Dulbecco's Modified Eagles' Medium (DMEM) supplemented with 10% heat-inactivated fetal bovine serum (FBS), 1X solution of antibiotic-antimycotic (Thermo Fisher, India) and maintained at 37°C in a humidified atmosphere containing 5% (v/v) CO_2_. For testing the cytotoxicity (antiproliferative) potential of KP1019, Rapamycin (RAP), and metal cation chlorides (Al^3+^, Ca^2+^, Cd^2+^, Co^2+^, Cu^2+^, Fe^2+^, Mg^2+^, Mn^2+^, Na^+^, Ni^2+^, and Zn^2+^) in alone or combination, we performed standard MTT [3-(4,5-dimethylthiazol-2-yl)-2,5diphenyltetrazolium bromide] cell viability assay as described earlier [118]. Briefly, HeLa cells were seeded in a 96-well plate at a density of 7000 cells/well and left undisturbed for 24h. The stock solutions of KP1019 and RAP were prepared in DMSO, whereas metal chlorides in distilled water. The doses of KP1019, RAP and metal chlorides at which 85% of the cells will be viable upon individual treatment, were selected and added in duplicate to the 96-well plate in alone or combination. After 36h of incubation, the medium was replaced with an equal volume of fresh DMEM containing a final 0.2mg/ml of MTT reagent (Calbiochem, India) and incubated for 4h. The resultant formazan crystals were dissolved in DMSO solvent and the absorbance values were recorded at 570 and 690nm.

The viability of cells was calculated as % of control as following: % of control= [(OD_t_-OD_b_)/(OD_c_-OD_b_)]^*^100. ‘OD_t_’ represents the mean absorbance of treated cells at 570nm, ‘OD_c_’ represents the mean absorbance of solvent (DMSO) treated control cells at 570nm, and ‘OD_b_’ represents the mean absorbance of respective well at 690nm. Additionally, the abovementioned treatments were repeated in a 24-well plate (40,000cells/well). After 36h, the morphology of HeLa cells was recorded using an inverted microscope (Axio Vert.A1; Zeiss).

### Strains, chemicals, growth media, and growth conditions

The *Saccharomyces cerevisiae* strains used in this study were listed in Supplementary Table 1. Both the yeast knockout (YKO) collection and histone H3/H4 library derived by Dai et al. [63] were purchased from Open Biosystems. Unless stated otherwise, all yeast strains used in this study were grown at 30°C in standard synthetic complete (SC) liquid media containing 2% glucose. SC liquid media was prepared by mixing all amino acids, yeast nitrogen base (YNB) and ammonium sulfate (AS) together by following a standard protocol (Yeast Protocols Handbook, Clontech laboratories, Inc.). The wild-type yeast cells were transformed with *pPW344 (URA3), pHL126 (URA3)* plasmids using the standard lithium acetate procedure [119] and the resultant transformants were propagated in SC (Uracil dropped) liquid media. For solid agar media, 2% Bacto-agar was used in addition to SC media components. Media components and all other reagents used in this study were of molecular biology grade and purchased from Sigma–Aldrich, Merck, Himedia, GE Healthcare, Invitrogen, New England BioLabs and Thermo Fisher Scientific. Some of the metal chlorides were from Rankem, India.

### Growth sensitivity and growth curve assays

Growth sensitivity assay was performed to examine the effect of KP1019 on the growth of yeast cells as described previously [120]. In brief, overnight cultures of wild-type (WT) and mutant yeast cells were ten-fold serially diluted and 3μl of each was spotted onto solid SC-agar plates without (DMSO control) or with addition of KP1019, ethanolamine (ETA), rapamycin (RAP), sorbitol, reduced glutathione (GSH), N-acetylcysteine (NAC), and metal cation chlorides alone or in combination at indicated doses. All the plates were incubated at 30°C and growth was recorded after 72h using HP scanner.

For growth curve analysis, exponentially growing yeast cells were treated in duplicate with either DMSO (control) or indicated doses of KP1019, ETA, and GSH alone or in combination and then seeded in a 96-well cell culture plate (SPL Life Sciences Ltd.). Growth curves were constructed for each treatment using representative optical density (OD_600_) values measured at a regular interval of 30min for indicated period using a plate reader (Eon™ Microplate Spectrophotometer) [121].

### Total RNA isolation and global transcriptome analysis

The exponentially growing wild-type yeast (1588-4C) cells were treated with KP1019 (50μg/ml) or equivalent DMSO (solvent control) for 3h and then harvested. Total RNAs were isolated by heat/freeze Phenol method as described earlier [122] and assessed its integrity and quality by Agilent 2100 Bioanalyzer (Agilent Technologies, CA) prior to proceeding for gene expression profiling using Affymetrix microarray platform at iLife Discoveries (Gurgaon, India) [121]. Briefly, biotinylated complementary RNA (cRNA) was prepared from 6μg of total RNA from two independent biological repeats of each DMSO and KP1019 treated cells using *in vitro* transcription reaction and then hybridized to Yeast Genome 2.0 GeneChip Arrays (GPL2529) by following a standard Affymetrix protocol. Afterward, GeneChips were washed, stained in the Fluidics Station 450 (Affymetrix) and scanned using the GeneArray 30007G microarray scanner. The data sets were extracted from all CEL (raw intensity) files and submitted to NCBI's Gene Expression Omnibus (GEO) repository with a GEO Series accession number of GSE76985 (https://www.ncbi.nlm.nih.gov/geo/query/acc.cgi?acc=GSE76985).

The raw signals (CEL) in each array were processed for background adjustment, normalization followed by log transformation and summarization of probe sets using RMA (Robust Multi-array Average) algorithm in GeneSpring GX 12.6 expression analysis software (Agilent Technologies, CA). Differentially expressed genes (DEG’s) whose expression altered (induced or repressed) significantly by 1.5-fold (>1.5) in KP1019 treatment compared to DMSO (control) were determined by moderated t-test (p<0.05). Functional classification and Gene Ontology (GO) enrichment analysis of DEG's were performed using standard tools described in the supplementary information.

### Reverse transcriptase-pcr (RT-PCR)

Total cellular RNAs were extracted from yeast cells by heat/freeze phenol method as described earlier [122]. 1μg of DNA-free RNA was reverse transcribed to cDNA as per method supplied by iScript cDNA Synthesis Kit (Bio-Rad, India). The cDNA was amplified by PCR using Taq DNA Polymerase, reverse and forward primers of *HAC1-S* (**F**: 5’-TAGAGGGATTTCCAGAGCACG-3’; **R**: 5’-TCATTGAAGTGATGAAGAAATC-3’) and *ACT1* (**F**: 5’-CACCCTGTTCTTTTGACTGAAGC-3’; **R**: 5’-TACCGGCAGATTCCAAACCC-3’). The PCR amplicon products were electrophoresed, stained with ethidium bromide, and photographed [123].

### Preparation of protein extracts and immunoblotting analysis

Whole-cell protein extracts were obtained by 20% Trichloroacetic acid (TCA) precipitation method as described previously [124]. The protein extracts were resolved by electrophoresis on a SDS-polyacrylamide gel and transferred to nitrocellulose membranes. Immunoblotting analysis was performed by following a standard protocol. Briefly, the nitrocellulose membranes were blocked for 45min using blocking buffer (2.5% Bovine serum albumin in TBST; TBS containing 0.05% Tween-20) followed by incubation with primary antibodies for 90min (overnight for α-S758-P at 4°C). After washing with TBST, the membranes were incubated with relevant secondary antibody such as IRDye 800CW Goat anti-Rabbit IgG or anti-Mouse IgG (1:15000, LI-COR Biosciences) for 45min. Following primary antibodies were used: Anti-Rnr1 (Agrisera, ASO9576), Anti-Rnr2 (Agrisera, ASO9 575), Anti-Sml1 (Agrisera, AS10847), Anti-Rad53 (Santa Cruz Biotechnology Inc., SC-6749), Anti-Mpk1 (Santa Cruz Biotechnology Inc., SC-6803), and Anti-Phospho-p44/42 MAPK antibody (Cell Signaling, 4370). Anti-S758-P (Sch9-P) antibody was a kind gift from Prof. Robbie J. Loewith (University of Geneva, Switzerland). Polyclonal antibodies against recombinant Tbp and Rap1 were raised in rabbit. Blots were scanned by using Odyssey infrared imager (LI-COR Biosciences). Anti-Rad53 western signals were detected by chemiluminescence using Fuji gel-dock system (LAS-4000 mini).

### *β*-galactosidase activity assay

The *β*-galactosidase assay was performed to monitor the expression levels of *UPRE-lacZ* reporter gene as described previously [125]. Briefly, the exponentially growing wild-type (BY4741) yeast cells (OD_600_: 0.8-1) carrying 2μ *UPRE-lacZ* reporter plasmid (*pPW344*; gifted by Laran T. Jensen) [126] were treated with either DMSO (solvent control) or indicated doses of KP1019 (50, 100 μg/ml; 3h) and tunicamycin (1μg/ml; 1h) in SC (Ura dropped) media. Prior to harvesting the cells, the cell density at OD_600_ was determined. The *β*-galactosidase activity was measured by permeabilizing the cells in 1ml of Z buffer and using ONPG (ortho-Nitrophenyl-*β*-D-galactoside; 4mg/ml) as a substrate. The galactosidase activity was expressed in terms of Miller units as described earlier [127].

### Lipid droplets (LDs) visualization and ApoTome microscopy

To investigate the effects of KP1019 on neutral lipids (LDs), ER architecture, and Sfp1 localization in yeast, we employed BODIPY 493/503 dye [128], cells harbouring *ss-dsRed-HDEL* reporter (*GSHY583*; stains cortical and nuclear ER) [46], and *pHL126* plasmid (*pRS416-GFP-SFP1*; gifted by David Shore) [129], respectively. Exponentially growing yeast cells were left untreated (DMSO control) or treated with KP1019 (25, 50μg/ml) for 3h and 6h. The cells were incubated with 5μM of BODIPY 493/503 for 5min and washed with 1X-PBS (pH 7.4). The cells were imaged using the 63X oil-immersion objective lens of ZEISS ApoTome.2 microscope provided with the appropriate filter. Similarly, the wild-type (BY4743) cells carrying *pHL126* plasmid were grown till exponential phase (OD_600_: 1-1.2) and left untreated (DMSO control) or treated with KP1019 (50, 75μg/ml). Then the localization of Sfp1 was analyzed after 1h and 3h of KP1019 treatment using 63x oil-immersion objective lens of ZEISS ApoTome.2 microscope provided with the appropriate filter.

To test the effect of KP1019 on LDs in mammalian cells, HeLa cells were seeded in two-chambered slides (30,000 cells/well) using DMEM medium and left undisturbed for 24h. The cells were left untreated (DMSO control; final concentration <0.5%) or treated with KP1019 (20, 30μM) for 36h. The cells were then stained with 5μM of BODIPY 493/503 dye after 30min fixation with 3.7% formaldehyde as indicated earlier [130]. The cells were imaged using the 40X objective lens of ZEISS ApoTome.2 microscope provided with the appropriate filter. All the images were processed via ZEN-2012 (Blue edition) software. The quantitation of LDs both in yeast and HeLa cells was performed by counting the BODIPY 493/503 stained LDs in ApoTome images and represented as the number of LDs per cell.

### Measurement of Ruthenium (Ru) content by Inductively Coupled Plasma Mass Spectrometry (ICP-MS)

To assess the uptake of KP1019 by yeast cells, intracellular Ru levels were determined using ICP-MS as described previously [131]. A detailed description is provided in the supplementary information.

### Screening, validation, scoring, and functional analysis of H3/H4 mutant library

All the synthetic yeast histone H3/H4 library mutants were pre-cultured in SC liquid medium (Uracil dropped) till the saturation in a 96-well plate. The cell density of cultures was normalized to an OD_600_ of 0.1, and then replica spotted onto SC-agar tray plates that were supplemented without (DMSO control) or with 50μg/ml of KP1019 (for sensitivity), 100μg/ml of KP1019 (for resistance). The plates were incubated at 30°C and imaged after 48h. The growth fitness of mutants was compared to their respective wild-type cells spotted on the same plate and also to their corresponding fitness on the control (DMSO) plate. The mutants with decreased and increased fitness (in the presence of KP1019) compared to wild-type cells were assigned as KP1019 ‘sensitive’ and ‘resistant’ respectively. For further confirmation, KP1019 sensitive and resistant mutants were validated by both the growth assays and growth curves as described above.

Each of KP1019 sensitive and resistant mutants was scored using the similar methodology described by Rizzardi et al. [132]. Briefly, those mutants exhibiting increased or decreased growth fitness (in the presence of KP1019) relative to wild-type controls were designated positive or negative scores respectively from 2 to 4, with 2 being approximately a 10-fold difference in growth and 4 being a 1000-fold difference in growth.

For assessing the functional role of histone H3/H4 residues whose mutants exhibited altered KP1019 tolerance, we extracted information about their position in the nucleosome, status of PTM, and associated phenotypes (along with scores) from the HistoneHits database [64]. The histone H3/H4 residues whose mutants exhibited sensitivity to KP1019 were highlighted in the 3D yeast nucleosome structure (PDB ID: 1ID3) and visualized using PyMOL software.

### Human homologs of yeast KP1019 target genes and pathway analysis

The target genes of KP1019 that were obtained here through yeast growth assays were used to identify the corresponding human homologs and their role in different human diseases using YeastMine tool [133]. Diseases annotations for human genes are provided according to the OMIM (Online Mendelian Inheritance in Man) database. The biological functions associated with the human homologs of yeast genes were analyzed using ToppCluster tool [134].

### Statistical analysis

All the quantitative results are shown as Mean±SEM. The details about the number of independent experiments and experimental repeats are provided in the corresponding figure legends. Unless otherwise stated, statistical significance was assessed by performing Student's *t*-test (two-tailed; paired). *P*<0.05 considered as significant compared to control.

## SUPPLEMENTARY MATERIALS FIGURES AND TABLES















## Abbreviations

KP: KP1019
PTMs: post-translational modifications
SC: synthetic complete medium
OD600: optical density measured at 600nm
DEGs: differentially expressed genes
DMSO: dimethyl sulfoxide
RT-PCR: reverse transcriptase- polymerase chain reaction
ICP-MS: inductively coupled plasma mass spectrometry
Ru: Ruthenium
GO: gene ontology
MTT: [3-(4
CBBR: Coomassie brilliant blue R-250
TCA: trichloroacetic acid
MAPK: Mitogen-activated protein kinases
ROS: reactive oxygen species
RNR: ribonucleotide reductase
CWI: cell wall integrity
HOG: high osmolarity glycerol
ER: endoplasmic reticulum
LDs: lipid droplets
UPR: unfolded protein response
PE: Phosphatidylethanolamine
PC: phosphatidylcholine
FAs: fatty acids
BSO: buthionine sulfoximine
SERCA: sarcoplasmic/endoplasmic reticulum Ca^2+^-ATPase
GSH: reduced glutathione
NAC: N-acetylcysteine
ETA: ethanolamine
MMS: methyl methanesulfonate
HU: hydroxyurea
TAG: triacylglycerols
SE: steryl esters
TLS: translesion synthesis
RAP: rapamycin
TOR: target of rapamycin
DTT: dithiothreitol
TM: tunicamycin
