# Supplementary material for: A systematic assessment of chemical, genetic, and epigenetic factors influencing the activity of anticancer drug KP1019 (FFC14A)
Source: Oncotarget. 2017 Sep 30;8(58):98426–54. doi: 10.18632/oncotarget.21416 (PMC5716741; doi:10.18632/oncotarget.21416)
Supplement: Supplementary file 1 [file oncotarget-08-98426-s001.pdf]

A systematic assessment of chemical, genetic, and epigenetic factors influencing the activity of anticancer drug KP1019 (FFC14A)

SUPPLEMENTARY MATERIALS

Functional enrichment analysis of KP1019 transcriptome

The differentially expressed genes (DEG's) obtained upon KP1019 treatment were systematically classified into MIPS (Munich Information Center for Protein Sequences) functional categories using Functional Catalogue Database (FunCatDB; available at http://mips.helmholtzmuenchen.de/funcatDB/) [1], and evaluated for enrichment of functional gene clusters using a web-based tool, FunSpec (http://funspec.med.utoronto.ca) [2]. Moreover, the functional associations including pathways, protein, and genetic interactions exist in KP1019 induced transcriptome were assessed using GeneMANIA tool (http://genemania.org/) [3]. The functional interactions among genes belonging to significantly enriched biological processes were represented in the form of an interactive hierarchy using GeneMANIA Cytoscape plugin (http://apps.cytoscape.org/apps/genemania) [4] of Cytoscape software [5]. Additionally, the enrichment of chromatin features in both the induced and repressed genes of KP1019 transcriptome were analyzed using ChromatinDB database (http://www.bioinformatics2.wsu.edu/ChromatinDB) [6].

UV-Vis absorption spectroscopy

To reason the enhanced or decreased KP1019 cytotoxicity in the presence of different metal ions, we assessed the possible interaction or binding of metal ions with KP1019 by using UV-Vis spectrophotometer. Briefly, the final reaction volume of each 2ml was prepared separately with distilled water (aqueous) or 100mM Potassium phosphate buffer (pH 7.4), with or without (blank control) containing 65µM of KP1019, 400µM of aqueous solutions of individual metal cation (Al^{3+}, Ca^{2+}, Co^{2+}, Cu^{2+}, Fe^{2+}, Mn^{2+}, Na^{+}, and Zn^{2+}) chlorides in alone or combination. The final reaction mixtures (both aqueous and buffered) were incubated at 37°C for 5h with occasional vortexing. Primary spectra of each reaction mixture was obtained in the range of 300-600nm by using UV-Visible Spectrophotometer (Cary 100, Agilent Technologies) and imported into OriginPro 8.0 software.

Measurement of total ruthenium (Ru) levels by inductively coupled plasma mass spectrometry (ICP-MS)

The uptake of KP1019 in yeast cells was assessed in terms of total Ru levels by ICP-MS as described earlier [7]. Briefly, the exponentially growing wild-type (BY4743) yeast cells in synthetic complete (SC) liquid media were left untreated (DMSO) or treated with KP1019 (25µg/ml), ethanolamine-ETA (2.5mM), Fe^{2+} (2.5mM), reduced glutathione-GSH (10mM), and Sorbitol (1M) in alone or combination. After 3h treatment, the cells were pelleted (~30 x 10^6 cells) at 4°C and washed twice with ice-cold Milli-Q water. The whole-cell samples were digested in double-distilled 65% nitric acid for 2h at 65°C and were then diluted to 2% (v/v) nitric acid with Milli-Q water. The content of Ru was determined by iCAP™ Q ICP-MS instrument featured with a high-performance quadrupole analyzer (Thermo Fisher Scientific, USA) in a class 10,000 clean lab with class 1000 clear zones. The sensitivity of the instrument was optimized using a standard multi-element tuning solution containing representative analytes of the entire mass range capable of being scanned by the instrument. The instrument was calibrated for ^{101}Ru and ^{102}Ru by constructing standard curves from a Ru standard (aqueous solution of $RuCl_3$ matrixed in 2% HNO_3). The method blank solution (reagent water containing sample matrix and carried through entire digestion process) was measured in between each sample to ensure no memory from previous sample and double-distilled HNO_3 (2% v/v) was used as a backwash during the measurements. The results were corrected for Ru levels of method blank and the total Ru levels were represented as ppb (parts per billion) per OD_{600} (10^6 cells).

Micrococcal nuclease (MNase) accessibility of chromatin

MNase assay was performed following a protocol described previously with certain modifications [8]. In brief, yeast cells from 100ml mid-log phase culture of both untreated and KP1019 treated (50µg/ml; 6h) were harvested and washed once with ice-cold water followed by pre-spheroplasting buffer (100mM Tris-HCl pH 8.8,

10mM DTT, 0.1% NaN_3). Spheroplasts were prepared by incubating in Spheroplast buffer (50mM Tris-HCl pH 7.5, 0.6M Sorbitol, 10mM DTT) containing 250U Lyticase (Sigma) at 37°C for 15min with occasional shaking. Then spheroplasts were washed thrice with Sorbitol wash buffer (50mM HEPES-KOH pH 7.5, 100mM KCl, 2.5mM $MgCl_2$, 0.4M Sorbitol). Spheroplasts were then suspended in spheroplast digestion buffer (50mM Tris-HCl pH 7.9, 5mM $CaCl_2$), divided into 100µl aliquots and digested with varying concentrations (0, 100, 200, 300, 500, and 1000U/ml) of MNase (New England Biolabs-NEB; M0247S) for 5min at 37°C. The digestion reactions were terminated by adding 20µl of pre-warmed stop solution (250mM EDTA, 5% SDS) followed by Proteinase K (10mg/ml) treatment at 50°C overnight. Samples were extracted once with phenol-chloroform and once with chloroform. Then samples were ethanol precipitated and DNA was resuspended in TE (10mM Tris-HCl pH 8.0, 1mM EDTA) containing 100µg/ml RNase A. Samples were incubated at 37°C for 1 h, electrophoresed on 1.2% agarose gel and stained with ethidium bromide.

REFERENCES

1. Ruepp A, Zollner A, Maier D, Albermann K, Hani J, Mokrejs M, Tetko I, Guldener U, Mannhaupt G, Munsterkotter M, Mewes HW. The FunCat, a functional annotation scheme for systematic classification of proteins from whole genomes. Nucleic acids research. 2004; 32:5539-5545.
2. Robinson MD, Grigull J, Mohammad N, Hughes TR. FunSpec: a web-based cluster interpreter for yeast. BMC bioinformatics. 2002; 3:35.
3. Warde-Farley D, Donaldson SL, Comes O, Zuberi K, Badrawi R, Chao P, Franz M, Grouios C, Kazi F, Lopes CT, Maitland A, Mostafavi S, Montojo J, et al. The GeneMANIA prediction server: biological network integration for gene prioritization and predicting gene function. Nucleic acids research. 2010; 38:W214-220.
4. Montojo J, Zuberi K, Rodriguez H, Kazi F, Wright G, Donaldson SL, Morris Q, Bader GD. GeneMANIA Cytoscape plugin: fast gene function predictions on the desktop. Bioinformatics. 2010; 26:2927-2928.
5. Shannon P, Markiel A, Ozier O, Baliga NS, Wang JT, Ramage D, Amin N, Schwikowski B, Ideker T. Cytoscape: a software environment for integrated models of biomolecular interaction networks. Genome research. 2003; 13:2498-2504.
6. O'Connor TR, Wyrick JJ. ChromatinDB: a database of genome-wide histone modification patterns for Saccharomyces cerevisiae. Bioinformatics. 2007; 23:1828-1830.
7. Hostetter AA, Miranda ML, DeRose VJ, McFarlane Holman KL. Ru binding to RNA following treatment with the antimetastatic prodrug NAMI-A in Saccharomyces cerevisiae and *in vitro*. Journal of biological inorganic chemistry. 2011; 16:1177-1185.
8. Pidoux A, Mellone B, Allshire R. Analysis of chromatin in fission yeast. Methods. 2004; 33:252-259.
9. Tsaponina O, Barsoum E, Astrom SU, Chabes A. Ixr1 Is Required for the Expression of the Ribonucleotide Reductase Rnr1 and Maintenance of dNTP Pools. Plos Genet. 2011; 7.
10. Tomar RS, Zheng S, Brunke-Reese D, Wolcott HN, Reese JC. Yeast Rap1 contributes to genomic integrity by activating DNA damage repair genes. The EMBO journal. 2008; 27:1575-1584.
11. Suresh HG, da Silveira Dos Santos AX, Kukulski W, Tyedmers J, Riezman H, Bukau B, Mogk A. Prolonged starvation drives reversible sequestration of lipid biosynthetic enzymes and organelle reorganization in Saccharomyces cerevisiae. Molecular biology of the cell. 2015; 26:1601-1615.
12. Sandager L, Gustavsson MH, Stahl U, Dahlqvist A, Wiberg E, Banas A, Lenman M, Ronne H, Stymne S. Storage lipid synthesis is non-essential in yeast. J Biol Chem. 2002; 277:6478-6482.

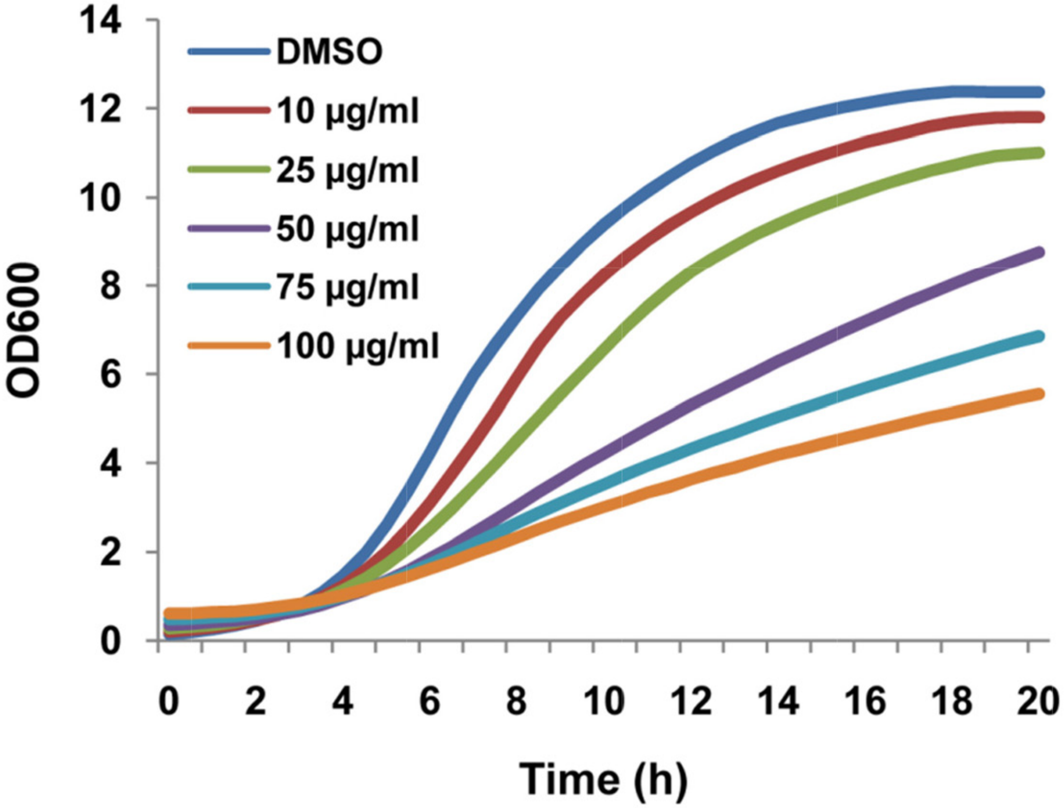

Supplementary Figure 1: KP1019 exhibit dose-dependent growth inhibition in yeast cells. KP1019 negatively affects the growth of wild-type yeast cells in a dose-dependent manner. The exponentially growing wild-type (W1588-4C) yeast cells were treated with either DMSO (control) or indicated doses of KP1019 in SC-liquid media and then growth was monitored in terms of absorbance (OD600) for 20h using a plate reader.

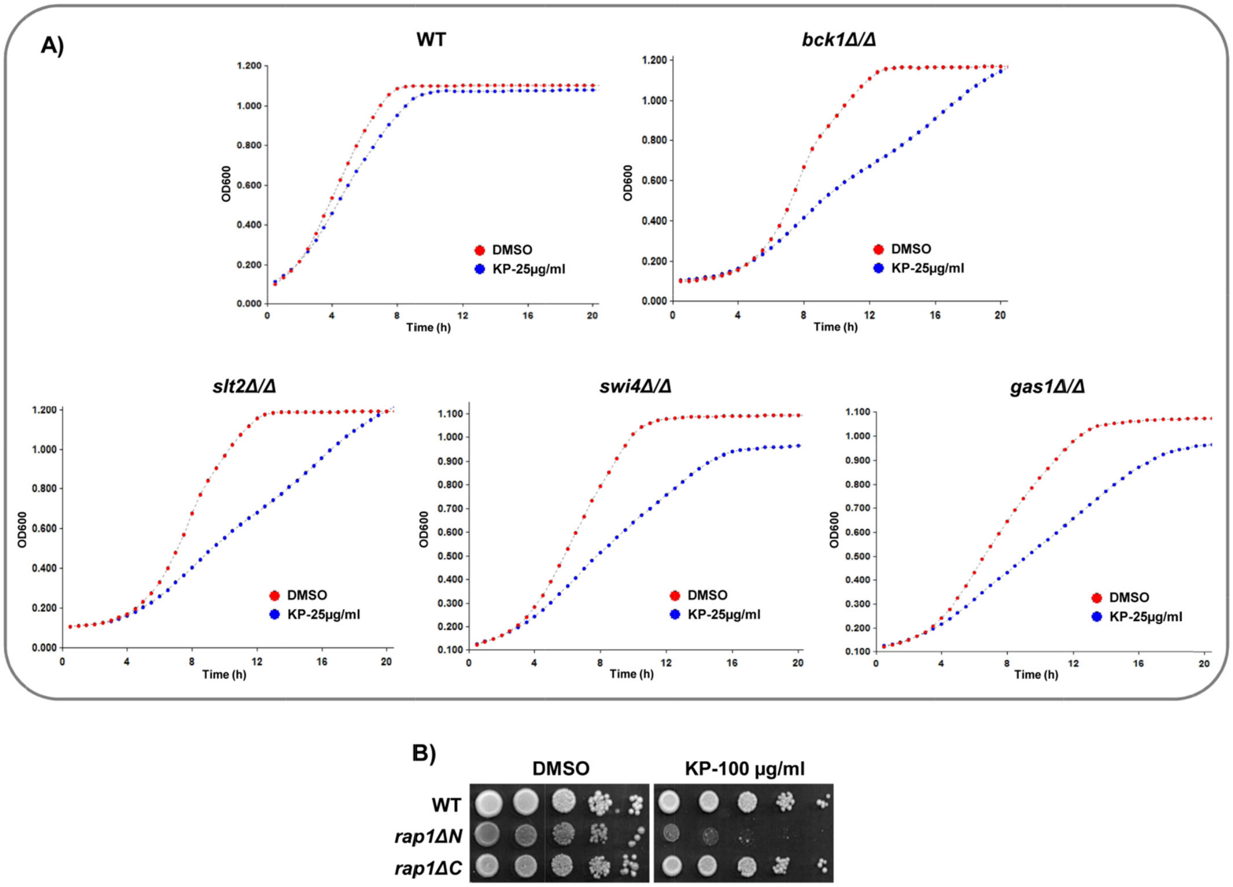

Supplementary Figure 2: Cell wall integrity (CWI) pathway mutant cells exhibit sensitivity to KP1019. (A) KP1019 exerts its cytotoxicity by altering CWI pathway in yeast. The exponentially growing wild-type (BY4743) and indicated CWI pathway null mutant cells were treated with either DMSO solvent (control) or KP1019 (25μg/ml) in SC-liquid media, and the growth was monitored in terms of absorbance (OD600) for 20h using a plate reader. **(B)** N-terminal of Rap1 is essential for KP1019 tolerance. 3μl of ten-fold serial dilutions of wild-type (WT) and Rap1 mutant cells were spotted onto SC-agar plates containing either KP1019 (100μg/ml) or equivalent DMSO solvent (control). The plates were incubated at 30°C and imaged after 72h.

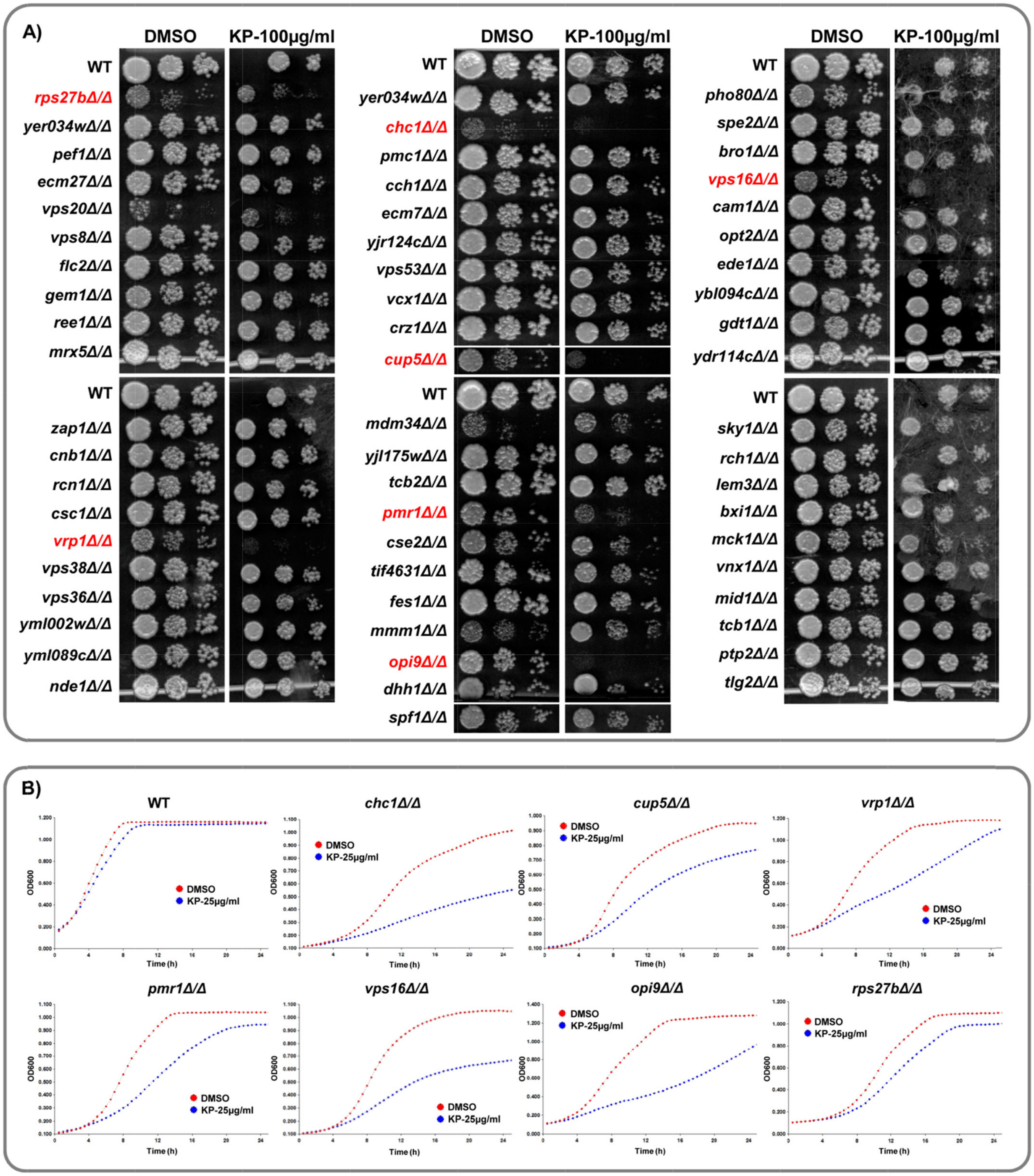

Supplementary Figure 3: Loss of Ca2+ ion homeostasis genes increase KP1019-induced toxicity. **(A)** Genetic screening of Ca^{2+} homeostasis null mutants for KP1019 tolerance. 3µl of ten-fold serial dilutions of wild-type (BY4743; WT) and indicated null mutant cells were spotted onto SC-agar plates containing either KP1019 (100µg/ml) or equivalent DMSO solvent (control). The plates were incubated at 30°C and imaged after 48h. **(B)** Validation of KP1019 sensitivity by growth curve analysis. The exponentially growing wild-type (BY4743) and Ca^{2+} homeostasis null mutant cells were treated with either DMSO solvent (control) or KP1019 (25µg/ml) and the growth was monitored in terms of absorbance (OD600) for 24h using a plate reader.

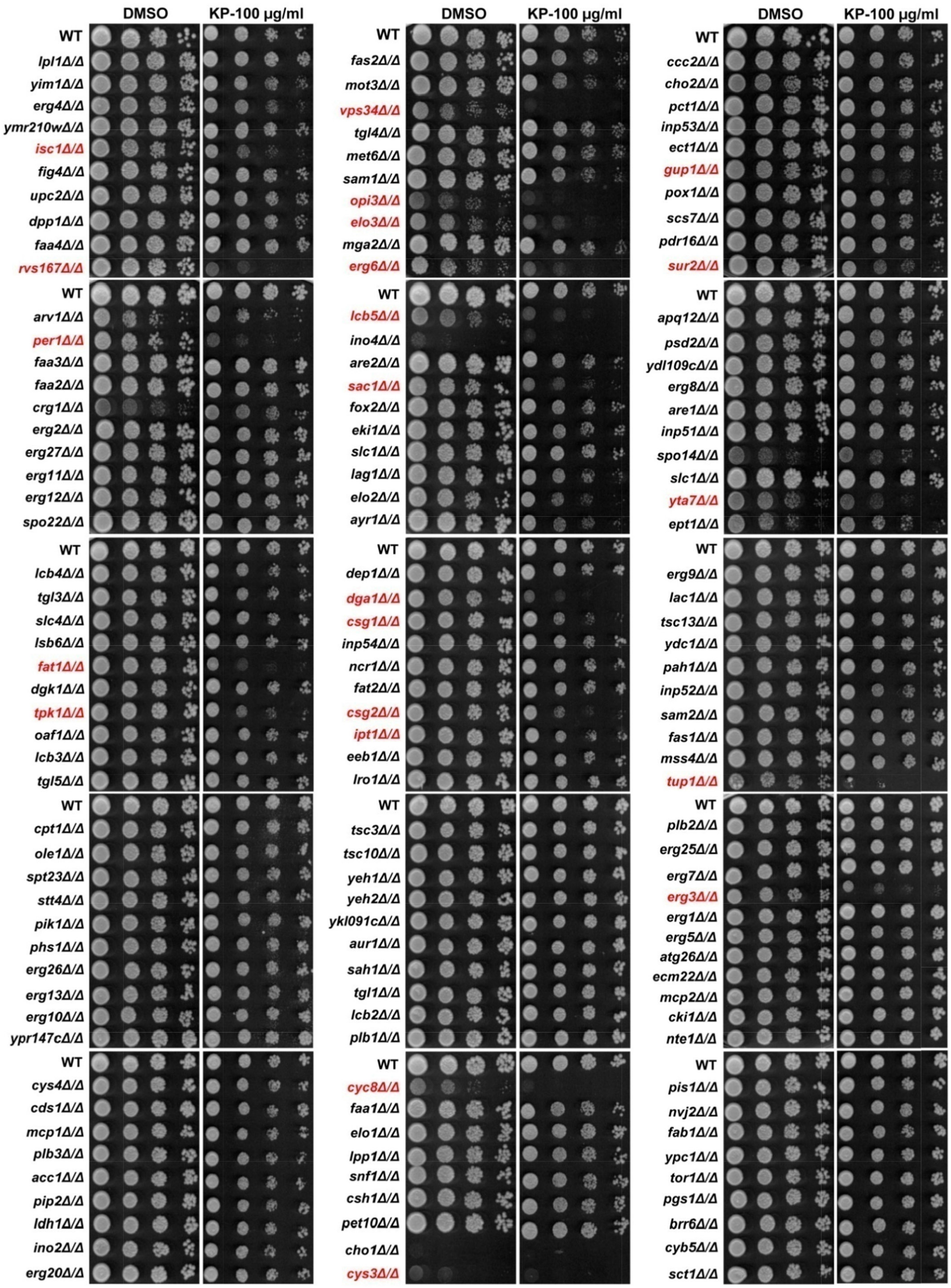

Supplementary Figure 4: Loss of lipid homeostasis genes increase KP1019-induced toxicity. Genetic screening of lipid homeostasis mutants for KP1019 tolerance. Growth assay of wild-type (BY4743; WT) and indicated mutant cells was performed in absence and presence of KP1019 (100µg/ml). Plates were imaged after 48h.

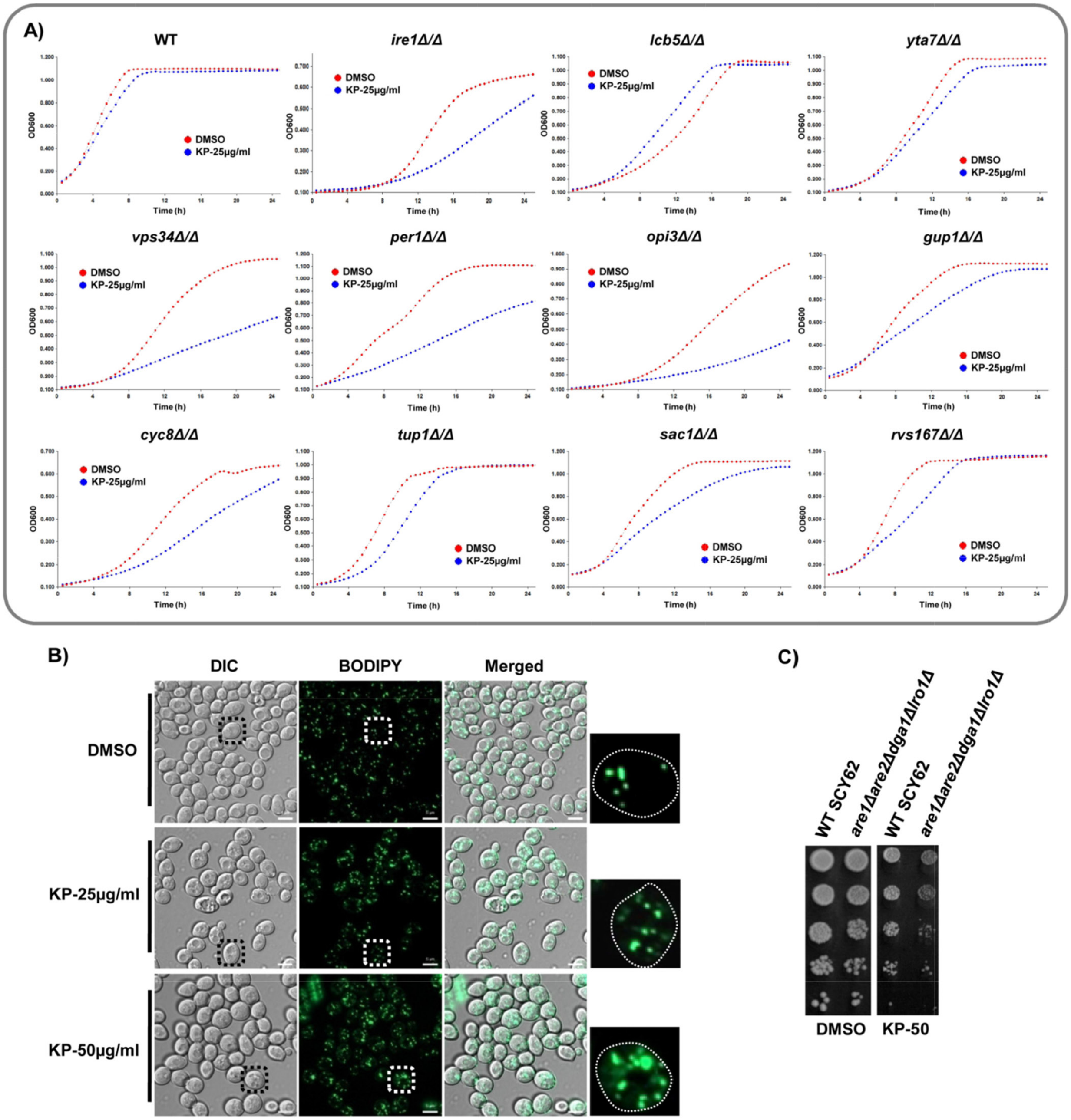

Supplementary Figure 5: KP1019 treatment alters lipid homeostasis in budding yeast cells. (A) The null mutants of lipid homeostasis genes exhibit sensitivity to KP1019. The exponentially growing wild-type (BY4743) and lipid homeostasis null mutant cells were treated with either DMSO solvent (control) or KP1019 (25µg/ml), and the growth was monitored in terms of absorbance (OD600) for 24h using a plate reader. **(B)** KP1019 treatment leads to the accumulation of lipid droplets (LDs). Exponentially growing wild-type (BY4743) yeast cells were treated with either DMSO solvent (control) or KP1019 (25 and 50µg/ml) for 3h and then incubated with 5µM BODIPY493/503 dye for 5min. The LDs were visualized under ApoTome microscope (40X objective) using FITC filter. Representative images from three independent experiments are shown. Scale bar represents 5µm. **(C)** The effect of KP1019 does not exacerbate in cells lacking LDs. Growth assay was performed with wild-type (SCY62) and quadruple null mutant cells that lacks LDs in absence and presence of KP1019 (50µg/ml). The plates were imaged after 48h.

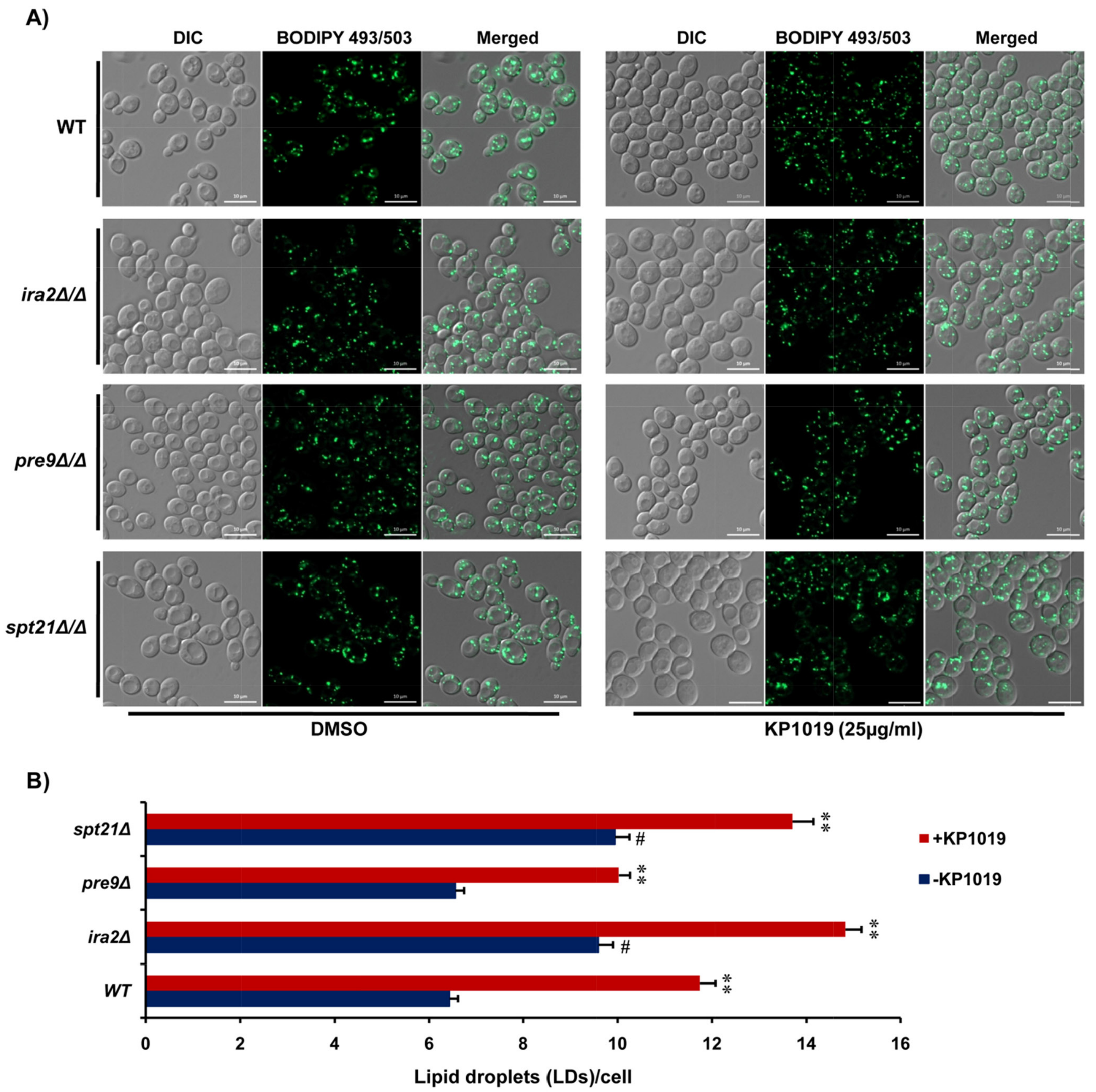

Supplementary Figure 6: Effect of KP1019 treatment in yeast mutants containing higher lipid content. (A) KP1019 treatment leads to increased cell size and number of lipid droplets (LDs) in yeast null mutants with higher lipid content. Exponentially growing wild-type (BY4743) and indicated yeast null mutants were treated with either DMSO solvent (control) or KP1019 (25µg/ml) for 3h and then incubated with 5µM BODIPY 493/503 dye for 5min. The LDs were visualized under ApoTome microscope (40X objective) using FITC filter. Representative images from two independent experiments were shown. Scale bar represents 10µm. **(B)** Quantification of KP1019-induced LDs formation in (A). The LDs stained with BODIPY 493/503 were counted in ApoTome images and represented as number of LDs per cell (n=150; Mean ± SEM) in wild-type (BY4743; WT) and indicated null mutants that were treated with either DMSO (solvent control; -KP1019) or KP1019 (25µg/ml; +KP1019) for 3h. **P<0.001 (compared to respective –KP1019) and $^{\#}$P<0.001 (compared to untreated WT) were considered significant (Student's t-test).

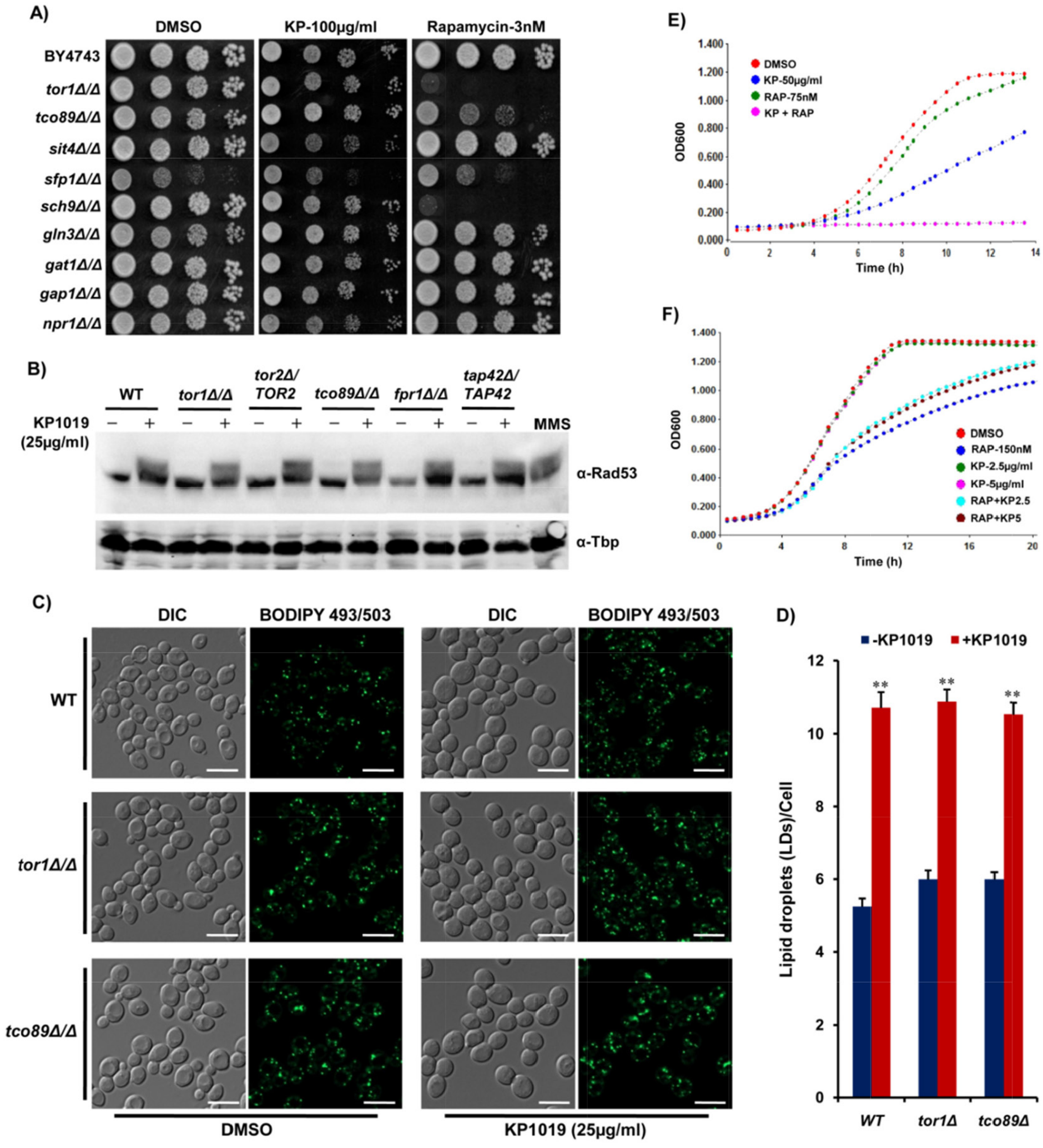

Supplementary Figure 7: Functional TOR pathway is not required for mediating KP1019-induced toxicity. **(A)** TOR pathway null mutants do not exhibit phenotypic alterations in presence of KP1019. Growth assay was performed with wild-type (BY4743) and indicated null mutants in absence or presence of KP1019 (100µg/ml) and Rapamycin (3nM). Rapamycin, an inhibitor of Tor1 served as a positive control. The Plates were imaged after 48h. **(B)** KP1019 activates Rad53 in null mutants of TOR pathway. The whole-cell protein extracts of wild-type (BY4743; WT) and indicated null mutants that were left untreated (DMSO control) or treated with KP1019 (25µg/ml) for 3h subjected to western blot analysis using the indicated antibodies. Anti-Tbp signals were used to check the protein loading. Cells treated with 0.03% methyl methanesulfonate (MMS) were served as positive control. **(C & D)** KP1019 induce the formation of LDs in TOR pathway null mutants. The wild-type and indicated null mutants of TOR pathway were left untreated (DMSO control) or treated with KP1019 (25µg/ml) for 3h and then stained with BODIPY 493/503 dye. Scale bar represents 10µm (C). The LDs stained with BODIPY 493/503 in (C) were counted and represented as number of LDs per cell (n=100; Mean ± SEM). **P<0.001 (compared to respective –KP1019) was considered significant (Student's t-test) (D). **(E & F)** The effect of combinatorial treatment with KP1019 and Rapamycin depends on their dose ratio. The exponentially growing wild-type (BY4743) cells were treated with either DMSO solvent (control) or indicated doses of KP1019 (KP) and rapamycin (RAP) in alone or combination. The growth was monitored in terms of absorbance (OD600) for indicated period using a plate reader.

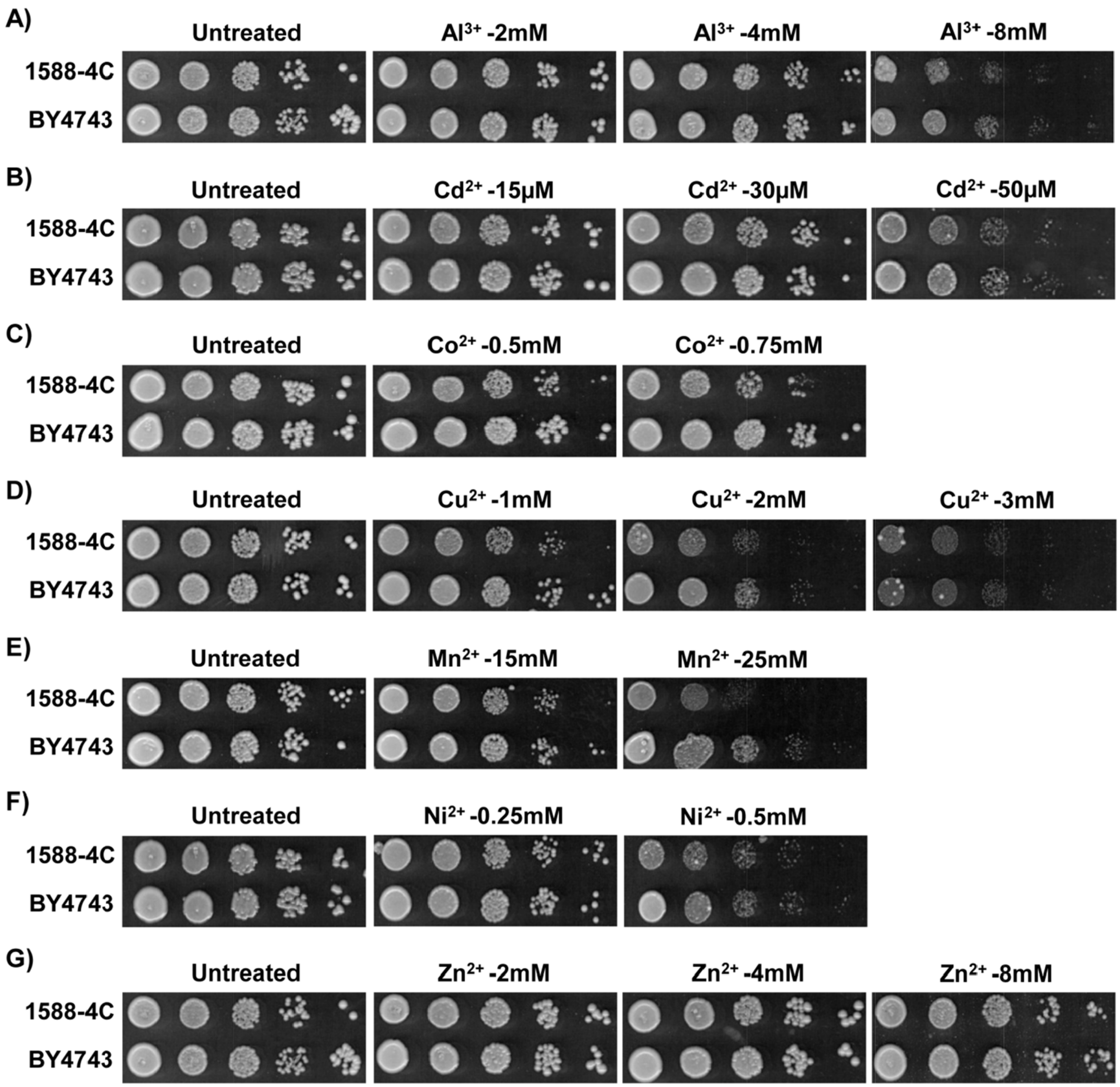

Supplementary Figure 8: Dose-dependent toxicity of various metal ions in budding yeast. **(A-G)** Screening of different metal cations (chlorides) to determine their tolerable dose. Ten-fold serial dilutions of wild-type (1588-4C and BY4743) cells were spotted onto SC-agar plates supplemented without or with indicated doses of different metal ion chlorides. The plates were incubated at 30°C and then imaged after 48h.

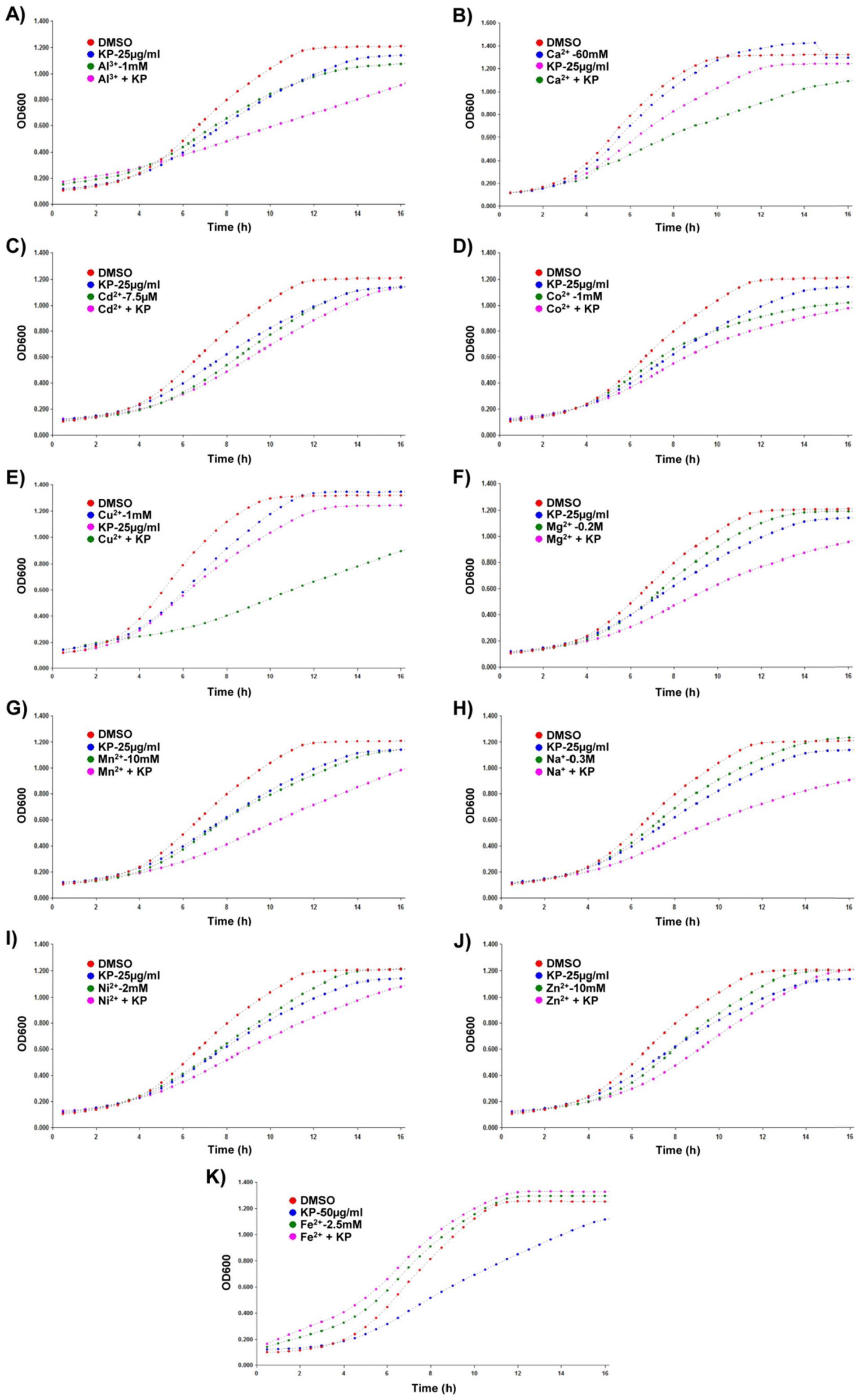

Supplementary Figure 9: KP1019 effect is modulated in the presence of different metal ions. **(A-K)** The effect of KP1019 is enhanced by Al^{3+}, Ca^{2+}, Cd^{2+} Cu^{2+}, Mn^{2+}, Na^{+}, and Zn^{2+}, whereas repressed by Fe^{2+} metal ions. The exponentially growing wild-type (BY4743) cells were treated with either DMSO solvent (control) or indicated doses of KP1019 and different metal cation chlorides in alone or combination. The growth was monitored in terms of absorbance (OD600) for 16h using a plate reader.

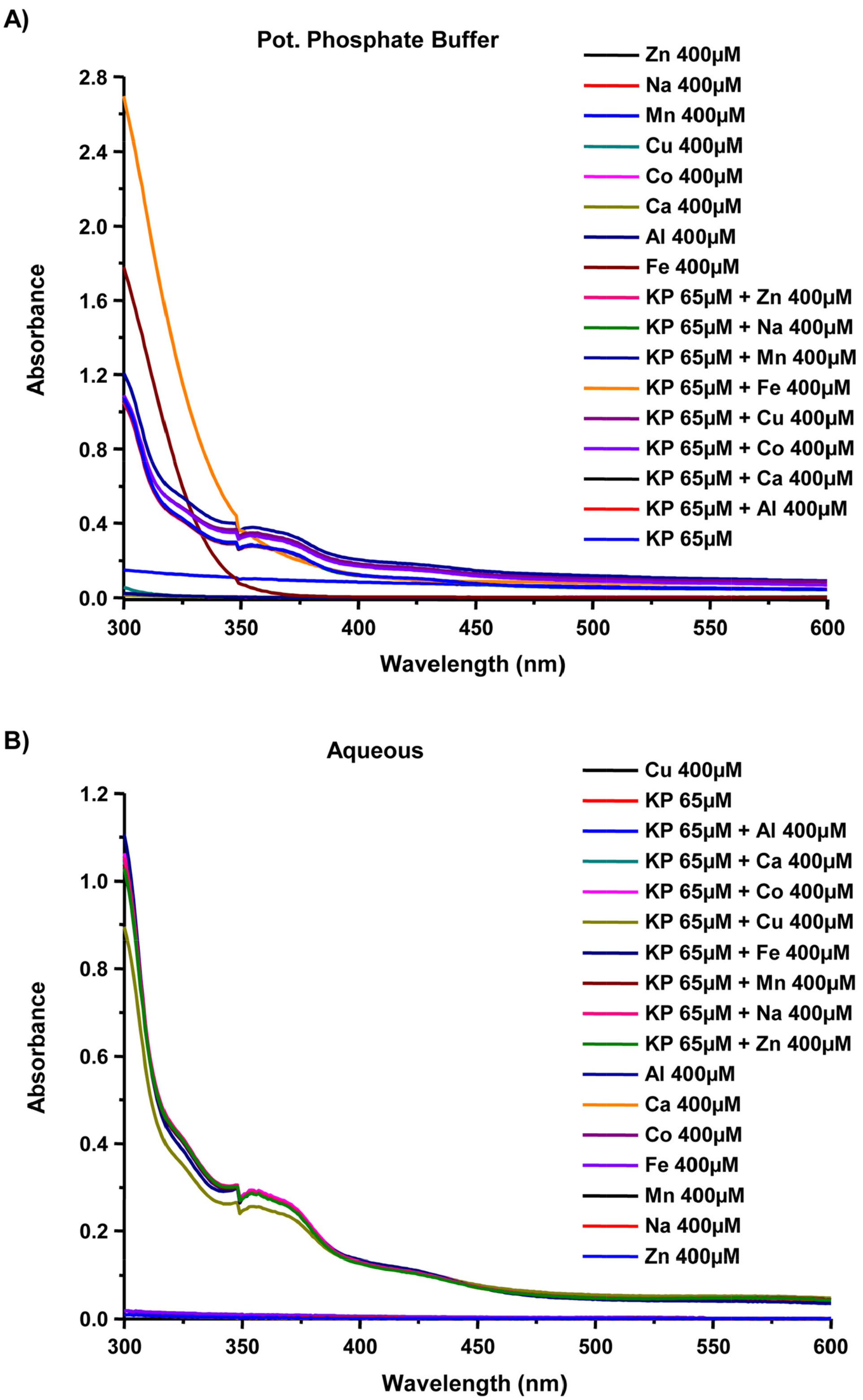

Supplementary Figure 10: UV-Vis absorption spectra of KP1019 in the presence of metal ions. **(A & B)** The absorption spectra of KP1019 doesn't alter in the presence of metal ions other than Fe^{2+}. The buffered (A) or aqueous (B) solutions of KP1019 (65µM) were incubated with 400µM of indicated metal cation chlorides for 5h at 37°C. The absorption spectra of KP1019, metal ions in alone or combination were obtained by using a UV-Vis spectrophotometer.

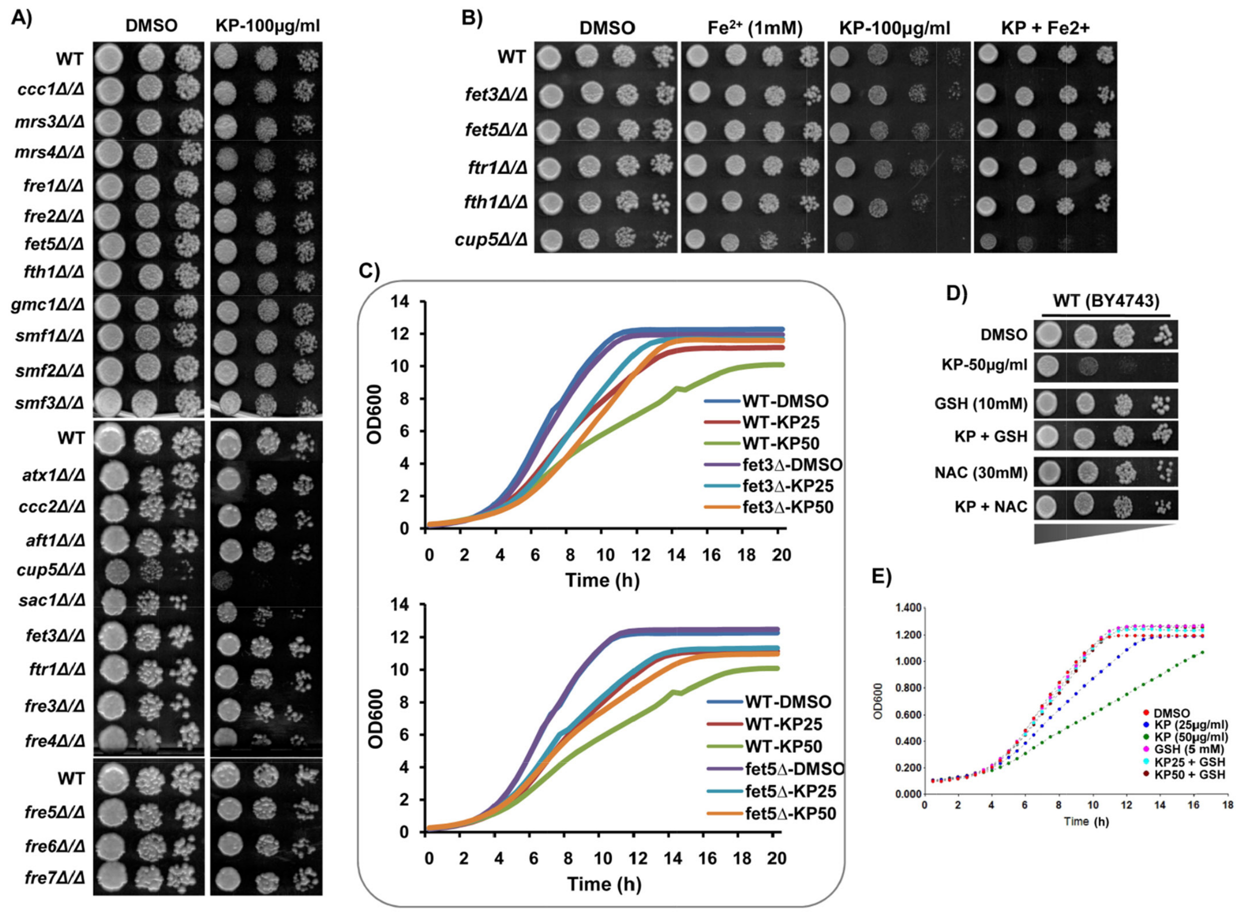

Supplementary Figure 11: Supplementation of Fe^{2+} ion and reduced glutathione (GSH) neutralizes the KP1019-induced toxicity. **(A)** KP1019 treatment does not alter cellular iron levels. Growth assay was performed with wild-type (BY4743; WT) cells and indicated iron homeostasis null mutants in the presence of DMSO (Solvent control) and KP1019 (100µg/ml). The Plates were imaged after 48h. **(B)** Supplementation of Fe^{2+} in the media remediates KP1019 induced toxicity. Ten-fold serial dilutions of wild-type (BY4743) cells and null mutants of iron transporter genes were spotted onto SC-agar plates supplemented without or with indicated doses of KP1019 and Fe^{2+} in alone or combination. The plates were incubated at 30°C and imaged after 48h. **(C)** Loss of Iron transporter genes increases the tolerance to KP1019. The exponentially growing wild-type (BY4743) cells and null mutants of iron-transport system (*fet3Δ/Δ, fet5Δ/Δ*) were treated with either DMSO solvent (control) or indicated doses of KP1019. The growth was monitored in terms of absorbance (OD600) for 20h using a plate reader. **(D)** Reduced glutathione (GSH) and its precursor N-Acetyl cysteine (NAC) neutralize KP1019-induced toxicity. Ten-fold serial dilutions of wild-type (BY4743) cells were spotted onto SC-agar plates supplemented without or with indicated doses of KP1019, GSH and NAC in alone or combination. The plates were incubated at 30°C and imaged after 48h. **(E)** Supplementation of GSH remediates KP1019-induced toxicity. The exponentially growing wild-type (BY4743) cells were treated with either DMSO solvent (control) or indicated doses of KP1019 and GSH in alone and combination. The growth was monitored in terms of absorbance (OD600) for 16h using a plate reader.

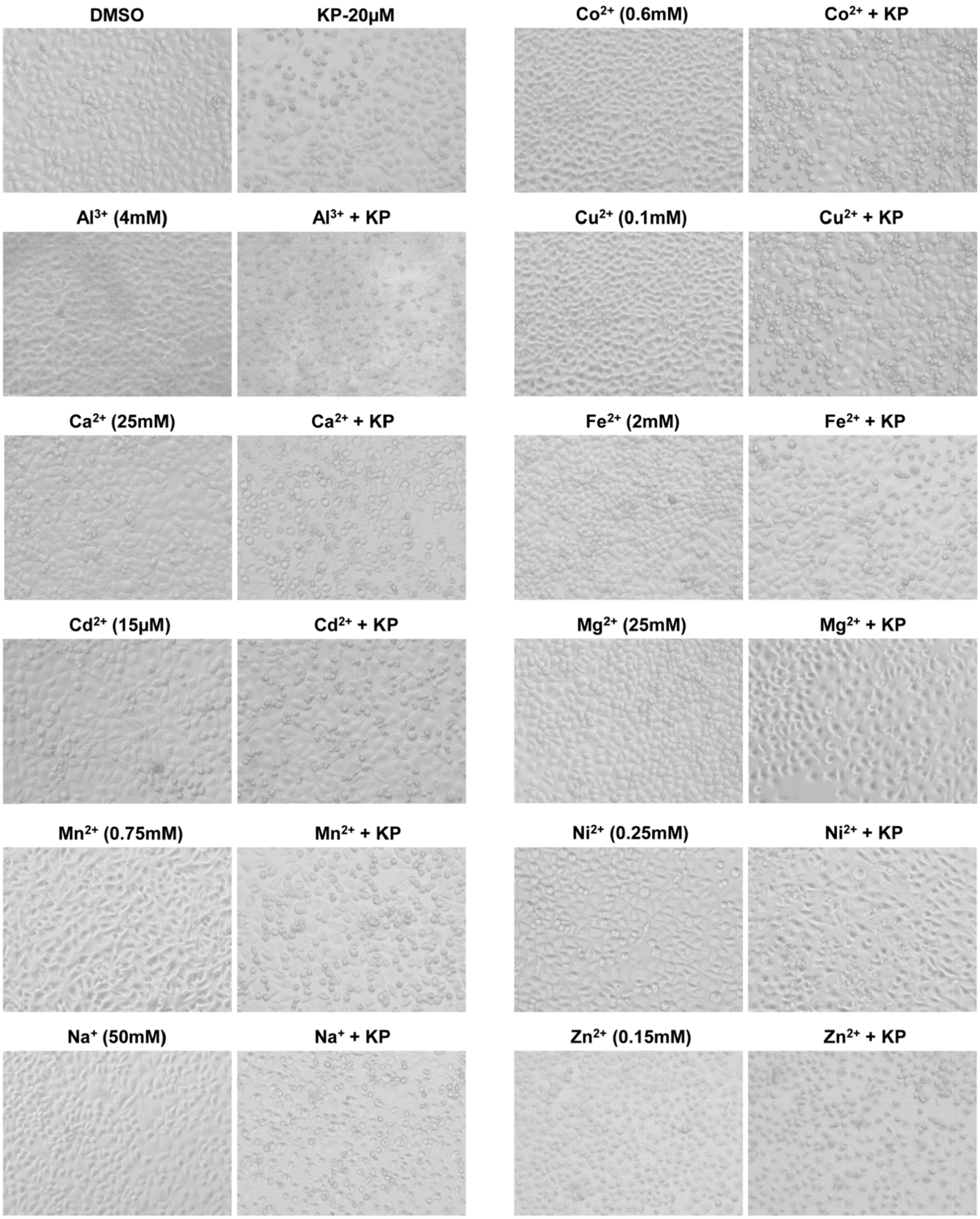

Supplementary Figure 12: Effect of KP1019 treatment in combination with metal ions on HeLa cells morphology. The co-treatment of KP1019 and metal ions has detrimental effects on HeLa cells morphology. Human cervical cancer cells (HeLa) were seeded in 24-well plate (40,000 cells/well). After 24h, the cells were treated with either DMSO (Solvent control) or indicated doses of KP1019, metal cation chlorides in alone or combination. The morphology of HeLa cells was imaged after 36h of treatment using 10X objective of an inverted microscope. Representative images from two independent experiments are shown.

A) Induced Transcriptome

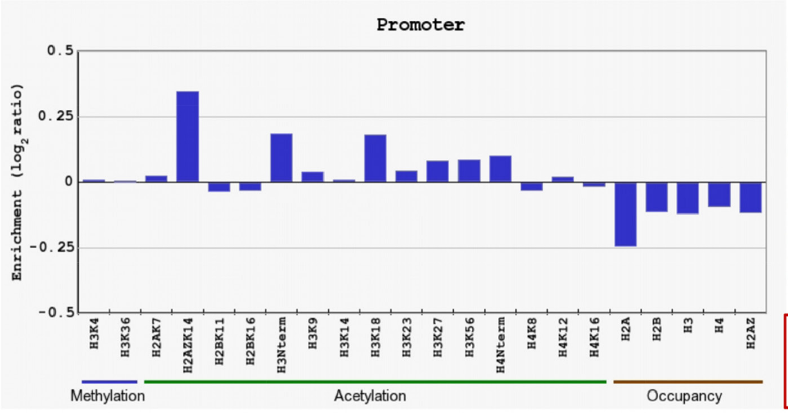

Chromatin Enrichment/Depletion
Promoter

Histone Modification	Rank Percentile	Trend	P-value
H3K4 Di-Methylation	49.9 %	none	0.9403
H3K36 Di-Methylation	52.6 %	none	0.1502
H2AK7 Acetylation	50.3 %	none	0.9187
H2AZK14 Acetylation	60.0 %	**Enriched**	$< 10^{-4}$
H2BK11 Acetylation	45.9 %	none	0.1501
H2BK16 Acetylation	47.2 %	none	0.3312
H3Nterm Acetylation	57.5 %	**Enriched**	$< 10^{-4}$
H3K9 Acetylation	50.5 %	none	0.8666
H3K14 Acetylation	48.3 %	none	0.5439
H3K18 Acetylation	53.5 %	none	0.2238
H3K23 Acetylation	50.3 %	none	0.9077
H3K27 Acetylation	52.3 %	none	0.4171
H3K56 Acetylation	55.7 %	none	0.0034
H4Nterm Acetylation	54.7 %	none	0.0098
H4K8 Acetylation	46.7 %	none	0.2457
H4K12 Acetylation	50.1 %	none	0.9786
H4K16 Acetylation	50.5 %	none	0.8749
H2A Occupancy	36.9 %	**Depleted**	$< 10^{-9}$
H2B Occupancy	39.5 %	**Depleted**	$< 10^{-8}$
H3 Occupancy	38.5 %	**Depleted**	$< 10^{-9}$
H4 Occupancy	39.8 %	**Depleted**	$< 10^{-7}$
H2AZ Occupancy	42.7 %	**Depleted**	$< 10^{-3}$

B) Repressed Transcriptome

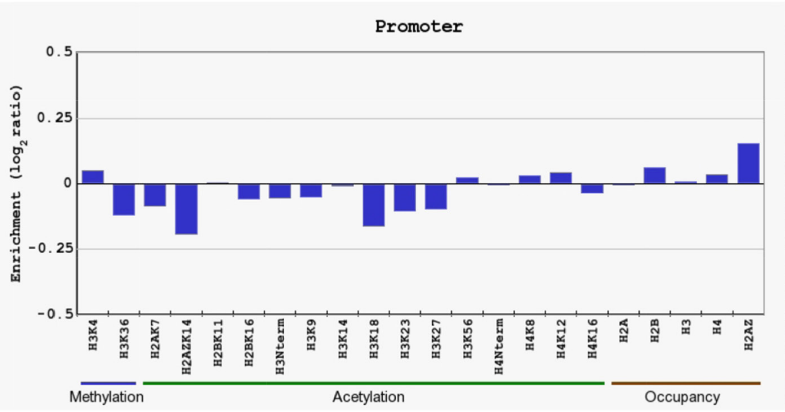

Chromatin Enrichment/Depletion
Promoter

Histone Modification	Rank Percentile	Trend	P-value
H3K4 Di-Methylation	52.8 %	none	0.3991
H3K36 Di-Methylation	43.2 %	none	0.0496
H2AK7 Acetylation	42.9 %	none	0.2054
H2AZK14 Acetylation	42.8 %	none	0.0938
H2BK11 Acetylation	48.8 %	none	0.8278
H2BK16 Acetylation	44.3 %	none	0.3054
H3Nterm Acetylation	45.0 %	none	0.1382
H3K9 Acetylation	46.9 %	none	0.5744
H3K14 Acetylation	48.0 %	none	0.7157
H3K18 Acetylation	40.5 %	none	0.0897
H3K23 Acetylation	42.2 %	none	0.1648
H3K27 Acetylation	42.7 %	none	0.1892
H3K56 Acetylation	50.8 %	none	0.8211
H4Nterm Acetylation	46.1 %	none	0.2397
H4K8 Acetylation	50.9 %	none	0.8665
H4K12 Acetylation	53.0 %	none	0.5871
H4K16 Acetylation	47.0 %	none	0.5869
H2A Occupancy	53.1 %	none	0.4041
H2B Occupancy	57.4 %	none	0.0296
H3 Occupancy	52.4 %	none	0.4915
H4 Occupancy	54.7 %	none	0.1677
H2AZ Occupancy	52.5 %	none	0.4818

Supplementary Figure 13: Genes induced by KP1019 treatment have depleted histone occupancy and enriched active histone post-translational modifications (PTMs) at the promoter. (A & B) Levels of Histone occupancy and histone PTMs in the promoter regions of 284 genes induced (A) and 76 genes repressed (B) by KP1019 treatment (i.e., genes that are repressed and induced in untreated cells respectively) were analysed (normalized by nucleosome occupancy levels) by using the web-based ChromatinDB database. Statistical analysis of the data (P-value) and the trend (enriched or depleted) followed by histone occupancy and PTMs were also represented.

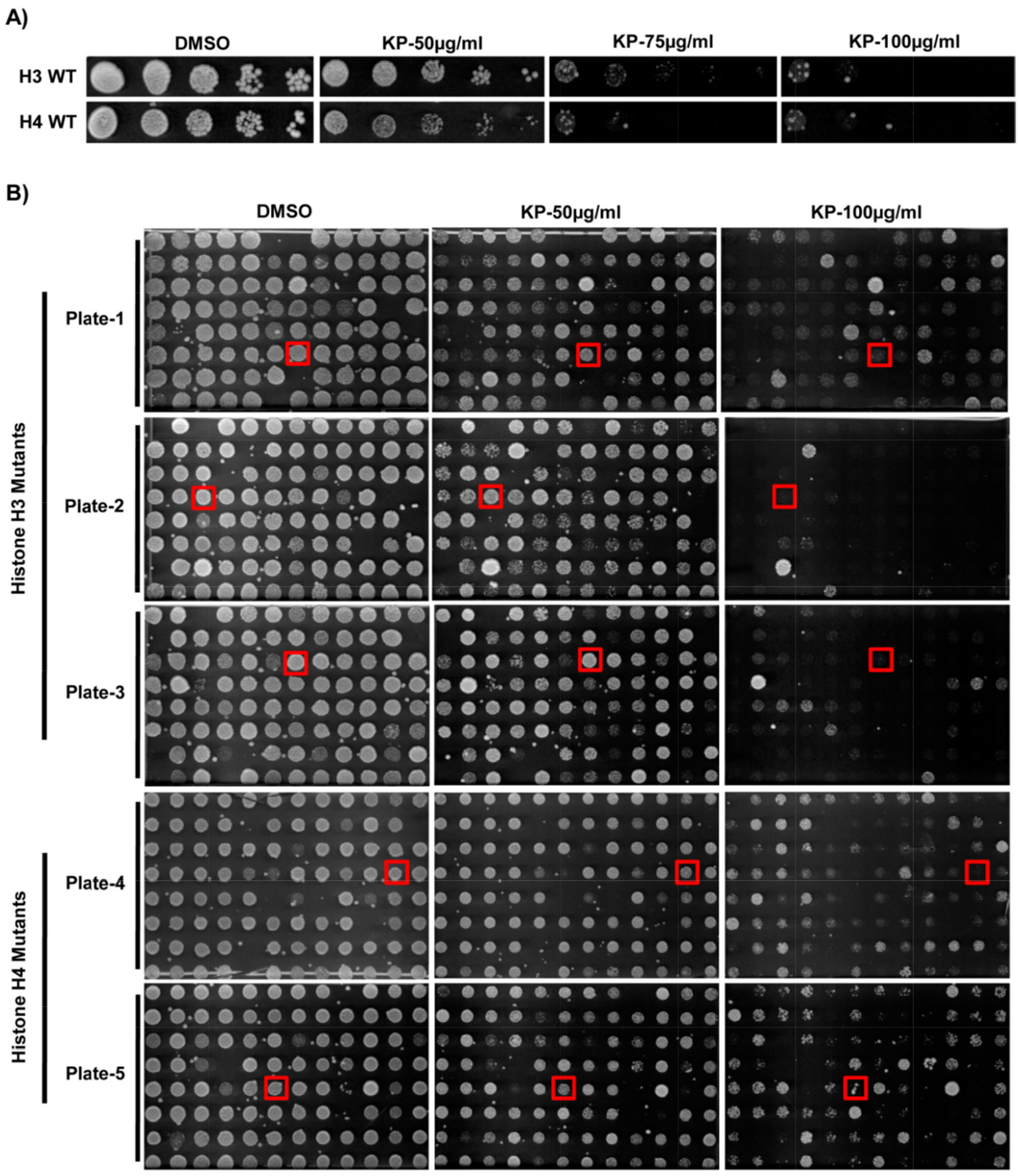

Supplementary Figure 14: High-throughput screening of synthetic histone H3/H4 mutant library for KP1019 tolerance. (A) The selection of KP1019 dose for screening the histone H3/H4 library. Ten-fold serial dilutions of wild-type (H3 and H4) cells were spotted onto SC-agar plates supplemented without or with indicated doses of KP1019. The plates were incubated at 30°C and imaged after 48h. **(B)** High-throughput screening of histone H3/H4 library revealed the role of histone H3 and H4 residues in KP1019 tolerance (sensitive and resistant). The library mutants grown in 96-well plates were normalized to an OD_{600} of 0.1 and then dropped onto the plates supplemented without (DMSO control) or with indicated doses of KP1019. The plates were incubated at 30°C and imaged after 48h. The position of wild-type spot in the plate was indicated by a red-colored square. The KP1019 dose of 50µg/ml and 100µg/ml was used to identify the sensitive and resistant mutants respectively.

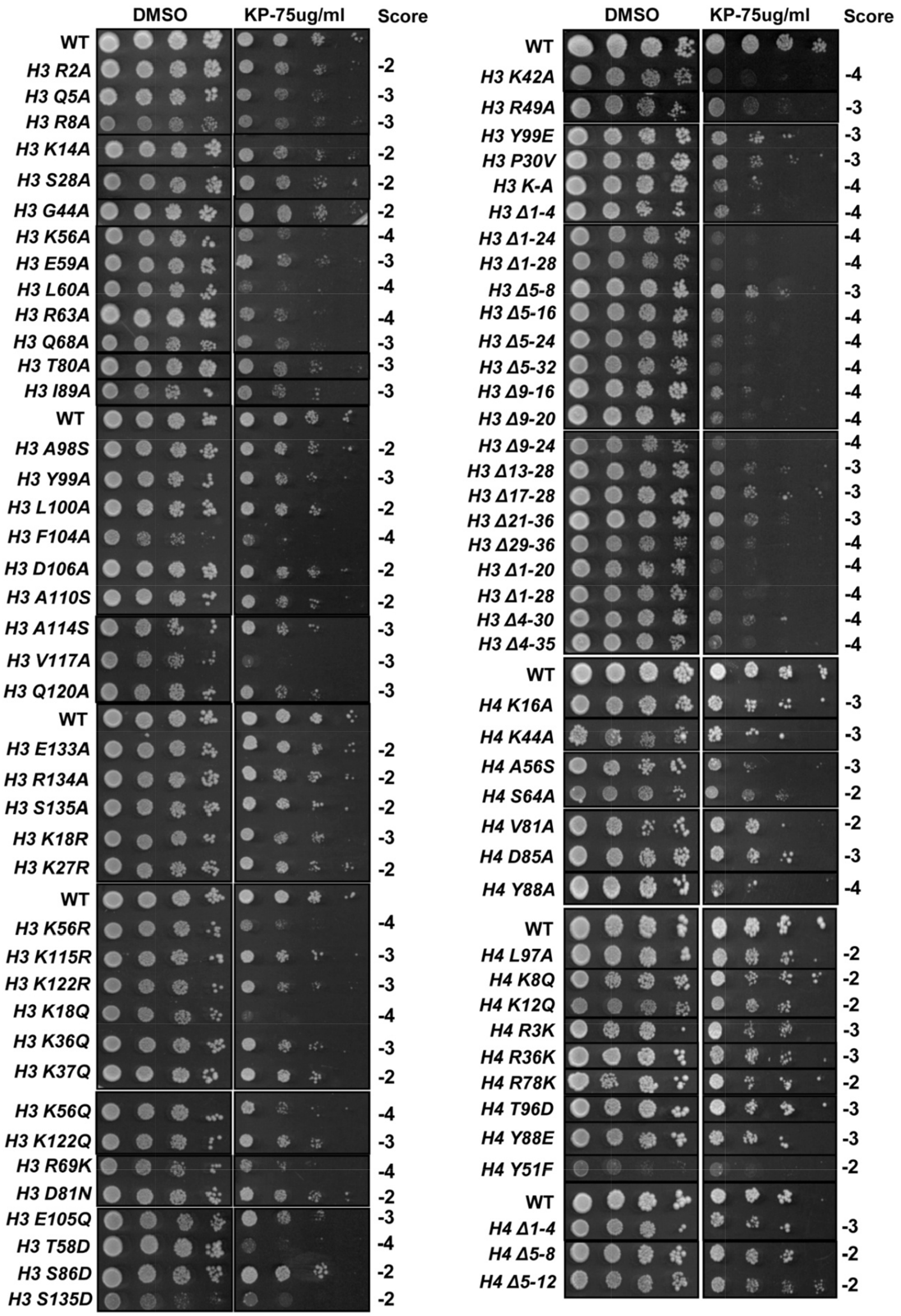

Supplementary Figure 15: Validation and scoring of KP1019 sensitive mutants obtained in high-throughput screening. Ten-fold serial dilutions of wild-type (H3 and H4; WT), mutants of histone H3 and H4 were spotted onto SC-agar plates supplemented without or with KP1019 (75µg/ml). The plates were incubated at 30°C and imaged after 48h. The scores for each of KP1019 sensitive mutants was given (-2 to -4) based on their growth fitness as described in the 'Materials and Methods' section.

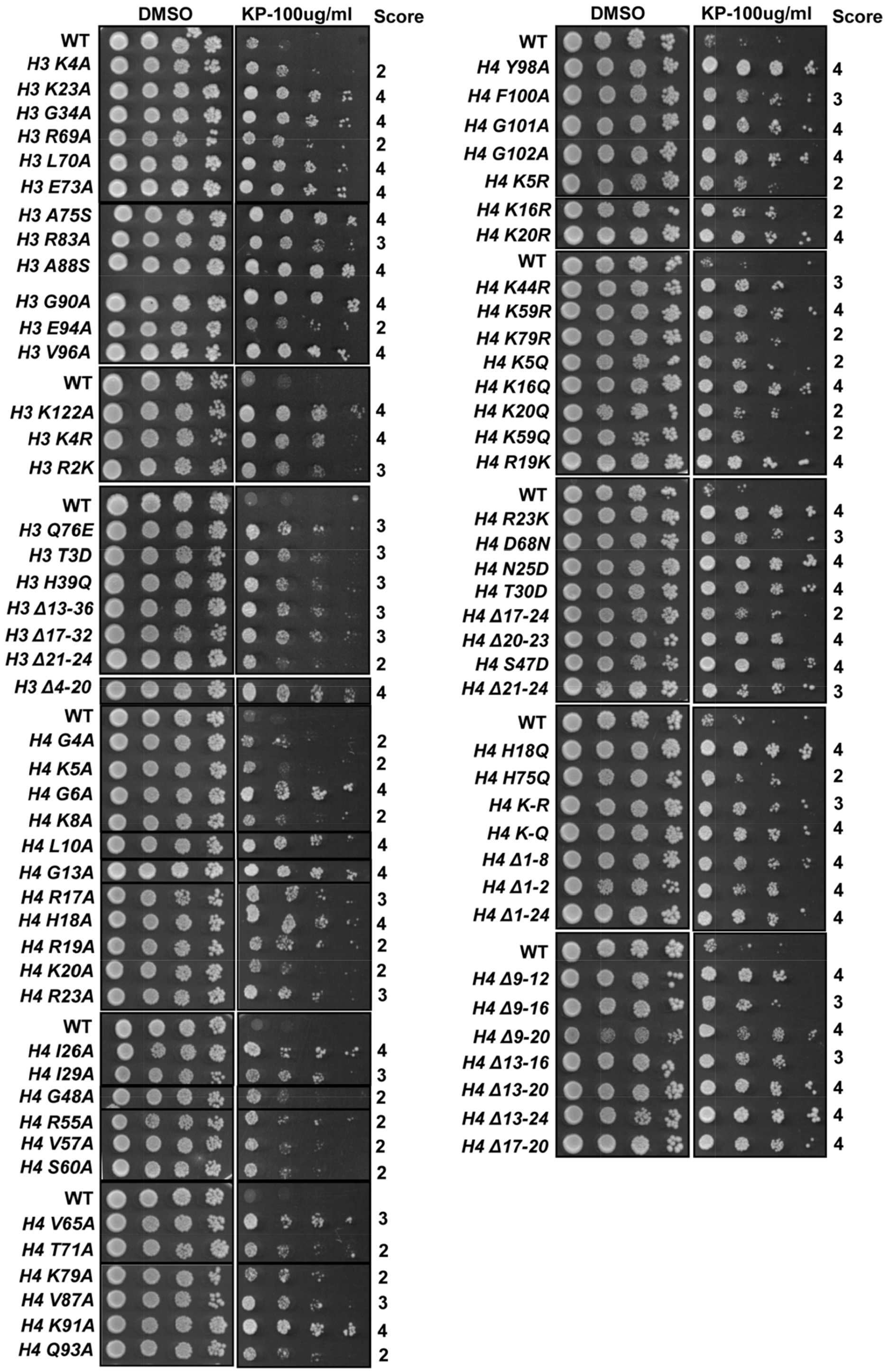

Supplementary Figure 16: Validation and scoring of KP1019 resistant mutants obtained in high-throughput screening. Ten-fold serial dilutions of wild-type (H3 and H4; WT), mutants of histone H3 and H4 were spotted onto SC-agar plates supplemented without or with KP1019 (100μg/ml). The plates were incubated at 30°C and imaged after 48h. The scores for each of KP1019 resistant mutants was given (2 to 4) based on their growth fitness as described in the 'Methods' section.

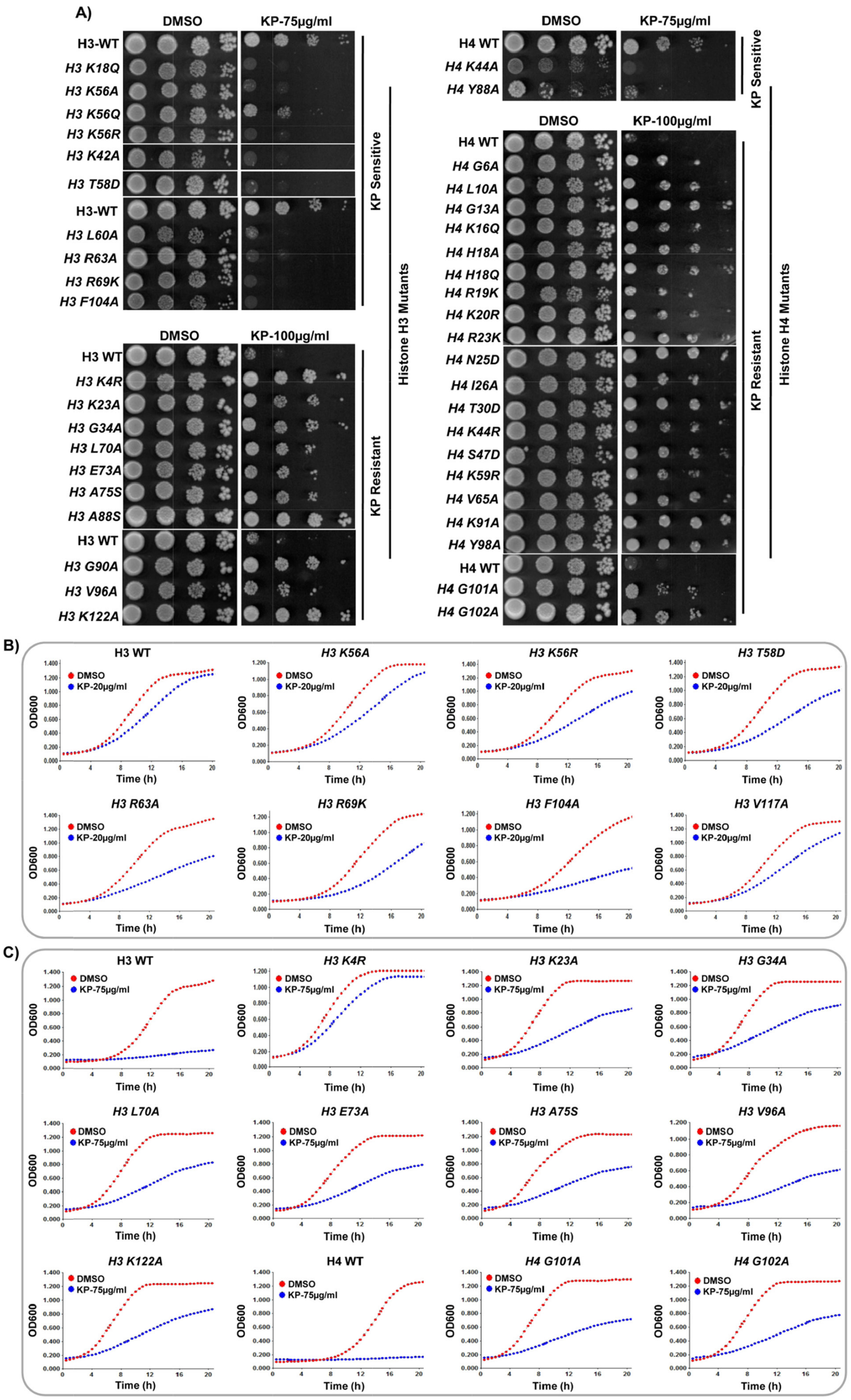

Supplementary Figure 17: Validation of top-scored KP1019 sensitive and resistant mutants. **(A)** Mutations in histone H3/H4 leads to the modulation of KP1019 induced toxicity. Ten-fold serial dilutions of wild-type (H3 and H4; WT), few top-scored KP1019 sensitive, and resistant mutants of histone H3/H4 were spotted onto SC-agar plates supplemented without or with indicated dose of KP1019. The plates were incubated at 30°C and imaged after 48h. **(B & C)** Validation of KP1019 sensitive and resistant histone H3/H4 mutants by growth curve analysis. The exponentially growing histone H3/H4 wild-type and selected (top-scored) KP1019 sensitive (B) and resistant (C) mutant cells were treated with either DMSO solvent (control) or indicated doses of KP1019. The growth was monitored in terms of absorbance (OD600) for 20h using a plate reader.

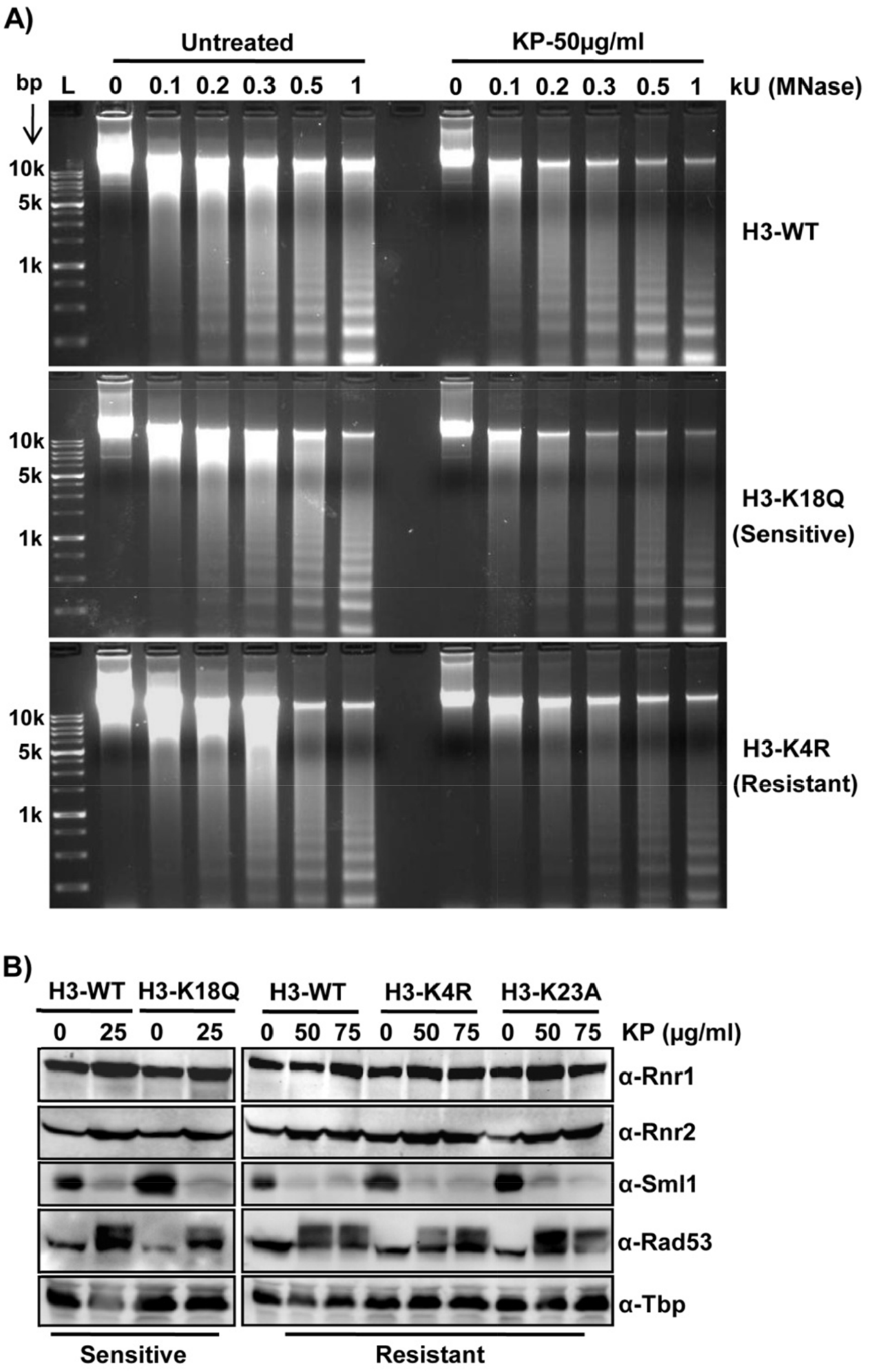

Supplementary Figure 18: KP1019 activates the DNA damage repair response in histone mutants without altering global chromatin accessibility. **(A)** The treatment of sensitive and resistant mutants with KP1019 does not alter global chromatin structure. The exponentially growing wild-type (H3), *H3-K18Q*, and *H3-K4R* mutant cells were left untreated (DMSO only) or treated with KP1019 (50μg/ml) for 6h. The nuclei were isolated and digested with increasing concentration (kilo Units/ml) of MNase, genomic DNA were isolated and analyzed on 1.2% agarose gel. 'L' denotes the DNA ladder while 'bp' denotes the 'base pair'. **(B)** KP1019 treatment leads to Rad53 phosphorylation in both the sensitive and resistant mutants. The exponentially growing wild-type (H3), KP1019 sensitive (*H3-K18Q*), and resistant (*H3-K4R* and *H3-K23A*) mutant cells were left untreated (DMSO only) or treated with the indicated dose of KP1019 for 3h and then harvested. The whole-cell protein extracts were subjected to western blot analysis using the indicated antibodies. Anti-Tbp signals were used to check the protein loading.

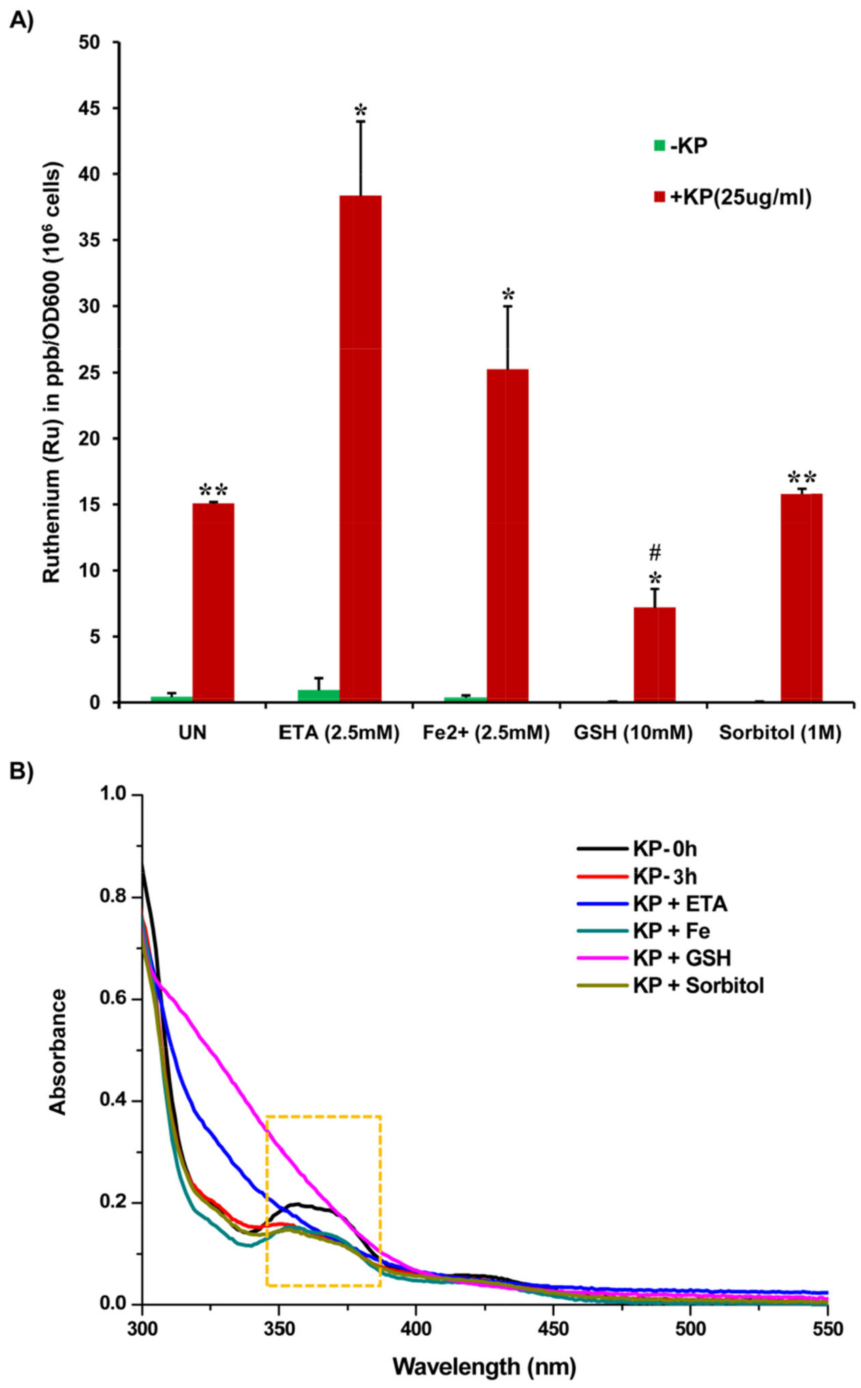

Supplementary Figure 19: Effect of ETA, Fe^{2+}, GSH, and Sorbitol supplementation on the uptake of KP1019. **(A)** The uptake of KP1019 by yeast cells is reduced by the presence of GSH. The exponentially growing wild-type (BY4743) yeast cells were treated with either DMSO (-KP1019) or KP1019 (25µg/ml), ETA (2.5mM), Fe^{2+} (2.5mM), GSH (10mM), and Sorbitol (1M) in alone and combination for 3h. Accumulation of KP1019 in terms of ruthenium (Ru) was measured by ICP-MS. The values are Mean ± SEM (n=2) and represented as parts per billion (ppb) of Ru/OD600 (10^6 cells). $^{*}P<0.05$, $^{**}P<0.005$ (compared to untreated-UN; –KP1019) and $^{\#}P<0.05$ (compared to only KP1019 treated; +KP1019) were considered significant (Student's t-test). **(B)** Absorption spectrum of KP1019 was altered in the presence of ETA and GSH. The exponentially growing wild-type (BY4743) yeast cells were treated with either DMSO (-KP1019) or KP1019 (25µg/ml), ETA (2.5mM), Fe^{2+} (2.5mM), GSH (10mM), and Sorbitol (1M) in alone and combination for 3h at 30°C. The absorption spectra of residual KP1019 present in SC media after 3h treatment were obtained by UV-Vis spectrophotometer. The UV-Vis absorption spectrum of KP1019 (characteristic peak is indicated by a dotted box) in SC media at 0h was served as control.

Supplementary Table 1: List of yeast strains used in this study

See Supplementary File 1

Supplementary Table 2: Complete list of differentially expressed genes (fold-change>1.5, moderated t-test BH-FDR corrected $p<0.05$) in *Saccharomyces cerevisiae* (W1588-4C) cells upon KP1019 (50µg/ml) treatment for 3h relative to untreated (control) cells

See Supplementary File 2

Supplementary Table 3: Complete list of significantly ($p<0.1$) over-represented MIPS (The Munich Information Center for Protein Sequences) functional categories in the dataset of KP1019 induced transcriptome (>1.5 fold) obtained by FunSpec bioinformatics tool

See Supplementary File 3

Supplementary Table 4: Complete list of significantly (p<0.1) over-represented MIPS (The Munich Information Center for Protein Sequences) functional categories in the dataset of KP1019 repressed transcriptome (>1.5 fold) obtained by FunSpec bioinformatics tool

See Supplementary File 4

Supplementary Table 5: Functional phenotypes associated with the KP1019 sensitive histone H3/H4 library mutants

See Supplementary File 5

Supplementary Table 6: Functional phenotypes associated with the KP1019 resistant histone H3/H4 library mutants

See Supplementary File 6
