# Supplementary material for: A systematic assessment of chemical, genetic, and epigenetic factors influencing the activity of anticancer drug KP1019 (FFC14A)
Source: Oncotarget. 2017 Sep 30;8(58):98426–54. doi: 10.18632/oncotarget.21416 (PMC5716741; doi:10.18632/oncotarget.21416)
Supplement: Supplementary file 2 [file oncotarget-08-98426-s002.docx]

**Supplementary Table 1: List of yeast strains used in this study**

| **S.No.** | **Strain Name** | **Genotype** | **Mutation** | **Source/Lab (Reference)** |
| --- | --- | --- | --- | --- |
| 1 | W1588-4C | MATa ade2-1 can1-100 his3-11,15 leu2-3, 112 trp1-1 ura3-1 RAD5+ | Wild-type (WT) | Andrei Chabes [[9](#_ENREF_9)] |
| 2 | YDS203-6b | W303 (MATa ade2-1 his3-11,15 leu2-3,112 ura3-1 trp1-1 can1-100; rap1::LEU2; Ycp50-RAP1 | RAP1-WT | Joseph C Reese [[10](#_ENREF_10)] |
| 3 | YJR1076 | W303; rap1::LEU2; rap1-6 (rap1 ∆43-279 [HIS3/CEN]) | *rap1ΔN* | Joseph C Reese |
| 4 | YJR1073 | W303; rap1::LEU2; rap1-8 (rap1 1-716 [HIS3/CEN]) | *rap1ΔC* | Joseph C Reese |
| 5 | BY4741 | MATa his3Δ1 leu2Δ0 met15Δ0 ura3Δ0 | Wild-type (WT) | Axel Mogk [[11](#_ENREF_11)] |
| 6 | GSHY583 | MATα his3Δ1 leu2Δ0 lys2Δ0 ura3Δ0; ss-dsRed-HDEL::natNT1 | *ss-dsRed-HDEL* | Axel Mogk |
| 7 | SCY62 | MATa his3-11,15 leu2-3,112 ura3-1 trp1-1 can1-100 ADE2 | Wild-type (WT) | Sten Stymne [[12](#_ENREF_12)] |
| 8 | H1246 | MATα are1∆::HIS3 are2∆::LEU2 dga1∆::KanMX4 lro1∆::TRP1 ADE2 | *are1∆are2∆ dga1∆lro1∆* | Sten Stymne |
| 9 | BY4743 | MATa/α his3Δ1/his3Δ1 leu2Δ0/leu2Δ0 LYS2/lys2Δ0 met15Δ0/MET15 ura3Δ0/ura3Δ0 | Wild-type (WT) | Yeast Knockout Collection-Open Biosystems(YKO-OB) |
| 10 | *acc1Δ/Δ* | Isogenic to BY4743; acc1Δ::KanMX4 | *acc1Δ* | YKO-OB |
| 11 | *aft1Δ/Δ* | Isogenic to BY4743; aft1Δ::KanMX4 | *aft1Δ* | YKO-OB |
| 12 | *apq12Δ/Δ* | Isogenic to BY4743; apq12Δ::KanMX4 | *apq12Δ* | YKO-OB |
| 13 | *are1Δ/Δ* | Isogenic to BY4743; are1Δ::KanMX4 | *are1Δ* | YKO-OB |
| 14 | *are2Δ/Δ* | Isogenic to BY4743; are2Δ::KanMX4 | *are2Δ* | YKO-OB |
| 15 | *arv1Δ/Δ* | Isogenic to BY4743; arv1Δ::KanMX4 | *arv1Δ* | YKO-OB |
| 16 | *atg26Δ/Δ* | Isogenic to BY4743; atg26Δ::KanMX4 | *atg26Δ* | YKO-OB |
| 17 | *atx1Δ/Δ* | Isogenic to BY4743; atx1Δ::KanMX4 | *atx1Δ* | YKO-OB |
| 18 | *aur1Δ/Δ* | Isogenic to BY4743; aur1Δ::KanMX4 | *aur1Δ* | YKO-OB |
| 19 | *ayr1Δ/Δ* | Isogenic to BY4743; ayr1Δ::KanMX4 | *ayr1Δ* | YKO-OB |
| 20 | *bck1Δ/Δ* | Isogenic to BY4743; bck1Δ::KanMX4 | *bck1Δ* | YKO-OB |
| 21 | *bni1Δ/Δ* | Isogenic to BY4743; bni1Δ::KanMX4 | *bni1Δ* | YKO-OB |
| 22 | *bro1Δ/Δ* | Isogenic to BY4743; bro1Δ::KanMX4 | *bro1Δ* | YKO-OB |
| 23 | *brr6Δ/Δ* | Isogenic to BY4743; brr6Δ::KanMX4 | *brr6Δ* | YKO-OB |
| 24 | *bxi1Δ/Δ* | Isogenic to BY4743; bxi1Δ::KanMX4 | *bxi1Δ* | YKO-OB |
| 25 | *cam1Δ/Δ* | Isogenic to BY4743; cam1Δ::KanMX4 | *cam1Δ* | YKO-OB |
| 26 | *ccc1Δ/Δ* | Isogenic to BY4743; ccc1Δ::KanMX4 | *ccc1Δ* | YKO-OB |
| 27 | *ccc2Δ/Δ* | Isogenic to BY4743; ccc2Δ::KanMX4 | *ccc2Δ* | YKO-OB |
| 28 | *cch1Δ/Δ* | Isogenic to BY4743; cch1Δ::KanMX4 | *cch1Δ* | YKO-OB |
| 29 | *cds1Δ/Δ* | Isogenic to BY4743; cds1Δ::KanMX4 | *cds1Δ* | YKO-OB |
| 30 | *chc1Δ/Δ* | Isogenic to BY4743; chc1Δ::KanMX4 | *chc1Δ* | YKO-OB |
| 31 | *cho1Δ/Δ* | Isogenic to BY4743; cho1Δ::KanMX4 | *cho1Δ* | YKO-OB |
| 32 | *cho2Δ/Δ* | Isogenic to BY4743; cho2Δ::KanMX4 | *cho2Δ* | YKO-OB |
| 33 | *cki1Δ/Δ* | Isogenic to BY4743; cki1Δ::KanMX4 | *cki1Δ* | YKO-OB |
| 34 | *cnb1Δ/Δ* | Isogenic to BY4743; cnb1Δ::KanMX4 | *cnb1Δ* | YKO-OB |
| 35 | *cpt1Δ/Δ* | Isogenic to BY4743; cpt1Δ::KanMX4 | *cpt1Δ* | YKO-OB |
| 36 | *crg1Δ/Δ* | Isogenic to BY4743; crg1Δ::KanMX4 | *crg1Δ* | YKO-OB |
| 37 | *crz1Δ/Δ* | Isogenic to BY4743; crz1Δ::KanMX4 | *crz1Δ* | YKO-OB |
| 38 | *csc1Δ/Δ* | Isogenic to BY4743; csc1Δ::KanMX4 | *csc1Δ* | YKO-OB |
| 39 | *cse2Δ/Δ* | Isogenic to BY4743; cse2Δ::KanMX4 | *cse2Δ* | YKO-OB |
| 40 | *csg1Δ/Δ* | Isogenic to BY4743; csg1Δ::KanMX4 | *csg1Δ* | YKO-OB |
| 41 | *csg2Δ/Δ* | Isogenic to BY4743; csg2Δ::KanMX4 | *csg2Δ* | YKO-OB |
| 42 | *csh1Δ/Δ* | Isogenic to BY4743; csh1Δ::KanMX4 | *csh1Δ* | YKO-OB |
| 43 | *cup5Δ/Δ* | Isogenic to BY4743; cup5Δ::KanMX4 | *cup5Δ* | YKO-OB |
| 44 | *cyb5Δ/Δ* | Isogenic to BY4743; cyb5Δ::KanMX4 | *cyb5Δ* | YKO-OB |
| 45 | *cyc8Δ/Δ* | Isogenic to BY4743; cyc8Δ::KanMX4 | *cyc8Δ* | YKO-OB |
| 46 | *cys3Δ/Δ* | Isogenic to BY4743; cys3Δ::KanMX4 | *cys3Δ* | YKO-OB |
| 47 | *cys4Δ/Δ* | Isogenic to BY4743; cys4Δ::KanMX4 | *cys4Δ* | YKO-OB |
| 48 | *dep1Δ/Δ* | Isogenic to BY4743; dep1Δ::KanMX4 | *dep1Δ* | YKO-OB |
| 49 | *dga1Δ/Δ* | Isogenic to BY4743; dga1Δ::KanMX4 | *dga1Δ* | YKO-OB |
| 50 | *dgk1Δ/Δ* | Isogenic to BY4743; dgk1Δ::KanMX4 | *dgk1Δ* | YKO-OB |
| 51 | *dhh1Δ/Δ* | Isogenic to BY4743; dhh1Δ::KanMX4 | *dhh1Δ* | YKO-OB |
| 52 | *dpp1Δ/Δ* | Isogenic to BY4743; dpp1Δ::KanMX4 | *dpp1Δ* | YKO-OB |
| 53 | *ecm22Δ/Δ* | Isogenic to BY4743; ecm22Δ::KanMX4 | *ecm22Δ* | YKO-OB |
| 54 | *ecm27Δ/Δ* | Isogenic to BY4743; ecm27Δ::KanMX4 | *ecm27Δ* | YKO-OB |
| 55 | *ecm7Δ/Δ* | Isogenic to BY4743; ecm7Δ::KanMX4 | *ecm7Δ* | YKO-OB |
| 56 | *ect1Δ/Δ* | Isogenic to BY4743; ect1Δ::KanMX4 | *ect1Δ* | YKO-OB |
| 57 | *ede1Δ/Δ* | Isogenic to BY4743; ede1Δ::KanMX4 | *ede1Δ* | YKO-OB |
| 58 | *eeb1Δ/Δ* | Isogenic to BY4743; eeb1Δ::KanMX4 | *eeb1Δ* | YKO-OB |
| 59 | *eki1Δ/Δ* | Isogenic to BY4743; eki1Δ::KanMX4 | *eki1Δ* | YKO-OB |
| 60 | *elo1Δ/Δ* | Isogenic to BY4743; elo1Δ::KanMX4 | *elo1Δ* | YKO-OB |
| 61 | *elo2Δ/Δ* | Isogenic to BY4743; elo2Δ::KanMX4 | *elo2Δ* | YKO-OB |
| 62 | *elo3Δ/Δ* | Isogenic to BY4743; elo3Δ::KanMX4 | *elo3Δ* | YKO-OB |
| 63 | *ept1Δ/Δ* | Isogenic to BY4743; ept1Δ::KanMX4 | *ept1Δ* | YKO-OB |
| 64 | *erg1Δ/Δ* | Isogenic to BY4743; erg1Δ::KanMX4 | *erg1Δ* | YKO-OB |
| 65 | *erg10Δ/Δ* | Isogenic to BY4743; erg10Δ::KanMX4 | *erg10Δ* | YKO-OB |
| 66 | *erg11Δ/Δ* | Isogenic to BY4743; erg11Δ::KanMX4 | *erg11Δ* | YKO-OB |
| 67 | *erg12Δ/Δ* | Isogenic to BY4743; erg12Δ::KanMX4 | *erg12Δ* | YKO-OB |
| 68 | *erg13Δ/Δ* | Isogenic to BY4743; erg13Δ::KanMX4 | *erg13Δ* | YKO-OB |
| 69 | *erg2Δ/Δ* | Isogenic to BY4743; erg2Δ::KanMX4 | *erg2Δ* | YKO-OB |
| 70 | *erg20Δ/Δ* | Isogenic to BY4743; erg20Δ::KanMX4 | *erg20Δ* | YKO-OB |
| 71 | *erg25Δ/Δ* | Isogenic to BY4743; erg25Δ::KanMX4 | *erg25Δ* | YKO-OB |
| 72 | *erg26Δ/Δ* | Isogenic to BY4743; erg26Δ::KanMX4 | *erg26Δ* | YKO-OB |
| 73 | *erg27Δ/Δ* | Isogenic to BY4743; erg27Δ::KanMX4 | *erg27Δ* | YKO-OB |
| 74 | *erg3Δ/Δ* | Isogenic to BY4743; erg3Δ::KanMX4 | *erg3Δ* | YKO-OB |
| 75 | *erg4Δ/Δ* | Isogenic to BY4743; erg4Δ::KanMX4 | *erg4Δ* | YKO-OB |
| 76 | *erg5Δ/Δ* | Isogenic to BY4743; erg5Δ::KanMX4 | *erg5Δ* | YKO-OB |
| 77 | *erg6Δ/Δ* | Isogenic to BY4743; erg6Δ::KanMX4 | *erg6Δ* | YKO-OB |
| 78 | *erg7Δ/Δ* | Isogenic to BY4743; erg7Δ::KanMX4 | *erg7Δ* | YKO-OB |
| 79 | *erg8Δ/Δ* | Isogenic to BY4743; erg8Δ::KanMX4 | *erg8Δ* | YKO-OB |
| 80 | *erg9Δ/Δ* | Isogenic to BY4743; erg9Δ::KanMX4 | *erg9Δ* | YKO-OB |
| 81 | *faa1Δ/Δ* | Isogenic to BY4743; faa1Δ::KanMX4 | *faa1Δ* | YKO-OB |
| 82 | *faa2Δ/Δ* | Isogenic to BY4743; faa2Δ::KanMX4 | *faa2Δ* | YKO-OB |
| 83 | *faa3Δ/Δ* | Isogenic to BY4743; faa3Δ::KanMX4 | *faa3Δ* | YKO-OB |
| 84 | *faa4Δ/Δ* | Isogenic to BY4743; faa4Δ::KanMX4 | *faa4Δ* | YKO-OB |
| 85 | *fab1Δ/Δ* | Isogenic to BY4743; fab1Δ::KanMX4 | *fab1Δ* | YKO-OB |
| 86 | *fas1Δ/Δ* | Isogenic to BY4743; fas1Δ::KanMX4 | *fas1Δ* | YKO-OB |
| 87 | *fas2Δ/Δ* | Isogenic to BY4743; fas2Δ::KanMX4 | *fas2Δ* | YKO-OB |
| 88 | *fat1Δ/Δ* | Isogenic to BY4743; fat1Δ::KanMX4 | *fat1Δ* | YKO-OB |
| 89 | *fat2Δ/Δ* | Isogenic to BY4743; fat2Δ::KanMX4 | *fat2Δ* | YKO-OB |
| 90 | *fes1Δ/Δ* | Isogenic to BY4743; fes1Δ::KanMX4 | *fes1Δ* | YKO-OB |
| 91 | *fet3Δ/Δ* | Isogenic to BY4743; fet3Δ::KanMX4 | *fet3Δ* | YKO-OB |
| 92 | *fet5Δ/Δ* | Isogenic to BY4743; fet5Δ::KanMX4 | *fet5Δ* | YKO-OB |
| 93 | *fig4Δ/Δ* | Isogenic to BY4743; fig4Δ::KanMX4 | *fig4Δ* | YKO-OB |
| 94 | *flc2Δ/Δ* | Isogenic to BY4743; flc2Δ::KanMX4 | *flc2Δ* | YKO-OB |
| 95 | *fox2Δ/Δ* | Isogenic to BY4743; fox2Δ::KanMX4 | *fox2Δ* | YKO-OB |
| 96 | *fpr1Δ/Δ* | Isogenic to BY4743; fox2Δ::KanMX4 | *fpr1Δ* | YKO-OB |
| 97 | *fre1Δ/Δ* | Isogenic to BY4743; fre1Δ::KanMX4 | *fre1Δ* | YKO-OB |
| 98 | *fre2Δ/Δ* | Isogenic to BY4743; fre2Δ::KanMX4 | *fre2Δ* | YKO-OB |
| 99 | *fre3Δ/Δ* | Isogenic to BY4743; fre3Δ::KanMX4 | *fre3Δ* | YKO-OB |
| 100 | *fre4Δ/Δ* | Isogenic to BY4743; fre4Δ::KanMX4 | *fre4Δ* | YKO-OB |
| 101 | *fre5Δ/Δ* | Isogenic to BY4743; fre5Δ::KanMX4 | *fre5Δ* | YKO-OB |
| 102 | *fre6Δ/Δ* | Isogenic to BY4743; fre6Δ::KanMX4 | *fre6Δ* | YKO-OB |
| 103 | *fre7Δ/Δ* | Isogenic to BY4743; fre7Δ::KanMX4 | *fre7Δ* | YKO-OB |
| 104 | *fth1Δ/Δ* | Isogenic to BY4743; fth1Δ::KanMX4 | *fth1Δ* | YKO-OB |
| 105 | *ftr1Δ/Δ* | Isogenic to BY4743; ftr1Δ::KanMX4 | *ftr1Δ* | YKO-OB |
| 106 | *gap1Δ/Δ* | Isogenic to BY4743; gap1Δ::KanMX4 | *gap1Δ* | YKO-OB |
| 107 | *gas1Δ/Δ* | Isogenic to BY4743; gas1Δ::KanMX4 | *gas1Δ* | YKO-OB |
| 108 | *gat1Δ/Δ* | Isogenic to BY4743; gat1Δ::KanMX4 | *gat1Δ* | YKO-OB |
| 109 | *gdt1Δ/Δ* | Isogenic to BY4743; gdt1Δ::KanMX4 | *gdt1Δ* | YKO-OB |
| 110 | *gem1Δ/Δ* | Isogenic to BY4743; gem1Δ::KanMX4 | *gem1Δ* | YKO-OB |
| 111 | *gln3Δ/Δ* | Isogenic to BY4743; gln3Δ::KanMX4 | *gln3Δ* | YKO-OB |
| 112 | *gmc1Δ/Δ* | Isogenic to BY4743; gmc1Δ::KanMX4 | *gmc1Δ* | YKO-OB |
| 113 | *gsc2Δ/Δ* | Isogenic to BY4743; gsc2Δ::KanMX4 | *gsc2Δ* | YKO-OB |
| 114 | *gup1Δ/Δ* | Isogenic to BY4743; gup1Δ::KanMX4 | *gup1Δ* | YKO-OB |
| 115 | *hac1Δ/Δ* | Isogenic to BY4743; hac1Δ::KanMX4 | *hac1Δ* | YKO-OB |
| 116 | *ino2Δ/Δ* | Isogenic to BY4743; ino2Δ::KanMX4 | *ino2Δ* | YKO-OB |
| 117 | *ino4Δ/Δ* | Isogenic to BY4743; ino4Δ::KanMX4 | *ino4Δ* | YKO-OB |
| 118 | *inp51Δ/Δ* | Isogenic to BY4743; inp51Δ::KanMX4 | *inp51Δ* | YKO-OB |
| 119 | *inp52Δ/Δ* | Isogenic to BY4743; inp52Δ::KanMX4 | *inp52Δ* | YKO-OB |
| 120 | *inp53Δ/Δ* | Isogenic to BY4743; inp53Δ::KanMX4 | *inp53Δ* | YKO-OB |
| 121 | *inp54Δ/Δ* | Isogenic to BY4743; inp54Δ::KanMX4 | *inp54Δ* | YKO-OB |
| 122 | *ipt1Δ/Δ* | Isogenic to BY4743; ipt1Δ::KanMX4 | *ipt1Δ* | YKO-OB |
| 123 | *ira2Δ/Δ* | Isogenic to BY4743; ira2Δ::KanMX4 | *ira2Δ* | YKO-OB |
| 124 | *ire1Δ/Δ* | Isogenic to BY4743; ire1Δ::KanMX4 | *ire1Δ* | YKO-OB |
| 125 | *isc1Δ/Δ* | Isogenic to BY4743; isc1Δ::KanMX4 | *isc1Δ* | YKO-OB |
| 126 | *lac1Δ/Δ* | Isogenic to BY4743; lac1Δ::KanMX4 | *lac1Δ* | YKO-OB |
| 127 | *lag1Δ/Δ* | Isogenic to BY4743; lag1Δ::KanMX4 | *lag1Δ* | YKO-OB |
| 128 | *lcb2Δ/Δ* | Isogenic to BY4743; lcb2Δ::KanMX4 | *lcb2Δ* | YKO-OB |
| 129 | *lcb3Δ/Δ* | Isogenic to BY4743; lcb3Δ::KanMX4 | *lcb3Δ* | YKO-OB |
| 130 | *lcb4Δ/Δ* | Isogenic to BY4743; lcb4Δ::KanMX4 | *lcb4Δ* | YKO-OB |
| 131 | *lcb5Δ/Δ* | Isogenic to BY4743; lcb5Δ::KanMX4 | *lcb5Δ* | YKO-OB |
| 132 | *ldh1Δ/Δ* | Isogenic to BY4743; ldh1Δ::KanMX4 | *ldh1Δ* | YKO-OB |
| 133 | *lem3Δ/Δ* | Isogenic to BY4743; lem3Δ::KanMX4 | *lem3Δ* | YKO-OB |
| 134 | *lpl1Δ/Δ* | Isogenic to BY4743; lpl1Δ::KanMX4 | *lpl1Δ* | YKO-OB |
| 135 | *lpp1Δ/Δ* | Isogenic to BY4743; lpp1Δ::KanMX4 | *lpp1Δ* | YKO-OB |
| 136 | *lro1Δ/Δ* | Isogenic to BY4743; lro1Δ::KanMX4 | *lro1Δ* | YKO-OB |
| 137 | *lsb6Δ/Δ* | Isogenic to BY4743; lsb6Δ::KanMX4 | *lsb6Δ* | YKO-OB |
| 138 | *mck1Δ/Δ* | Isogenic to BY4743; mck1Δ::KanMX4 | *mck1Δ* | YKO-OB |
| 139 | *mcp1Δ/Δ* | Isogenic to BY4743; mcp1Δ::KanMX4 | *mcp1Δ* | YKO-OB |
| 140 | *mcp2Δ/Δ* | Isogenic to BY4743; mcp2Δ::KanMX4 | *mcp2Δ* | YKO-OB |
| 141 | *mdm34Δ/Δ* | Isogenic to BY4743; mdm34Δ::KanMX4 | *mdm34Δ* | YKO-OB |
| 142 | *met6Δ/Δ* | Isogenic to BY4743; met6Δ::KanMX4 | *met6Δ* | YKO-OB |
| 143 | *mga2Δ/Δ* | Isogenic to BY4743; mga2Δ::KanMX4 | *mga2Δ* | YKO-OB |
| 144 | *mid1Δ/Δ* | Isogenic to BY4743; mid1Δ::KanMX4 | *mid1Δ* | YKO-OB |
| 145 | *mid2Δ/Δ* | Isogenic to BY4743; mid2Δ::KanMX4 | *mid2Δ* | YKO-OB |
| 146 | *mkk1Δ/Δ* | Isogenic to BY4743; mkk1Δ::KanMX4 | *mkk1Δ* | YKO-OB |
| 147 | *mkk2Δ/Δ* | Isogenic to BY4743; mkk2Δ::KanMX4 | *mkk2Δ* | YKO-OB |
| 148 | *mmm1Δ/Δ* | Isogenic to BY4743; mmm1Δ::KanMX4 | *mmm1Δ* | YKO-OB |
| 149 | *mot3Δ/Δ* | Isogenic to BY4743; mot3Δ::KanMX4 | *mot3Δ* | YKO-OB |
| 150 | *mrs3Δ/Δ* | Isogenic to BY4743; mrs3Δ::KanMX4 | *mrs3Δ* | YKO-OB |
| 151 | *mrs4Δ/Δ* | Isogenic to BY4743; mrs4Δ::KanMX4 | *mrs4Δ* | YKO-OB |
| 152 | *mrx5Δ/Δ* | Isogenic to BY4743; mrx5Δ::KanMX4 | *mrx5Δ* | YKO-OB |
| 153 | *mss4Δ/Δ* | Isogenic to BY4743; mss4Δ::KanMX4 | *mss4Δ* | YKO-OB |
| 154 | *ncr1Δ/Δ* | Isogenic to BY4743; ncr1Δ::KanMX4 | *ncr1Δ* | YKO-OB |
| 155 | *nde1Δ/Δ* | Isogenic to BY4743; nde1Δ::KanMX4 | *nde1Δ* | YKO-OB |
| 156 | *npr1Δ/Δ* | Isogenic to BY4743; npr1Δ::KanMX4 | *npr1Δ* | YKO-OB |
| 157 | *nte1Δ/Δ* | Isogenic to BY4743; nte1Δ::KanMX4 | *nte1Δ* | YKO-OB |
| 158 | *nvj2Δ/Δ* | Isogenic to BY4743; nvj2Δ::KanMX4 | *nvj2Δ* | YKO-OB |
| 159 | *oaf1Δ/Δ* | Isogenic to BY4743; oaf1Δ::KanMX4 | *oaf1Δ* | YKO-OB |
| 160 | *ole1Δ/Δ* | Isogenic to BY4743; ole1Δ::KanMX4 | *ole1Δ* | YKO-OB |
| 161 | *opi3Δ/Δ* | Isogenic to BY4743; opi3Δ::KanMX4 | *opi3Δ* | YKO-OB |
| 162 | *opi9Δ/Δ* | Isogenic to BY4743; opi9Δ::KanMX4 | *opi9Δ* | YKO-OB |
| 163 | *opt2Δ/Δ* | Isogenic to BY4743; opt2Δ::KanMX4 | *opt2Δ* | YKO-OB |
| 164 | *pah1Δ/Δ* | Isogenic to BY4743; pah1Δ::KanMX4 | *pah1Δ* | YKO-OB |
| 165 | *pcl1Δ/Δ* | Isogenic to BY4743; pcl1Δ::KanMX4 | *pcl1Δ* | YKO-OB |
| 166 | *pct1Δ/Δ* | Isogenic to BY4743; pct1Δ::KanMX4 | *pct1Δ* | YKO-OB |
| 167 | *pdr16Δ/Δ* | Isogenic to BY4743; pdr16Δ::KanMX4 | *pdr16Δ* | YKO-OB |
| 168 | *pef1Δ/Δ* | Isogenic to BY4743; pef1Δ::KanMX4 | *pef1Δ* | YKO-OB |
| 169 | *per1Δ/Δ* | Isogenic to BY4743; per1Δ::KanMX4 | *per1Δ* | YKO-OB |
| 170 | *pet10Δ/Δ* | Isogenic to BY4743; pet10Δ::KanMX4 | *pet10Δ* | YKO-OB |
| 171 | *pgs1Δ/Δ* | Isogenic to BY4743; pgs1Δ::KanMX4 | *pgs1Δ* | YKO-OB |
| 172 | *pho80Δ/Δ* | Isogenic to BY4743; pho80Δ::KanMX4 | *pho80Δ* | YKO-OB |
| 173 | *pho90Δ/Δ* | Isogenic to BY4743; pho90Δ::KanMX4 | *pho90Δ* | YKO-OB |
| 174 | *phs1Δ/Δ* | Isogenic to BY4743; phs1Δ::KanMX4 | *phs1Δ* | YKO-OB |
| 175 | *pik1Δ/Δ* | Isogenic to BY4743; pik1Δ::KanMX4 | *pik1Δ* | YKO-OB |
| 176 | *pip2Δ/Δ* | Isogenic to BY4743; pip2Δ::KanMX4 | *pip2Δ* | YKO-OB |
| 177 | *pis1Δ/Δ* | Isogenic to BY4743; pis1Δ::KanMX4 | *pis1Δ* | YKO-OB |
| 178 | *plb1Δ/Δ* | Isogenic to BY4743; plb1Δ::KanMX4 | *plb1Δ* | YKO-OB |
| 179 | *plb2Δ/Δ* | Isogenic to BY4743; plb2Δ::KanMX4 | *plb2Δ* | YKO-OB |
| 180 | *plb3Δ/Δ* | Isogenic to BY4743; plb3Δ::KanMX4 | *plb3Δ* | YKO-OB |
| 181 | *pmc1Δ/Δ* | Isogenic to BY4743; pmc1Δ::KanMX4 | *pmc1Δ* | YKO-OB |
| 182 | *pmr1Δ/Δ* | Isogenic to BY4743; pmr1Δ::KanMX4 | *pmr1Δ* | YKO-OB |
| 183 | *pox1Δ/Δ* | Isogenic to BY4743; pox1Δ::KanMX4 | *pox1Δ* | YKO-OB |
| 184 | *ppa2Δ/Δ* | Isogenic to BY4743; ppa2Δ::KanMX4 | *ppa2Δ* | YKO-OB |
| 185 | *pre9Δ/Δ* | Isogenic to BY4743; pre9Δ::KanMX4 | *pre9Δ* | YKO-OB |
| 186 | *prm5Δ/Δ* | Isogenic to BY4743; prm5Δ::KanMX4 | *prm5Δ* | YKO-OB |
| 187 | *psd2Δ/Δ* | Isogenic to BY4743; psd2Δ::KanMX4 | *psd2Δ* | YKO-OB |
| 188 | *ptp2Δ/Δ* | Isogenic to BY4743; ptp2Δ::KanMX4 | *ptp2Δ* | YKO-OB |
| 189 | *rch1Δ/Δ* | Isogenic to BY4743; rch1Δ::KanMX4 | *rch1Δ* | YKO-OB |
| 190 | *rcn1Δ/Δ* | Isogenic to BY4743; rcn1Δ::KanMX4 | *rcn1Δ* | YKO-OB |
| 191 | *ree1Δ/Δ* | Isogenic to BY4743; ree1Δ::KanMX4 | *ree1Δ* | YKO-OB |
| 192 | *rlm1Δ/Δ* | Isogenic to BY4743; rlm1Δ::KanMX4 | *rlm1Δ* | YKO-OB |
| 193 | *rom2Δ/Δ* | Isogenic to BY4743; rom2Δ::KanMX4 | *rom2Δ* | YKO-OB |
| 194 | *rps27bΔ/Δ* | Isogenic to BY4743; rps27bΔ::KanMX4 | *rps27bΔ* | YKO-OB |
| 195 | *rvs167Δ/Δ* | Isogenic to BY4743; rvs167Δ::KanMX4 | *rvs167Δ* | YKO-OB |
| 196 | *sac1Δ/Δ* | Isogenic to BY4743; sac1Δ::KanMX4 | *sac1Δ* | YKO-OB |
| 197 | *sah1Δ/Δ* | Isogenic to BY4743; sah1Δ::KanMX4 | *sah1Δ* | YKO-OB |
| 198 | *sam1Δ/Δ* | Isogenic to BY4743; sam1Δ::KanMX4 | *sam1Δ* | YKO-OB |
| 199 | *sam2Δ/Δ* | Isogenic to BY4743; sam2Δ::KanMX4 | *sam2Δ* | YKO-OB |
| 200 | *sch9Δ/Δ* | Isogenic to BY4743; sch9Δ::KanMX4 | *sch9Δ* | YKO-OB |
| 201 | *scs7Δ/Δ* | Isogenic to BY4743; scs7Δ::KanMX4 | *scs7Δ* | YKO-OB |
| 202 | *sct1Δ/Δ* | Isogenic to BY4743; sct1Δ::KanMX4 | *sct1Δ* | YKO-OB |
| 203 | *sfp1Δ/Δ* | Isogenic to BY4743; sfp1Δ::KanMX4 | *sfp1Δ* | YKO-OB |
| 204 | *sit4Δ/Δ* | Isogenic to BY4743; sit4Δ::KanMX4 | *sit4Δ* | YKO-OB |
| 205 | *skn7Δ/Δ* | Isogenic to BY4743; skn7Δ::KanMX4 | *skn7Δ* | YKO-OB |
| 206 | *sky1Δ/Δ* | Isogenic to BY4743; sky1Δ::KanMX4 | *sky1Δ* | YKO-OB |
| 207 | *slc1Δ/Δ* | Isogenic to BY4743; slc1Δ::KanMX4 | *slc1Δ* | YKO-OB |
| 208 | *slc4Δ/Δ* | Isogenic to BY4743; slc4Δ::KanMX4 | *slc4Δ* | YKO-OB |
| 209 | *slt2Δ/Δ* | Isogenic to BY4743; slt2Δ::KanMX4 | *slt2Δ* | YKO-OB |
| 210 | *smf1Δ/Δ* | Isogenic to BY4743; smf1Δ::KanMX4 | *smf1Δ* | YKO-OB |
| 211 | *smf2Δ/Δ* | Isogenic to BY4743; smf2Δ::KanMX4 | *smf2Δ* | YKO-OB |
| 212 | *smf3Δ/Δ* | Isogenic to BY4743; smf3Δ::KanMX4 | *smf3Δ* | YKO-OB |
| 213 | *snf1Δ/Δ* | Isogenic to BY4743; snf1Δ::KanMX4 | *snf1Δ* | YKO-OB |
| 214 | *snf2Δ/Δ* | Isogenic to BY4743; snf2Δ::KanMX4 | *snf2Δ* | YKO-OB |
| 215 | *spe2Δ/Δ* | Isogenic to BY4743; spe2Δ::KanMX4 | *spe2Δ* | YKO-OB |
| 216 | *spf1Δ/Δ* | Isogenic to BY4743; spf1Δ::KanMX4 | *spf1Δ* | YKO-OB |
| 217 | *spo14Δ/Δ* | Isogenic to BY4743; spo14Δ::KanMX4 | *spo14Δ* | YKO-OB |
| 218 | *spo22Δ/Δ* | Isogenic to BY4743; spo22Δ::KanMX4 | *spo22Δ* | YKO-OB |
| 219 | *spt21Δ/Δ* | Isogenic to BY4743; spt21Δ::KanMX4 | *spt21Δ* | YKO-OB |
| 220 | *spt23Δ/Δ* | Isogenic to BY4743; spt23Δ::KanMX4 | *spt23Δ* | YKO-OB |
| 221 | *srl3Δ/Δ* | Isogenic to BY4743; srl3Δ::KanMX4 | *srl3Δ* | YKO-OB |
| 222 | *stt4Δ/Δ* | Isogenic to BY4743; stt4Δ::KanMX4 | *stt4Δ* | YKO-OB |
| 223 | *sur2Δ/Δ* | Isogenic to BY4743; sur2Δ::KanMX4 | *sur2Δ* | YKO-OB |
| 224 | *swi4Δ/Δ* | Isogenic to BY4743; swi4Δ::KanMX4 | *swi4Δ* | YKO-OB |
| 225 | *tap42Δ/TAP42* | Isogenic to BY4743; TAP42/tap42Δ::KanMX4 | *tap42Δ/TAP42* | YKO-OB |
| 226 | *tcb1Δ/Δ* | Isogenic to BY4743; tcb1Δ::KanMX4 | *tcb1Δ* | YKO-OB |
| 227 | *tcb2Δ/Δ* | Isogenic to BY4743; tcb2Δ::KanMX4 | *tcb2Δ* | YKO-OB |
| 228 | *tco89Δ/Δ* | Isogenic to BY4743; tco89Δ::KanMX4 | *tco89Δ* | YKO-OB |
| 229 | *tgl1Δ/Δ* | Isogenic to BY4743; tgl1Δ::KanMX4 | *tgl1Δ* | YKO-OB |
| 230 | *tgl3Δ/Δ* | Isogenic to BY4743; tgl3Δ::KanMX4 | *tgl3Δ* | YKO-OB |
| 231 | *tgl4Δ/Δ* | Isogenic to BY4743; tgl4Δ::KanMX4 | *tgl4Δ* | YKO-OB |
| 232 | *tgl5Δ/Δ* | Isogenic to BY4743; tgl5Δ::KanMX4 | *tgl5Δ* | YKO-OB |
| 233 | *tif4631Δ/Δ* | Isogenic to BY4743; tif4631Δ::KanMX4 | *tif4631Δ* | YKO-OB |
| 234 | *tlg2Δ/Δ* | Isogenic to BY4743; tlg2Δ::KanMX4 | *tlg2Δ* | YKO-OB |
| 235 | *tor1Δ/Δ* | Isogenic to BY4743; tor1Δ::KanMX4 | *tor1Δ* | YKO-OB |
| 236 | *tor2Δ/TOR2* | Isogenic to BY4743; TOR2/tor2Δ::KanMX4 | *tor2Δ/TOR2* | YKO-OB |
| 237 | *tpk1Δ/Δ* | Isogenic to BY4743; tpk1Δ::KanMX4 | *tpk1Δ* | YKO-OB |
| 238 | *tsc10Δ/Δ* | Isogenic to BY4743; tsc10Δ::KanMX4 | *tsc10Δ* | YKO-OB |
| 239 | *tsc13Δ/Δ* | Isogenic to BY4743; tsc13Δ::KanMX4 | *tsc13Δ* | YKO-OB |
| 240 | *tsc3Δ/Δ* | Isogenic to BY4743; tsc3Δ::KanMX4 | *tsc3Δ* | YKO-OB |
| 241 | *tup1Δ/Δ* | Isogenic to BY4743; tup1Δ::KanMX4 | *tup1Δ* | YKO-OB |
| 242 | *upc2Δ/Δ* | Isogenic to BY4743; upc2Δ::KanMX4 | *upc2Δ* | YKO-OB |
| 243 | *vcx1Δ/Δ* | Isogenic to BY4743; vcx1Δ::KanMX4 | *vcx1Δ* | YKO-OB |
| 244 | *vnx1Δ/Δ* | Isogenic to BY4743; vnx1Δ::KanMX4 | *vnx1Δ* | YKO-OB |
| 245 | *vps16Δ/Δ* | Isogenic to BY4743; vps16Δ::KanMX4 | *vps16Δ* | YKO-OB |
| 246 | *vps20Δ/Δ* | Isogenic to BY4743; vps20Δ::KanMX4 | *vps20Δ* | YKO-OB |
| 247 | *vps34Δ/Δ* | Isogenic to BY4743; vps34Δ::KanMX4 | *vps34Δ* | YKO-OB |
| 248 | *vps36Δ/Δ* | Isogenic to BY4743; vps36Δ::KanMX4 | *vps36Δ* | YKO-OB |
| 249 | *vps38Δ/Δ* | Isogenic to BY4743; vps38Δ::KanMX4 | *vps38Δ* | YKO-OB |
| 250 | *vps53Δ/Δ* | Isogenic to BY4743; vps53Δ::KanMX4 | *vps53Δ* | YKO-OB |
| 251 | *vps8Δ/Δ* | Isogenic to BY4743; vps8Δ::KanMX4 | *vps8Δ* | YKO-OB |
| 252 | *vrp1Δ/Δ* | Isogenic to BY4743; vrp1Δ::KanMX4 | *vrp1Δ* | YKO-OB |
| 253 | *wsc1Δ/Δ* | Isogenic to BY4743; wsc1Δ::KanMX4 | *wsc1Δ* | YKO-OB |
| 254 | *wsc2Δ/Δ* | Isogenic to BY4743; wsc2Δ::KanMX4 | *wsc2Δ* | YKO-OB |
| 255 | *wsc3Δ/Δ* | Isogenic to BY4743; wsc3Δ::KanMX4 | *wsc3Δ* | YKO-OB |
| 256 | *ybl094cΔ/Δ* | Isogenic to BY4743; ybl094cΔ::KanMX4 | *ybl094cΔ* | YKO-OB |
| 257 | *ydc1Δ/Δ* | Isogenic to BY4743; ydc1Δ::KanMX4 | *ydc1Δ* | YKO-OB |
| 258 | *ydl109cΔ/Δ* | Isogenic to BY4743; ydl109cΔ::KanMX4 | *ydl109cΔ* | YKO-OB |
| 259 | *ydr114cΔ/Δ* | Isogenic to BY4743; ydr114cΔ::KanMX4 | *ydr114cΔ* | YKO-OB |
| 260 | *yeh1Δ/Δ* | Isogenic to BY4743; yeh1Δ::KanMX4 | *yeh1Δ* | YKO-OB |
| 261 | *yeh2Δ/Δ* | Isogenic to BY4743; yeh2Δ::KanMX4 | *yeh2Δ* | YKO-OB |
| 262 | *yer034wΔ/Δ* | Isogenic to BY4743; yer034wΔ::KanMX4 | *yer034wΔ* | YKO-OB |
| 263 | *yim1Δ/Δ* | Isogenic to BY4743; yim1Δ::KanMX4 | *yim1Δ* | YKO-OB |
| 264 | *yjl175wΔ/Δ* | Isogenic to BY4743; yjl175wΔ::KanMX4 | *yjl175wΔ* | YKO-OB |
| 265 | *yjr124cΔ/Δ* | Isogenic to BY4743; yjr124cΔ::KanMX4 | *yjr124cΔ* | YKO-OB |
| 266 | *ykl091cΔ/Δ* | Isogenic to BY4743; ykl091cΔ::KanMX4 | *ykl091cΔ* | YKO-OB |
| 267 | *yml002wΔ/Δ* | Isogenic to BY4743; yml002wΔ::KanMX4 | *yml002wΔ* | YKO-OB |
| 268 | *yml089cΔ/Δ* | Isogenic to BY4743; yml089cΔ::KanMX4 | *yml089cΔ* | YKO-OB |
| 269 | *ymr210wΔ/Δ* | Isogenic to BY4743; ymr210wΔ::KanMX4 | *ymr210wΔ* | YKO-OB |
| 270 | *ypc1Δ/Δ* | Isogenic to BY4743; ypc1Δ::KanMX4 | *ypc1Δ* | YKO-OB |
| 271 | *ypr147cΔ/Δ* | Isogenic to BY4743; ypr147cΔ::KanMX4 | *ypr147cΔ* | YKO-OB |
| 272 | *yta7Δ/Δ* | Isogenic to BY4743; yta7Δ::KanMX4 | *yta7Δ* | YKO-OB |
| 273 | *zap1Δ/Δ* | Isogenic to BY4743; zap1Δ::KanMX4 | *zap1Δ* | YKO-OB |
