# Supplementary material for: A systematic assessment of chemical, genetic, and epigenetic factors influencing the activity of anticancer drug KP1019 (FFC14A)
Source: Oncotarget. 2017 Sep 30;8(58):98426–54. doi: 10.18632/oncotarget.21416 (PMC5716741; doi:10.18632/oncotarget.21416)
Supplement: Supplementary file 3 [file oncotarget-08-98426-s003.docx]

**SupplementaryTable 2: Complete list of differentially expressed genes (fold-change>1.5, moderated t-test BH-FDR corrected p<0.05) in** ***Saccharomyces cerevisiae* (W1588-4C) cells upon KP1019 (50µg/ml) treatment for 3h relative to untreated (control) cells**

| **Probe Set ID** | **Gene Symbol** | **Systematic Name** | **Gene Title** | | **Fold Change (FC)** | **Log FC** | **Regulation** |
| --- | --- | --- | --- | --- | --- | --- | --- |
| 1780044_at | RNR3 | YIL066C | Minor isoform of the large subunit of ribonucleotide-diphosphate reductase; the RNR complex catalyzes rate-limiting step in dNTP synthesis, regulated by DNA replication and DNA damage checkpoint pathways via localization of small subunits | | 25.79112 | 4.688803 | up |
| 1776837_at | HUG1 | YML058W-A | Protein involved in the Mec1p-mediated checkpoint pathway that responds to DNA damage or replication arrest, transcription is induced by DNA damage | | 21.05465 | 4.396067 | up |
| 1770298_at | RPS9A | YPL081W | Protein component of the small (40S) ribosomal subunit; nearly identical to Rps9Bp and has similarity to E. coli S4 and rat S9 ribosomal proteins | | 16.45595 | 4.040537 | up |
| 1778919_at | RNR1 | YER070W | Major isoform of the large subunit of ribonucleotide-diphosphate reductase; the RNR complex catalyzes rate-limiting step in dNTP synthesis, regulated by DNA replication and DNA damage checkpoint pathways via localization of small subunits | | 15.73432 | 3.975843 | up |
| 1769443_at | PLM2 | YDR501W | Forkhead Associated domain containing protein and putative transcription factor found associated with chromatin; target of SBF transcription factor; induced in response to DNA damaging agents and deletion of telomerase; similar to TOS4 | | 12.862 | 3.685043 | up |
| 1771055_at | RPL18B | YNL301C | Protein component of the large (60S) ribosomal subunit, identical to Rpl18Ap and has similarity to rat L18 ribosomal protein | | 12.76416 | 3.674027 | up |
| 1774888_at |  | YJL045W |  | | 12.68435 | 3.664977 | up |
| 1773427_at | RPS22B | YLR367W | Protein component of the small (40S) ribosomal subunit; nearly identical to Rps22Ap and has similarity to E. coli S8 and rat S15a ribosomal proteins | | 6.411696 | 2.680706 | up |
| 1771497_at | RPS24B | YIL069C | Protein component of the small (40S) ribosomal subunit; identical to Rps24Ap and has similarity to rat S24 ribosomal protein | | 5.903783 | 2.56164 | up |
| 1775717_at |  | YLR413W |  | | 5.5807 | 2.480446 | up |
| 1770957_at |  | YOR378W |  | | 5.572671 | 2.478369 | up |
| 1774236_at | PLB2 | YMR006C | Phospholipase B (lysophospholipase) involved in phospholipid metabolism; displays transacylase activity in vitro; overproduction confers resistance to lysophosphatidylcholine | | 5.528277 | 2.46683 | up |
| 1777679_at | DIN7 | YDR263C | Mitochondrial nuclease functioning in DNA repair and replication, modulates the stability of the mitochondrial genome, induced by exposure to mutagens, also induced during meiosis at a time nearly coincident with commitment to recombination | | 5.116274 | 2.355094 | up |
| 1772935_at | NBL1 | YHR199C-A | Subunit of the conserved chromosomal passenger complex (CPC; Ipl1p-Sli15p-Bir1p-Nbl1p), which regulates mitotic chromosome segregation; not required for the kinase activity of the complex; mediates the interaction of Sli15p and Bir1p | | 5.004421 | 2.323203 | up |
| 1772742_at | RPS0B | YLR048W | Protein component of the small (40S) ribosomal subunit, nearly identical to Rps0Ap; required for maturation of 18S rRNA along with Rps0Ap; deletion of either RPS0 gene reduces growth rate, deletion of both genes is lethal | | 4.983883 | 2.31727 | up |
| 1778491_at | TRZ1 | YKR079C | tRNA 3'-end processing endonuclease tRNase Z; also localized to mitochondria and interacts genetically with Rex2 exonuclease; homolog of the human candidate prostate cancer susceptibility gene ELAC2 | | 4.790262 | 2.260105 | up |
| 1776735_at | RPL34A | YER056C-A | Protein component of the large (60S) ribosomal subunit, nearly identical to Rpl34Bp and has similarity to rat L34 ribosomal protein | | 4.739683 | 2.244791 | up |
| 1775186_at | RPS14B | YJL191W | Ribosomal protein 59 of the small subunit, required for ribosome assembly and 20S pre-rRNA processing; mutations confer cryptopleurine resistance; nearly identical to Rps14Ap and similar to E. coli S11 and rat S14 ribosomal proteins | | 4.670645 | 2.223622 | up |
| 1770864_at | RAD53 | YPL153C | Protein kinase, required for cell-cycle arrest in response to DNA damage; activated by trans autophosphorylation when interacting with hyperphosphorylated Rad9p; also interacts with ARS1 and plays a role in initiation of DNA replication | | 4.583663 | 2.196501 | up |
| 1770501_at | RPS8B | YER102W | Protein component of the small (40S) ribosomal subunit; identical to Rps8Ap and has similarity to rat S8 ribosomal protein | | 4.38477 | 2.132501 | up |
| 1772734_at | RPL9B | YNL067W | Protein component of the large (60S) ribosomal subunit, nearly identical to Rpl9Ap and has similarity to E. coli L6 and rat L9 ribosomal proteins | | 4.332665 | 2.115255 | up |
| 1770945_at | NMD4 | YLR363C | Protein interacting with Nam7p, may be involved in the nonsense-mediated mRNA decay pathway | | 4.311368 | 2.108146 | up |
| 1776278_at | RPS7B | YNL096C | Protein component of the small (40S) ribosomal subunit, nearly identical to Rps7Ap; interacts with Kti11p; deletion causes hypersensitivity to zymocin; has similarity to rat S7 and Xenopus S8 ribosomal proteins | | 4.308279 | 2.107112 | up |
| 1774976_at | NOC4 | YPR144C | Nucleolar protein, forms a complex with Nop14p that mediates maturation and nuclear export of 40S ribosomal subunits | | 4.306588 | 2.106545 | up |
| 1776467_at | RPL8A | YHL033C | Ribosomal protein L4 of the large (60S) ribosomal subunit, nearly identical to Rpl8Bp and has similarity to rat L7a ribosomal protein; mutation results in decreased amounts of free 60S subunits | | 4.289987 | 2.100973 | up |
| 1778296_at | CSI2 | YOL007C | Protein of unknown function; green fluorescent protein (GFP)- fusion protein localizes to the mother side of the bud neck and the vacuole; YOL007C is not an essential gene | | 4.255703 | 2.089397 | up |
| 1772366_at | RPS1B | YML063W | Ribosomal protein 10 (rp10) of the small (40S) subunit; nearly identical to Rps1Ap and has similarity to rat S3a ribosomal protein | | 4.255361 | 2.089282 | up |
| 1778542_at | TCA17 | YEL048C | Subunit of TRAPPII, a multimeric GEF involved in intra-Golgi and endosome-to-Golgi transport; promotes association of TRAPPII-specific subunits with the core complex; sedlin related; human Sedlin mutations cause SEDT, a skeletal disorder | | 4.248317 | 2.086891 | up |
| 1778943_at | RIM2 | YBR192W | Mitochondrial pyrimidine nucleotide transporter; imports pyrimidine nucleoside triphosphates and exports pyrimidine nucleoside monophosphates; member of the mitochondrial carrier family | | 4.197502 | 2.069531 | up |
| 1772016_at | MAM33 | YIL070C | Acidic protein of the mitochondrial matrix involved in oxidative phosphorylation; related to the human complement receptor gC1q-R | | 4.167094 | 2.059042 | up |
| 1774174_at | RPL6B | YLR448W | Protein component of the large (60S) ribosomal subunit, has similarity to Rpl6Ap and to rat L6 ribosomal protein; binds to 5.8S rRNA | | 4.127621 | 2.045311 | up |
| 1778277_at | SST2 | YLR452C | GTPase-activating protein for Gpa1p, regulates desensitization to alpha factor pheromone; also required to prevent receptor-independent signaling of the mating pathway; member of the RGS (regulator of G-protein signaling) family | | 4.103083 | 2.036708 | up |
| 1777104_at | RPL6A | YML073C | N-terminally acetylated protein component of the large (60S) ribosomal subunit, has similarity to Rpl6Bp and to rat L6 ribosomal protein; binds to 5.8S rRNA | | 4.064201 | 2.022972 | up |
| 1770683_at | SLD5 | YDR489W | Subunit of the GINS complex (Sld5p, Psf1p, Psf2p, Psf3p), which is localized to DNA replication origins and implicated in assembly of the DNA replication machinery | | 4.03447 | 2.012379 | up |
| 1776719_at | ATP10 | YLR393W | Mitochondrial inner membrane protein required for assembly of the F0 sector of mitochondrial F1F0 ATP synthase | | 3.989293 | 1.996133 | up |
| 1770437_at |  | YPR015C |  | | 3.980464 | 1.992937 | up |
| 1776235_at | POL2 | YNL262W | Catalytic subunit of DNA polymerase (II) epsilon, a chromosomal DNA replication polymerase that exhibits processivity and proofreading exonuclease activity; also involved in DNA synthesis during DNA repair; interacts extensively with Mrc1p | | 3.945153 | 1.980081 | up |
| 1776844_at | PRM7 | YDL038C | Pheromone-regulated protein, predicted to have one transmembrane segment; promoter contains Gcn4p binding elements | | 3.854064 | 1.946381 | up |
| 1778886_at | RPS15 | YOL040C | Protein component of the small (40S) ribosomal subunit; has similarity to E. coli S19 and rat S15 ribosomal proteins | | 3.840614 | 1.941337 | up |
| 1773865_at | RRN5 | YLR141W | Protein involved in transcription of rDNA by RNA polymerase I; transcription factor, member of UAF (upstream activation factor) family along with Rrn9p and Rrn10p | | 3.834225 | 1.938935 | up |
| 1779803_x_at | AAD3 | YCR107W | Putative aryl-alcohol dehydrogenase; similar to P. chrysosporium aryl-alcohol dehydrogenase; mutational analysis has not yet revealed a physiological role | | 3.815132 | 1.931733 | up |
| 1775158_at |  | YDL241W |  | | 3.768957 | 1.914166 | up |
| 1772218_at | RIM9 | YMR063W | Protein of unknown function, involved in the proteolytic activation of Rim101p in response to alkaline pH; has similarity to A. nidulans PalI; putative membrane protein | | 3.709573 | 1.891253 | up |
| 1779681_at | DIT1 | YDR403W | Sporulation-specific enzyme required for spore wall maturation, involved in the production of a soluble LL-dityrosine-containing precursor of the spore wall; transcripts accumulate at the time of prospore enclosure | | 3.68679 | 1.882365 | up |
| 1778735_at |  | YGR035C |  | | 3.684864 | 1.881611 | up |
| 1771711_at | SEN34 | YAR008W | Subunit of the tRNA splicing endonuclease, which is composed of Sen2p, Sen15p, Sen34p, and Sen54p; Sen34p contains the active site for tRNA 3' splice site cleavage and has similarity to Sen2p and to Archaeal tRNA splicing endonuclease | | 3.667888 | 1.87495 | up |
| 1772341_at | UGO1 | YDR470C | Outer membrane component of the mitochondrial fusion machinery; binds directly to Fzo1p and Mgm1p and thus links these two GTPases during mitochondrial fusion; involved in fusion of both the outer and inner membranes; facilitates dimerization of Fzo1p during fusion; import into the outer membrane is mediated by Tom70p and Mim1p; has similarity to carrier proteins but is not likely to function as a transmembrane transporter | | 3.62509 | 1.858017 | up |
| 1774776_at | PPT2 | YPL148C | Phosphopantetheine:protein transferase (PPTase), activates mitochondrial acyl carrier protein (Acp1p) by phosphopantetheinylation | | 3.611351 | 1.852539 | up |
| 1776324_at | MEI4 | YER044C-A | Meiosis-specific protein involved in double-strand break formation during meiotic recombination; required for chromosome synapsis and production of viable spores | | 3.59732 | 1.846923 | up |
| 1779582_at | LST7 | YGR057C | Protein possibly involved in a post-Golgi secretory pathway; required for the transport of nitrogen-regulated amino acid permease Gap1p from the Golgi to the cell surface | | 3.589273 | 1.843692 | up |
| 1770115_at | SPH1 | YLR313C | Protein involved in shmoo formation and bipolar bud site selection; homologous to Spa2p, localizes to sites of polarized growth in a cell cycle dependent- and Spa2p-dependent manner, interacts with MAPKKs Mkk1p, Mkk2p, and Ste7p | | 3.582385 | 1.84092 | up |
| 1777923_at |  | YDR042C |  | | 3.570132 | 1.835977 | up |
| 1772631_at | RFU1 | YLR073C | Protein that inhibits Doa4p deubiquitinating activity; contributes to ubiquitin homeostasis by regulating the conversion of free ubiquitin chains to ubiquitin monomers by Doa4p; GFP-fusion protein localizes to endosomes | | 3.541137 | 1.824213 | up |
| 1777823_s_at |  | YLR312C-B |  | | 3.50957 | 1.811294 | up |
| 1776104_at | RPS28B | YLR264W | Protein component of the small (40S) ribosomal subunit; nearly identical to Rps28Ap and has similarity to rat S28 ribosomal protein | | 3.503463 | 1.808782 | up |
| 1772208_at | RPS11B | YBR048W | Protein component of the small (40S) ribosomal subunit; identical to Rps11Ap and has similarity to E. coli S17 and rat S11 ribosomal proteins | | 3.502564 | 1.808412 | up |
| 1779097_at | RKI1 | YOR095C | Ribose-5-phosphate ketol-isomerase, catalyzes the interconversion of ribose 5-phosphate and ribulose 5-phosphate in the pentose phosphate pathway; participates in pyridoxine biosynthesis | | 3.493475 | 1.804663 | up |
| 1775510_at | TOS2 | YGR221C | Protein involved in localization of Cdc24p to the site of bud growth; may act as a membrane anchor; localizes to the bud neck and bud tip; potentially phosphorylated by Cdc28p | | 3.491754 | 1.803952 | up |
| 1776546_at | BSC1 | YDL037C | Protein of unconfirmed function, similar to cell surface flocculin Muc1p; ORF exhibits genomic organization compatible with a translational readthrough-dependent mode of expression | | 3.456373 | 1.789259 | up |
| 1779787_at | RPL21A | YBR191W | Protein component of the large (60S) ribosomal subunit, nearly identical to Rpl21Bp and has similarity to rat L21 ribosomal protein | | 3.372692 | 1.753901 | up |
| 1769503_at | RPS17B | YDR447C | Ribosomal protein 51 (rp51) of the small (40s) subunit; nearly identical to Rps17Ap and has similarity to rat S17 ribosomal protein | | 3.35525 | 1.74642 | up |
| 1779532_at | RPL17B | YJL177W | Protein component of the large (60S) ribosomal subunit, nearly identical to Rpl17Ap and has similarity to E. coli L22 and rat L17 ribosomal proteins | | 3.326315 | 1.733925 | up |
| 1776516_at | RPL13A | YDL082W | Protein component of the large (60S) ribosomal subunit, nearly identical to Rpl13Bp; not essential for viability; has similarity to rat L13 ribosomal protein | | 3.324217 | 1.733015 | up |
| 1771832_at | RPL12A | YEL054C | Protein component of the large (60S) ribosomal subunit, nearly identical to Rpl12Bp; rpl12a rpl12b double mutant exhibits slow growth and slow translation; has similarity to E. coli L11 and rat L12 ribosomal proteins | | 3.272574 | 1.710426 | up |
| 1772857_at | RPS8A | YBL072C | Protein component of the small (40S) ribosomal subunit; identical to Rps8Bp and has similarity to rat S8 ribosomal protein | | 3.239933 | 1.695964 | up |
| 1778133_s_at | RPL7A | YGL076C | Protein component of the large (60S) ribosomal subunit, nearly identical to Rpl7Bp and has similarity to E. coli L30 and rat L7 ribosomal proteins; contains a conserved C-terminal Nucleic acid Binding Domain (NDB2)///Protein component of the large (60S) ribosomal subunit, nearly identical to Rpl7Ap and has similarity to E. coli L30 and rat L7 ribosomal proteins; contains a conserved C-terminal Nucleic acid Binding Domain (NDB2) | | 3.17293 | 1.665816 | up |
| 1779526_at |  | YOR114W |  | | 3.170144 | 1.664548 | up |
| 1776757_at | CIN2 | YPL241C | GTPase-activating protein (GAP) for Cin4p; tubulin folding factor C involved in beta-tubulin (Tub2p) folding; mutants display increased chromosome loss and benomyl sensitivity; deletion complemented by human GAP, retinitis pigmentosa 2 | | 3.136438 | 1.649127 | up |
| 1779256_at | FUI1 | YBL042C | High affinity uridine permease, localizes to the plasma membrane; also mediates low but significant transport of the cytotoxic nucleoside analog 5-fluorouridine; not involved in uracil transport | | 3.134739 | 1.648346 | up |
| 1770882_at | RPL13B | YMR142C | Protein component of the large (60S) ribosomal subunit, nearly identical to Rpl13Ap; not essential for viability; has similarity to rat L13 ribosomal protein | | 3.037406 | 1.60284 | up |
| 1771373_at | SPS100 | YHR139C | Protein required for spore wall maturation; expressed during sporulation; may be a component of the spore wall; expression also induced in cells treated with the mycotoxin patulin | | 3.036302 | 1.602315 | up |
| 1779227_at | CDC6 | YJL194W | Essential ATP-binding protein required for DNA replication, component of the pre-replicative complex (pre-RC) which requires ORC to associate with chromatin and is in turn required for Mcm2-7p DNA association; homologous to S. pombe Cdc18p | | 3.015727 | 1.592506 | up |
| 1776619_at | URB2 | YJR041C | Nucleolar protein required for normal metabolism of the rRNA primary transcript, proposed to be involved in ribosome biogenesis | | 3.00366 | 1.586722 | up |
| 1775832_at | RPS20 | YHL015W | Protein component of the small (40S) ribosomal subunit; overproduction suppresses mutations affecting RNA polymerase III-dependent transcription; has similarity to E. coli S10 and rat S20 ribosomal proteins | | 2.993102 | 1.581641 | up |
| 1780116_at | RPS21B | YJL136C | Protein component of the small (40S) ribosomal subunit; nearly identical to Rps21Ap and has similarity to rat S21 ribosomal protein | | 2.992134 | 1.581175 | up |
| 1770413_at | RPL24A | YGL031C | Ribosomal protein L30 of the large (60S) ribosomal subunit, nearly identical to Rpl24Bp and has similarity to rat L24 ribosomal protein; not essential for translation but may be required for normal translation rate | | 2.991114 | 1.580683 | up |
| 1769638_at |  | YNL095C |  | | 2.98801 | 1.579185 | up |
| 1779816_at | RPL33B | YOR234C | Ribosomal protein L37 of the large (60S) ribosomal subunit, nearly identical to Rpl33Ap and has similarity to rat L35a; rpl33b null mutant exhibits normal growth while rpl33a rpl33b double null mutant is inviable | | 2.98627 | 1.578345 | up |
| 1775837_at | SSN8 | YNL025C | Cyclin-like component of the RNA polymerase II holoenzyme, involved in phosphorylation of the RNA polymerase II C-terminal domain; involved in glucose repression and telomere maintenance | | 2.981919 | 1.576241 | up |
| 1776927_at | RPL9A | YGL147C | Protein component of the large (60S) ribosomal subunit, nearly identical to Rpl9Bp and has similarity to E. coli L6 and rat L9 ribosomal proteins | | 2.969966 | 1.570447 | up |
| 1773510_at | RPL27B | YDR471W | Protein component of the large (60S) ribosomal subunit, nearly identical to Rpl27Ap and has similarity to rat L27 ribosomal protein | | 2.968824 | 1.569891 | up |
| 1770979_at | FLX1 | YIL134W | Protein required for transport of flavin adenine dinucleotide (FAD), a synthesis product of riboflavin, across the mitochondrial membrane | | 2.936827 | 1.554258 | up |
| 1776025_at | RPS9B | YBR189W | Protein component of the small (40S) ribosomal subunit; nearly identical to Rps9Ap and has similarity to E. coli S4 and rat S9 ribosomal proteins | | 2.922519 | 1.547213 | up |
| 1772477_at |  | YJR124C |  | | 2.898147 | 1.535131 | up |
| 1777982_at | RPS7A | YOR096W | Protein component of the small (40S) ribosomal subunit, nearly identical to Rps7Bp; interacts with Kti11p; deletion causes hypersensitivity to zymocin; has similarity to rat S7 and Xenopus S8 ribosomal proteins | | 2.883691 | 1.527916 | up |
| 1772965_at | MSA2 | YKR077W | Putative transcriptional activator, that interacts with G1-specific transcription factor, MBF and G1-specific promoters; ortholog of Msa2p, an MBF and SBF activator that regulates G1-specific transcription and cell cycle initiation | | 2.881818 | 1.526979 | up |
| 1771085_at | RPL18A | YOL120C | Protein component of the large (60S) ribosomal subunit, identical to Rpl18Bp and has similarity to rat L18 ribosomal protein; intron of RPL18A pre-mRNA forms stem-loop structures that are a target for Rnt1p cleavage leading to degradation | | 2.87696 | 1.524545 | up |
| 1777211_at | RFX1 | YLR176C | Major transcriptional repressor of DNA-damage-regulated genes, recruits repressors Tup1p and Cyc8p to their promoters; involved in DNA damage and replication checkpoint pathway; similar to a family of mammalian DNA binding RFX1-4 proteins | | 2.869773 | 1.520937 | up |
| 1772638_at | RPL26A | YLR344W | Protein component of the large (60S) ribosomal subunit, nearly identical to Rpl26Bp and has similarity to E. coli L24 and rat L26 ribosomal proteins; binds to 5.8S rRNA | | 2.860736 | 1.516387 | up |
| 1772468_at | RPL40B | YKR094C | Fusion protein, identical to Rpl40Ap, that is cleaved to yield ubiquitin and a ribosomal protein of the large (60S) ribosomal subunit with similarity to rat L40; ubiquitin may facilitate assembly of the ribosomal protein into ribosomes | | 2.832935 | 1.502297 | up |
| 1776906_at | SMK1 | YPR054W | Middle sporulation-specific mitogen-activated protein kinase (MAPK) required for production of the outer spore wall layers; negatively regulates activity of the glucan synthase subunit Gsc2p | | 2.817426 | 1.494378 | up |
| 1773183_at | MUC1 | YIR019C | GPI-anchored cell surface glycoprotein (flocculin); required for pseudohyphal formation, invasive growth, flocculation, and biofilms; transcriptionally regulated by the MAPK pathway (via Ste12p and Tec1p) and the cAMP pathway (via Flo8p); required for the formation of fibrous interconnections between cells in a colony of a wild S. cerevisiae strain | | 2.813519 | 1.492376 | up |
| 1775626_at | URA7 | YBL039C | Major CTP synthase isozyme (see also URA8), catalyzes the ATP-dependent transfer of the amide nitrogen from glutamine to UTP, forming CTP, the final step in de novo biosynthesis of pyrimidines; involved in phospholipid biosynthesis | | 2.813414 | 1.492322 | up |
| 1771652_at |  | YML096W |  | | 2.809275 | 1.490198 | up |
| 1772893_at | MPS3 | YJL019W | Nuclear envelope protein required for SPB duplication and nuclear fusion; localizes to the SPB half bridge and at telomeres during meiosis; required with Ndj1p and Csm4p for meiotic bouquet formation and telomere-led rapid prophase movement | | 2.786773 | 1.478595 | up |
| 1776772_at | GTO3 | YMR251W | Omega class glutathione transferase; putative cytosolic localization | | 2.756382 | 1.462776 | up |
| 1777817_at | RPS24A | YER074W | Protein component of the small (40S) ribosomal subunit; identical to Rps24Bp and has similarity to rat S24 ribosomal protein | | 2.748138 | 1.458455 | up |
| 1776199_at | RPS23B | YPR132W | Ribosomal protein 28 (rp28) of the small (40S) ribosomal subunit, required for translational accuracy; nearly identical to Rps23Ap and similar to E. coli S12 and rat S23 ribosomal proteins; deletion of both RPS23A and RPS23B is lethal | | 2.738686 | 1.453484 | up |
| 1771443_at | ARG81 | YML099C | Zinc-finger transcription factor of the Zn(2)-Cys(6) binuclear cluster domain type, involved in the regulation of arginine-responsive genes; acts with Arg80p and Arg82p | | 2.724608 | 1.446049 | up |
| 1779680_at |  | YMR310C |  | | 2.72005 | 1.443633 | up |
| 1774459_at | POL12 | YBL035C | B subunit of DNA polymerase alpha-primase complex, required for initiation of DNA replication during mitotic and premeiotic DNA synthesis; also functions in telomere capping and length regulation | | 2.711986 | 1.43935 | up |
| 1770136_at | RPS5 | YJR123W | Protein component of the small (40S) ribosomal subunit, the least basic of the non-acidic ribosomal proteins; phosphorylated in vivo; essential for viability; has similarity to E. coli S7 and rat S5 ribosomal proteins | | 2.704722 | 1.435481 | up |
| 1772870_at | URK1 | YNR012W | Uridine/cytidine kinase, component of the pyrimidine ribonucleotide salvage pathway that converts uridine into UMP and cytidine into CMP; involved in the pyrimidine deoxyribonucleotide salvage pathway, converting deoxycytidine into dCMP | | 2.700902 | 1.433441 | up |
| 1773281_at | RAD54 | YGL163C | DNA-dependent ATPase, stimulates strand exchange by modifying the topology of double-stranded DNA; involved in the recombinational repair of double-strand breaks in DNA during vegetative growth and meiosis; member of the SWI/SNF family | | 2.699924 | 1.432919 | up |
| 1771078_at | FRM2 | YCL026C-A | Type II nitroreductase, using NADH as reductant; mutants are defective in fatty acid mediated repression of genes involved in fatty acid biosynthesis indicative of a role in lipid signaling; involved in the oxidative stress response; transcription induction by cadmium and selenite indicates a possible role in the metal stress response; expression induced in cells treated with the mycotoxin patulin | | 2.699332 | 1.432602 | up |
| 1779033_at | DEG1 | YFL001W | tRNA:pseudouridine synthase, introduces pseudouridines at position 38 or 39 in tRNA, important for maintenance of translation efficiency and normal cell growth, localizes to both the nucleus and cytoplasm; non-essential for viability | | 2.689819 | 1.427509 | up |
| 1776088_at | NRM1 | YNR009W | Transcriptional co-repressor of MBF (MCB binding factor)-regulated gene expression; Nrm1p associates stably with promoters via MBF to repress transcription upon exit from G1 phase | | 2.67323 | 1.418584 | up |
| 1776010_at | RPS22A | YJL190C | Protein component of the small (40S) ribosomal subunit; nearly identical to Rps22Bp and has similarity to E. coli S8 and rat S15a ribosomal proteins | | 2.671943 | 1.417889 | up |
| 1769786_at |  | YOR161C-C |  | | 2.647156 | 1.404443 | up |
| 1777458_at | RPL16B | YNL069C | N-terminally acetylated protein component of the large (60S) ribosomal subunit, binds to 5.8 S rRNA; has similarity to Rpl16Ap, E. coli L13 and rat L13a ribosomal proteins; transcriptionally regulated by Rap1p | | 2.628343 | 1.394154 | up |
| 1775548_at | MSH6 | YDR097C | Protein required for mismatch repair in mitosis and meiosis, forms a complex with Msh2p to repair both single-base & insertion-deletion mispairs; potentially phosphorylated by Cdc28p | | 2.619947 | 1.389538 | up |
| 1770284_at | RPL37A | YLR185W | Protein component of the large (60S) ribosomal subunit, has similarity to Rpl37Bp and to rat L37 ribosomal protein | | 2.608854 | 1.383416 | up |
| 1780240_at | RFA2 | YNL312W | Subunit of heterotrimeric Replication Protein A (RPA), which is a highly conserved single-stranded DNA binding protein involved in DNA replication, repair, and recombination | | 2.588435 | 1.37208 | up |
| 1778903_at |  | YPL068C |  | | 2.554872 | 1.353251 | up |
| 1774042_at | SPC29 | YPL124W | Inner plaque spindle pole body (SPB) component, links the central plaque component Spc42p to the inner plaque component Spc110p; required for SPB duplication | | 2.542552 | 1.346277 | up |
| 1779904_at | YHK8 | YHR048W | Presumed antiporter of the DHA1 family of multidrug resistance transporters; contains 12 predicted transmembrane spans; expression of gene is up-regulated in cells exhibiting reduced susceptibility to azoles | | 2.536484 | 1.34283 | up |
| 1774802_at | RPS11A | YDR025W | Protein component of the small (40S) ribosomal subunit; identical to Rps11Bp and has similarity to E. coli S17 and rat S11 ribosomal proteins | | 2.531486 | 1.339984 | up |
| 1779312_at | MSH3 | YCR092C | Mismatch repair protein, forms dimers with Msh2p that mediate repair of insertion or deletion mutations and removal of nonhomologous DNA ends, contains a PCNA (Pol30p) binding motif required for genome stability | | 2.530814 | 1.339601 | up |
| 1772611_at | FLD1 | YLR404W | Seipin protein involved in lipid droplet morphology, number, and size; proposed to be involved in lipid metabolism; related to the human BSCL2 which is associated with lipodystrophy | | 2.527338 | 1.337618 | up |
| 1771236_at | CAX4 | YGR036C | Dolichyl pyrophosphate (Dol-P-P) phosphatase with a luminally oriented active site in the ER, cleaves the anhydride linkage in Dol-P-P, required for Dol-P-P-linked oligosaccharide intermediate synthesis and protein N-glycosylation | | 2.524264 | 1.335863 | up |
| 1773091_at | RPL2B | YIL018W | Protein component of the large (60S) ribosomal subunit, identical to Rpl2Ap and has similarity to E. coli L2 and rat L8 ribosomal proteins; expression is upregulated at low temperatures | | 2.523676 | 1.335527 | up |
| 1776601_at | TOM5 | YPR133W-A | Component of the TOM (translocase of outer membrane) complex responsible for recognition and initial import of all mitochondrially directed proteins; involved in transfer of precursors from the Tom70p and Tom20p receptors to the Tom40p pore | | 2.518739 | 1.332702 | up |
| 1773420_at |  | YMR147W |  | | 2.515876 | 1.331061 | up |
| 1773960_at | RPS16B | YDL083C | Protein component of the small (40S) ribosomal subunit; identical to Rps16Ap and has similarity to E. coli S9 and rat S16 ribosomal proteins | | 2.514824 | 1.330457 | up |
| 1778481_at | RPL3 | YOR063W | Protein component of the large (60S) ribosomal subunit, has similarity to E. coli L3 and rat L3 ribosomal proteins; involved in the replication and maintenance of killer double stranded RNA virus | | 2.507856 | 1.326455 | up |
| 1777227_at | RPL23A | YBL087C | Protein component of the large (60S) ribosomal subunit, identical to Rpl23Bp and has similarity to E. coli L14 and rat L23 ribosomal proteins | | 2.493799 | 1.318345 | up |
| 1776344_at | RPL14A | YKL006W | N-terminally acetylated protein component of the large (60S) ribosomal subunit, nearly identical to Rpl14Bp and has similarity to rat L14 ribosomal protein; rpl14a csh5 double null mutant exhibits synthetic slow growth | | 2.491825 | 1.317203 | up |
| 1776154_at | ALG14 | YBR070C | Component of UDP-GlcNAc transferase required for the second step of dolichyl-linked oligosaccharide synthesis; anchors the catalytic subunit Alg13p to the ER membrane; similar to bacterial and human glycosyltransferases | | 2.486049 | 1.313855 | up |
| 1769788_at | NSE5 | YML023C | Component of the SMC5-SMC6 complex; this complex plays a key role in the removal of X-shaped DNA structures that arise between sister chromatids during DNA replication and repair | | 2.481839 | 1.31141 | up |
| 1778240_at | MMT1 | YMR177W | Putative metal transporter involved in mitochondrial iron accumulation; closely related to Mmt2p | | 2.477979 | 1.309164 | up |
| 1770710_at | IRC19 | YLL033W | Putative protein of unknown function; YLL033W is not an essential gene but mutant is defective in spore formation; null mutant displays increased levels of spontaneous Rad52p foci | | 2.476019 | 1.308023 | up |
| 1776698_at |  | YLR036C |  | | 2.471414 | 1.305337 | up |
| 1774142_at | GIC2 | YDR309C | Redundant rho-like GTPase Cdc42p effector; homolog of Gic1p; involved in initiation of budding and cellular polarization; interacts with Cdc42p via the Cdc42/Rac-interactive binding (CRIB) domain and with PI(4,5)P2 via a polybasic region | | 2.450578 | 1.293122 | up |
| 1775150_at | NIP7 | YPL211W | Nucleolar protein required for 60S ribosome subunit biogenesis, constituent of 66S pre-ribosomal particles; physically interacts with Nop8p and the exosome subunit Rrp43p | | 2.448482 | 1.291888 | up |
| 1776604_at | CDC9 | YDL164C | DNA ligase found in the nucleus and mitochondria, an essential enzyme that joins Okazaki fragments during DNA replication; also acts in nucleotide excision repair, base excision repair, and recombination | | 2.447683 | 1.291417 | up |
| 1773892_at | NCS2 | YNL119W | Protein required for thiolation of the uridine at the wobble position of Lys(UUU) and Glu(UUC) tRNAs; has a role in urmylation and in invasive and pseudohyphal growth; inhibits replication of Brome mosaic virus in S. cerevisiae | | 2.435643 | 1.284303 | up |
| 1773498_at | MET3 | YJR010W | ATP sulfurylase, catalyzes the primary step of intracellular sulfate activation, essential for assimilatory reduction of sulfate to sulfide, involved in methionine metabolism | | 2.427204 | 1.279295 | up |
| 1779946_at | AAH1 | YNL141W | Adenine deaminase (adenine aminohydrolase), converts adenine to hypoxanthine; involved in purine salvage; transcriptionally regulated by nutrient levels and growth phase; Aah1p degraded upon entry into quiescence via SCF and the proteasome | | 2.426733 | 1.279016 | up |
| 1774822_at | CTF3 | YLR381W | Outer kinetochore protein that forms a complex with Mcm16p and Mcm22p; may bind the kinetochore to spindle microtubules | | 2.424845 | 1.277893 | up |
| 1774858_at | PES4 | YFR023W | Poly(A) binding protein, suppressor of DNA polymerase epsilon mutation, similar to Mip6p | | 2.421645 | 1.275987 | up |
| 1770653_at | RPS0A | YGR214W | Protein component of the small (40S) ribosomal subunit, nearly identical to Rps0Bp; required for maturation of 18S rRNA along with Rps0Bp; deletion of either RPS0 gene reduces growth rate, deletion of both genes is lethal | | 2.417714 | 1.273644 | up |
| 1773767_at | PMS1 | YNL082W | ATP-binding protein required for mismatch repair in mitosis and meiosis; functions as a heterodimer with Mlh1p, binds double- and single-stranded DNA via its N-terminal domain, similar to E. coli MutL | | 2.410074 | 1.269077 | up |
| 1779790_at |  | YNR063W |  | | 2.409649 | 1.268823 | up |
| 1779813_at | RPA49 | YNL248C | RNA polymerase I subunit A49 | | 2.397876 | 1.261757 | up |
| 1777046_at | MRPL4 | YLR439W | Mitochondrial ribosomal protein of the large subunit, homolog of prokaryotic L29 ribosomal protein; located at the ribosomal tunnel exit | | 2.394855 | 1.259938 | up |
| 1776556_at | NDD1 | YOR372C | Transcriptional activator essential for nuclear division; localized to the nucleus; essential component of the mechanism that activates the expression of a set of late-S-phase-specific genes | | 2.388441 | 1.256069 | up |
| 1780161_at | RPL16A | YIL133C | N-terminally acetylated protein component of the large (60S) ribosomal subunit, binds to 5.8 S rRNA; has similarity to Rpl16Bp, E. coli L13 and rat L13a ribosomal proteins; transcriptionally regulated by Rap1p | | 2.373804 | 1.247201 | up |
| 1778128_at | DUR3 | YHL016C | Plasma membrane transporter for both urea and polyamines, expression is highly sensitive to nitrogen catabolite repression and induced by allophanate, the last intermediate of the allantoin degradative pathway | | 2.372778 | 1.246577 | up |
| 1771911_at | RPL20A | YMR242C | Protein component of the large (60S) ribosomal subunit, nearly identical to Rpl20Bp and has similarity to rat L18a ribosomal protein | | 2.366814 | 1.242946 | up |
| 1773459_at | ARX1 | YDR101C | Shuttling pre-60S factor; involved in the biogenesis of ribosomal large subunit biogenesis; interacts directly with Alb1; responsible for Tif6 recycling defects in absence of Rei1; associated with the ribosomal export complex | | 2.361456 | 1.239677 | up |
| 1776370_at | RPL2A | YFR031C-A | Protein component of the large (60S) ribosomal subunit, identical to Rpl2Bp and has similarity to E. coli L2 and rat L8 ribosomal proteins | | 2.356527 | 1.236662 | up |
| 1771531_at | YUR1 | YJL139C | Mannosyltransferase of the KTR1 family, involved in protein N-glycosylation; located in the Golgi apparatus | | 2.346738 | 1.230657 | up |
| 1771565_at | ATC1 | YDR184C | Nuclear protein, possibly involved in regulation of cation stress responses and/or in the establishment of bipolar budding pattern | | 2.331322 | 1.221148 | up |
| 1777990_at | RPS10A | YOR293W | Protein component of the small (40S) ribosomal subunit; nearly identical to Rps10Bp and has similarity to rat ribosomal protein S10 | | 2.3284 | 1.219339 | up |
| 1771906_at | RPS18B | YML026C | Protein component of the small (40S) ribosomal subunit; nearly identical to Rps18Ap and has similarity to E. coli S13 and rat S18 ribosomal proteins | | 2.295418 | 1.198757 | up |
| 1775402_at | SPL2 | YHR136C | Protein with similarity to cyclin-dependent kinase inhibitors; downregulates low-affinity phosphate transport during phosphate limitation; overproduction suppresses a plc1 null mutation; GFP-fusion protein localizes to the cytoplasm | | 2.289396 | 1.194967 | up |
| 1779982_at | RPL32 | YBL092W | Protein component of the large (60S) ribosomal subunit, has similarity to rat L32 ribosomal protein; overexpression disrupts telomeric silencing | | 2.282372 | 1.190534 | up |
| 1778592_at | RPS1A | YLR441C | Ribosomal protein 10 (rp10) of the small (40S) subunit; nearly identical to Rps1Bp and has similarity to rat S3a ribosomal protein | | 2.279106 | 1.188468 | up |
| 1770843_at | IRC10 | YOL015W | Putative protein of unknown function; null mutant displays increased levels of spontaneous Rad52p foci | | 2.277499 | 1.187451 | up |
| 1777640_at |  | YLL054C |  | | 2.276007 | 1.186505 | up |
| 1771764_at | RAS1 | YOR101W | GTPase involved in G-protein signaling in the adenylate cyclase activating pathway, plays a role in cell proliferation; localized to the plasma membrane; homolog of mammalian RAS proto-oncogenes | | 2.275079 | 1.185916 | up |
| 1776193_at | TAT1 | YBR069C | Amino acid transport protein for valine, leucine, isoleucine, and tyrosine, low-affinity tryptophan and histidine transporter; overexpression confers FK506 and FTY720 resistance | | 2.268562 | 1.181778 | up |
| 1778617_at | IMA5 | YJL216C | Alpha-glucosidase with specificity for isomaltose, maltose, and palatinose; member of the IMA isomaltase family; not required for isomaltose utilization, but Ima5p overexpression allows the ima1 null mutant to grow on isomaltose | | 2.268248 | 1.181578 | up |
| 1770277_at | MNL1 | YHR204W | Alpha-1,2-specific exomannosidase of the endoplasmic reticulum; in complex with Pdi1p, generates a Man7GlcNac2 oligosaccharide signal on glycoproteins destined for ubiquitin-proteasome degradation | | 2.260184 | 1.17644 | up |
| 1770887_at |  | YKL187C |  | | 2.256217 | 1.173906 | up |
| 1777214_at |  | YNL162W-A | |  | 2.252641 | 1.171617 | up |
| 1779154_s_at | RPS4B | YHR203C | Protein component of the small (40S) ribosomal subunit; mutation affects 20S pre-rRNA processing; identical to Rps4Bp and has similarity to rat S4 ribosomal protein///Protein component of the small (40S) ribosomal subunit; identical to Rps4Ap and has similarity to rat S4 ribosomal protein | | 2.250526 | 1.170262 | up |
| 1769877_at | RPL34B | YIL052C | Protein component of the large (60S) ribosomal subunit, nearly identical to Rpl34Ap and has similarity to rat L34 ribosomal protein | | 2.244327 | 1.166283 | up |
| 1778691_at | SMD1 | YGR074W | Core Sm protein Sm D1; part of heteroheptameric complex (with Smb1p, Smd2p, Smd3p, Sme1p, Smx3p, and Smx2p) that is part of the spliceosomal U1, U2, U4, and U5 snRNPs; homolog of human Sm D1 | | 2.241258 | 1.164309 | up |
| 1774270_at | TOF1 | YNL273W | Subunit of a replication-pausing checkpoint complex (Tof1p-Mrc1p-Csm3p) that acts at the stalled replication fork to promote sister chromatid cohesion after DNA damage, facilitating gap repair of damaged DNA; interacts with the MCM helicase | | 2.241152 | 1.16424 | up |
| 1772007_at |  | YIL029C |  | | 2.233892 | 1.15956 | up |
| 1777369_at | SHO1 | YER118C | Transmembrane osmosensor involved in activation of the Cdc42p- and MAP kinase-dependent filamentous growth pathway and the high-osmolarity glycerol response pathway; phosphorylated by Hog1p; interacts with Pbs2p, Msb2p, Hkr1p, and Ste11p | | 2.233769 | 1.15948 | up |
| 1773093_at | RKM1 | YPL208W | SET-domain lysine-N-methyltransferase, catalyzes the formation of dimethyllysine residues on the large ribsomal subunit proteins L23a (Rpl23Ap and Rpl23Bp) and L18 (Rps18Ap and Rps18Bp) | | 2.23361 | 1.159378 | up |
| 1770189_at |  | YHR007C-A |  | | 2.221588 | 1.151591 | up |
| 1773168_at | SUT1 | YGL162W | Transcription factor of the Zn[II]2Cys6 family involved in sterol uptake; involved in induction of hypoxic gene expression | | 2.220404 | 1.150822 | up |
| 1773977_at | CPR8 | YNR028W | Peptidyl-prolyl cis-trans isomerase (cyclophilin), catalyzes the cis-trans isomerization of peptide bonds N-terminal to proline residues; similarity to Cpr4p suggests a potential role in the secretory pathway | | 2.212139 | 1.145442 | up |
| 1769919_at | RPL5 | YPL131W | Protein component of the large (60S) ribosomal subunit with similarity to E. coli L18 and rat L5 ribosomal proteins; binds 5S rRNA and is required for 60S subunit assembly | | 2.203005 | 1.139473 | up |
| 1774937_at | PDC5 | YLR134W | Minor isoform of pyruvate decarboxylase, key enzyme in alcoholic fermentation, decarboxylates pyruvate to acetaldehyde, regulation is glucose- and ethanol-dependent, repressed by thiamine, involved in amino acid catabolism | | 2.196627 | 1.13529 | up |
| 1772817_at | NSR1 | YGR159C | Nucleolar protein that binds nuclear localization sequences, required for pre-rRNA processing and ribosome biogenesis | | 2.194849 | 1.134122 | up |
| 1771501_at | DSS4 | YPR017C | Guanine nucleotide dissociation stimulator for Sec4p, functions in the post-Golgi secretory pathway; binds zinc, found both on membranes and in the cytosol | | 2.191874 | 1.132165 | up |
| 1775495_at | MCD1 | YDL003W | Essential subunit of the cohesin complex required for sister chromatid cohesion in mitosis and meiosis; apoptosis induces cleavage and translocation of a C-terminal fragment to mitochondria; expression peaks in S phase | | 2.19065 | 1.131359 | up |
| 1778439_at | RPL23B | YER117W | Protein component of the large (60S) ribosomal subunit, identical to Rpl23Ap and has similarity to E. coli L14 and rat L23 ribosomal proteins | | 2.183947 | 1.126938 | up |
| 1778004_at | PAC11 | YDR488C | Dynein intermediate chain, acts in the cytoplasmic dynein pathway, forms cortical cytoplasmic microtubule capture site with Num1p; null mutant is defective in nuclear migration, essential in the absence of CIN8 | | 2.178356 | 1.12324 | up |
| 1778001_at | DFG16 | YOR030W | Probable multiple transmembrane protein, involved in diploid invasive and pseudohyphal growth upon nitrogen starvation; required for accumulation of processed Rim101p | | 2.178263 | 1.123178 | up |
| 1779963_at |  | YLR287C |  | | 2.178122 | 1.123084 | up |
| 1779914_at |  | YHL044W |  | | 2.176085 | 1.121735 | up |
| 1780200_at | RPS16A | YMR143W | Protein component of the small (40S) ribosomal subunit; identical to Rps16Bp and has similarity to E. coli S9 and rat S16 ribosomal proteins | | 2.175316 | 1.121225 | up |
| 1774947_at | TOS4 | YLR183C | Forkhead Associated domain containing protein and putative transcription factor found associated with chromatin; target of SBF transcription factor; expression is periodic and peaks in G1; similar to PLM2 | | 2.174265 | 1.120528 | up |
| 1778552_at | AIM32 | YML050W | Putative protein of unknown function; null mutant is viable and displays elevated frequency of mitochondrial genome loss | | 2.172955 | 1.119658 | up |
| 1775407_at | RPL38 | YLR325C | Protein component of the large (60S) ribosomal subunit, has similarity to rat L38 ribosomal protein | | 2.171049 | 1.118392 | up |
| 1771269_at | CDC45 | YLR103C | DNA replication initiation factor; recruited to MCM pre-RC complexes at replication origins; promotes release of MCM from Mcm10p, recruits elongation machinery; mutants in human homolog may cause velocardiofacial and DiGeorge syndromes | | 2.16795 | 1.116331 | up |
| 1778153_at |  | YHR022C |  | | 2.158822 | 1.110245 | up |
| 1773412_at | RFA1 | YAR007C | Subunit of heterotrimeric Replication Protein A (RPA), which is a highly conserved single-stranded DNA binding protein involved in DNA replication, repair, and recombination | | 2.15427 | 1.107199 | up |
| 1777199_at | ELP6 | YMR312W | Subunit of hexameric RecA-like ATPase Elp456 Elongator subcomplex; which is required for modification of wobble nucleosides in tRNA; required for Elongator structural integrity | | 2.148906 | 1.103602 | up |
| 1773649_at | FUR4 | YBR021W | Uracil permease, localized to the plasma membrane; expression is tightly regulated by uracil levels and environmental cues | | 2.147927 | 1.102945 | up |
| 1774111_at | CLU1 | YMR012W | eIF3 component of unknown function; deletion causes defects in mitochondrial organization but not in growth or translation initiation, can rescue cytokinesis and mitochondrial organization defects of the Dictyostelium cluA- mutant | | 2.129505 | 1.090518 | up |
| 1776283_at | IZH4 | YOL101C | Membrane protein involved in zinc ion homeostasis, member of the four-protein IZH family, expression induced by fatty acids and altered zinc levels; deletion reduces sensitivity to excess zinc; possible role in sterol metabolism | | 2.126188 | 1.088269 | up |
| 1774275_at | RPS17A | YML024W | Ribosomal protein 51 (rp51) of the small (40s) subunit; nearly identical to Rps17Bp and has similarity to rat S17 ribosomal protein | | 2.120348 | 1.084301 | up |
| 1774371_at | RPL33A | YPL143W | N-terminally acetylated ribosomal protein L37 of the large (60S) ribosomal subunit, nearly identical to Rpl33Bp and has similarity to rat L35a; rpl33a null mutant exhibits slow growth while rpl33a rpl33b double null mutant is inviable | | 2.104569 | 1.073525 | up |
| 1779202_at | MST27 | YGL051W | Putative integral membrane protein, involved in vesicle formation; forms complex with Mst28p; member of DUP240 gene family; binds COPI and COPII vesicles | | 2.08945 | 1.063123 | up |
| 1775457_at | RPL21B | YPL079W | Protein component of the large (60S) ribosomal subunit, nearly identical to Rpl21Ap and has similarity to rat L21 ribosomal protein | | 2.085602 | 1.060464 | up |
| 1774650_at | MSC6 | YOR354C | Protein of unknown function; mutant is defective in directing meiotic recombination events to homologous chromatids; the authentic, non-tagged protein is detected in highly purified mitochondria in high-throughput studies | | 2.078121 | 1.055279 | up |
| 1773482_at | GYP6 | YJL044C | GTPase-activating protein (GAP) for the yeast Rab family member, Ypt6p; involved in vesicle mediated protein transport | | 2.071554 | 1.050714 | up |
| 1772131_s_at |  | YNL033W |  | | 2.067464 | 1.047862 | up |
| 1776049_at | RPS19B | YNL302C | Protein component of the small (40S) ribosomal subunit, required for assembly and maturation of pre-40 S particles; mutations in human RPS19 are associated with Diamond Blackfan anemia; nearly identical to Rps19Ap | | 2.06644 | 1.047148 | up |
| 1779736_at |  | YER039C-A |  | | 2.055699 | 1.039629 | up |
| 1777782_at |  | YOR304C-A |  | | 2.054269 | 1.038625 | up |
| 1777555_at | RPL27A | YHR010W | Protein component of the large (60S) ribosomal subunit, nearly identical to Rpl27Bp and has similarity to rat L27 ribosomal protein | | 2.052621 | 1.037468 | up |
| 1769383_at | MRS1 | YIR021W | Protein required for the splicing of two mitochondrial group I introns (BI3 in COB and AI5beta in COX1); forms a splicing complex, containing four subunits of Mrs1p and two subunits of the BI3-encoded maturase, that binds to the BI3 RNA | | 2.04558 | 1.03251 | up |
| 1769765_at | RPL19A | YBR084C-A | Protein component of the large (60S) ribosomal subunit, nearly identical to Rpl19Bp and has similarity to rat L19 ribosomal protein; rpl19a and rpl19b single null mutations result in slow growth, while the double null mutation is lethal | | 2.036958 | 1.026416 | up |
| 1777358_at | TIF5 | YPR041W | Translation initiation factor eIF5; functions both as a GTPase-activating protein to mediate hydrolysis of ribosome-bound GTP and as a GDP dissociation inhibitor to prevent recycling of eIF2 | | 2.033797 | 1.024176 | up |
| 1769777_at | SPO19 | YPL130W | Meiosis-specific prospore protein; required to produce bending force necessary for proper assembly of the prospore membrane during sporulation; identified as a weak high-copy suppressor of the spo1-1 ts mutation | | 2.021528 | 1.015446 | up |
| 1769511_at | DBP6 | YNR038W | Essential protein involved in ribosome biogenesis; putative ATP-dependent RNA helicase of the DEAD-box protein family | | 2.020538 | 1.01474 | up |
| 1780080_at | HSL7 | YBR133C | Protein arginine N-methyltransferase that exhibits septin and Hsl1p-dependent bud neck localization and periodic Hsl1p-dependent phosphorylation; required along with Hsl1p for bud neck recruitment, phosphorylation, and degradation of Swe1p | | 2.018596 | 1.013352 | up |
| 1778932_at | URB1 | YKL014C | Nucleolar protein required for the normal accumulation of 25S and 5.8S rRNAs, associated with the 27SA2 pre-ribosomal particle; proposed to be involved in the biogenesis of the 60S ribosomal subunit | | 2.008669 | 1.00624 | up |
| 1778852_at | SMA1 | YPL027W | Protein of unknown function involved in the assembly of the prospore membrane during sporulation; interacts with Spo14p | | 2.005323 | 1.003835 | up |
| 1780137_at | PHM6 | YDR281C | Protein of unknown function, expression is regulated by phosphate levels | | 2.004719 | 1.0034 | up |
| 1779782_at | RPS12 | YOR369C | Protein component of the small (40S) ribosomal subunit; has similarity to rat ribosomal protein S12 | | 2.003645 | 1.002627 | up |
| 1772947_at | RPL20B | YOR312C | Protein component of the large (60S) ribosomal subunit, nearly identical to Rpl20Ap and has similarity to rat L18a ribosomal protein | | 2.003589 | 1.002586 | up |
| 1780093_at |  | YML082W |  | | 1.99895 | 0.999242 | up |
| 1770569_at | RPS21A | YKR057W | Protein component of the small (40S) ribosomal subunit; nearly identical to Rps21Bp and has similarity to rat S21 ribosomal protein | | 1.997118 | 0.99792 | up |
| 1774501_at |  | YMR084W |  | | 1.992804 | 0.9948 | up |
| 1777134_at | RPL28 | YGL103W | Ribosomal protein of the large (60S) ribosomal subunit, has similarity to E. coli L15 and rat L27a ribosomal proteins; may have peptidyl transferase activity; can mutate to cycloheximide resistance | | 1.991303 | 0.993713 | up |
| 1772065_at | RPL12B | YDR418W | Protein component of the large (60S) ribosomal subunit, nearly identical to Rpl12Ap; rpl12a rpl12b double mutant exhibits slow growth and slow translation; has similarity to E. coli L11 and rat L12 ribosomal proteins | | 1.99122 | 0.993653 | up |
| 1770690_at | SLM3 | YDL033C | tRNA-specific 2-thiouridylase, responsible for 2-thiolation of the wobble base of mitochondrial tRNAs; human ortholog is implicated in myoclonus epilepsy associated with ragged red fibers (MERRF) | | 1.987873 | 0.991226 | up |
| 1775536_at | RPL22A | YLR061W | Protein component of the large (60S) ribosomal subunit, has similarity to Rpl22Bp and to rat L22 ribosomal protein | | 1.985297 | 0.989355 | up |
| 1777991_at |  | YER184C |  | | 1.976047 | 0.982617 | up |
| 1769663_at | RPS29B | YDL061C | Protein component of the small (40S) ribosomal subunit; nearly identical to Rps29Ap and has similarity to rat S29 and E. coli S14 ribosomal proteins | | 1.974755 | 0.981674 | up |
| 1775162_at | UBP8 | YMR223W | Ubiquitin-specific protease that is a component of the SAGA (Spt-Ada-Gcn5-Acetyltransferase) acetylation complex; required for SAGA-mediated deubiquitination of histone H2B | | 1.973425 | 0.980701 | up |
| 1778638_at | TAM41 | YGR046W | Mitochondrial protein involved in protein import into the mitochondrial matrix; maintains the functional integrity of the TIM23 protein translocator complex; viability of null mutant is strain-dependent; mRNA is targeted to the bud | | 1.970561 | 0.978606 | up |
| 1775052_at | UTP8 | YGR128C | Nucleolar protein required for export of tRNAs from the nucleus; also copurifies with the small subunit (SSU) processome containing the U3 snoRNA that is involved in processing of pre-18S rRNA | | 1.970477 | 0.978545 | up |
| 1778364_at | PPM2 | YOL141W | AdoMet-dependent tRNA methyltransferase also involved in methoxycarbonylation; required for the synthesis of wybutosine (yW), a modified guanosine found at the 3'-position adjacent to the anticodon of phe-tRNA; similarity to Ppm1p | | 1.962982 | 0.973047 | up |
| 1775594_s_at | RPL42B | YHR141C | Protein component of the large (60S) ribosomal subunit, identical to Rpl42Bp and has similarity to rat L44 ribosomal protein///Protein component of the large (60S) ribosomal subunit, identical to Rpl42Ap and has similarity to rat L44; required for propagation of the killer toxin-encoding M1 double-stranded RNA satellite of the L-A double-stranded RNA virus | | 1.96185 | 0.972215 | up |
| 1770974_at | SWM2 | YNR004W | Putative protein of unknown function; haploid disruptant exhibits cold-sensitive growth and elongated buds | | 1.961417 | 0.971897 | up |
| 1779908_at | BIT61 | YJL058C | Subunit of TORC2 (Tor2p-Lst8p-Avo1-Avo2-Tsc11p-Bit61p-Slm1p-Slm2p), a membrane-associated complex that regulates cell cycle-dependent actin cytoskeletal dynamics during polarized growth and cell wall integrity | | 1.948206 | 0.962146 | up |
| 1770680_at | SUA5 | YGL169W | Single-stranded telomeric DNA-binding protein, required for normal telomere length; null mutant lacks N6-threonylcarbamoyl adenosine (t6A) modification in the anticodon loop of ANN-decoding tRNA; member of conserved YrdC/Sua5 family | | 1.942545 | 0.957948 | up |
| 1775706_at | NUP188 | YML103C | Subunit of the inner ring of the nuclear pore complex (NPC); contributes to NPC organization and nucleocytoplasmic transport; homologous to human NUP188 | | 1.932081 | 0.950156 | up |
| 1777885_at | MNT4 | YNR059W | Putative alpha-1,3-mannosyltransferase, not required for protein O-glycosylation | | 1.92713 | 0.946454 | up |
| 1771716_s_at | AAD4 | YDL243C | Putative aryl-alcohol dehydrogenase; similar to P. chrysosporium aryl-alcohol dehydrogenase; mutational analysis has not yet revealed a physiological role///Putative aryl-alcohol dehydrogenase; involved in oxidative stress response; similar to P. chrysosporium aryl-alcohol dehydrogenase; expression induced in cells treated with the mycotoxin patulin | | 1.920237 | 0.941284 | up |
| 1778726_at | TIM13 | YGR181W | Mitochondrial intermembrane space protein, forms a complex with Tim8p that delivers a subset of hydrophobic proteins to the TIM22 complex for insertion into the inner membrane | | 1.912308 | 0.935315 | up |
| 1775807_at | GCD1 | YOR260W | Gamma subunit of the translation initiation factor eIF2B, the guanine-nucleotide exchange factor for eIF2; activity subsequently regulated by phosphorylated eIF2; first identified as a negative regulator of GCN4 expression | | 1.903498 | 0.928653 | up |
| 1770502_x_at | RPS26A | YGL189C | Protein component of the small (40S) ribosomal subunit; nearly identical to Rps26Bp and has similarity to rat S26 ribosomal protein | | 1.90061 | 0.926462 | up |
| 1773507_at |  | YOR214C |  | | 1.899406 | 0.925548 | up |
| 1779373_at | POC4 | YPL144W | Component of a heterodimeric Poc4p-Irc25p chaperone involved in assembly of alpha subunits into the 20S proteasome; may regulate formation of proteasome isoforms with alternative subunits under different conditions | | 1.897844 | 0.924361 | up |
| 1772317_at | MES1 | YGR264C | Methionyl-tRNA synthetase, forms a complex with glutamyl-tRNA synthetase (Gus1p) and Arc1p, which increases the catalytic efficiency of both tRNA synthetases; also has a role in nuclear export of tRNAs | | 1.892103 | 0.919991 | up |
| 1775201_at |  | YPL257W |  | | 1.882397 | 0.912571 | up |
| 1778979_at | MBA1 | YBR185C | Membrane-associated mitochondrial ribosome receptor; forms a complex with Mdm38p that may facilitate recruitment of mRNA-specific translational activators to ribosomes; possible role in protein export from the matrix to inner membrane | | 1.87704 | 0.908459 | up |
| 1775775_at | DPH1 | YIL103W | Protein required, along with Dph2p, Kti11p, Jjj3p, and Dph5p, for synthesis of diphthamide, which is a modified histidine residue of translation elongation factor 2 (Eft1p or Eft2p); may act in a complex with Dph2p and Kti11p | | 1.876444 | 0.908001 | up |
| 1774491_s_at | MAL32 | YBR299W | Maltase (alpha-D-glucosidase), inducible protein involved in maltose catabolism; encoded in the MAL1 complex locus; hydrolyzes the disaccharides maltose, turanose, maltotriose, and sucrose///Maltase (alpha-D-glucosidase), inducible protein involved in maltose catabolism; encoded in the MAL3 complex locus; functional in genomic reference strain S288C; hydrolyzes the disaccharides maltose, turanose, maltotriose, and sucrose | | 1.873309 | 0.905589 | up |
| 1770636_at | DOC1 | YGL240W | Processivity factor required for the ubiquitination activity of the anaphase promoting complex (APC), mediates the activity of the APC by contributing to substrate recognition; involved in cyclin proteolysis; contains a conserved DOC1 homology domain | | 1.869714 | 0.902818 | up |
| 1777328_at |  | YJL127C-B |  | | 1.865829 | 0.899817 | up |
| 1776118_at | UTP21 | YLR409C | Subunit of U3-containing 90S preribosome and Small Subunit (SSU) processome complexes involved in production of 18S rRNA and assembly of small ribosomal subunit; synthetic defect with STI1 Hsp90 cochaperone; human homolog linked to glaucoma | | 1.865477 | 0.899545 | up |
| 1778078_at | BTS1 | YPL069C | Geranylgeranyl diphosphate synthase, increases the intracellular pool of geranylgeranyl diphosphate, suppressor of bet2 mutation that causes defective geranylgeranylation of small GTP-binding proteins that mediate vesicular traffic | | 1.856931 | 0.89292 | up |
| 1773826_at | ALK2 | YBL009W | Protein kinase; accumulation and phosphorylation are periodic during the cell cycle; phosphorylated in response to DNA damage; contains characteristic motifs for degradation via the APC pathway; similar to Alk1p and to mammalian haspins | | 1.854275 | 0.890855 | up |
| 1769810_at | EBS1 | YDR206W | Protein involved in inhibition of translation and nonsense-mediated decay; interacts with cap binding protein Cdc33p and with Nam7p; localizes to P-bodies upon glucose starvation; mRNA abundance regulated by mRNA decay factors | | 1.84533 | 0.883879 | up |
| 1775448_at | RPL37B | YDR500C | Protein component of the large (60S) ribosomal subunit, has similarity to Rpl37Ap and to rat L37 ribosomal protein | | 1.837949 | 0.878097 | up |
| 1778154_at | IRC18 | YJL037W | Putative protein of unknown function; expression induced in respiratory-deficient cells and in carbon-limited chemostat cultures; similar to adjacent ORF, YJL038C; null mutant displays increased levels of spontaneous Rad52p foci | | 1.831196 | 0.872787 | up |
| 1775568_at |  | YMR259C |  | | 1.827598 | 0.869949 | up |
| 1775412_at | SHU2 | YDR078C | Component of the Shu complex, which promotes error-free DNA repair; Shu complex mediates inhibition of Srs2p function | | 1.824359 | 0.86739 | up |
| 1775975_at | MMT2 | YPL224C | Putative metal transporter involved in mitochondrial iron accumulation; closely related to Mmt1p | | 1.819845 | 0.863816 | up |
| 1774974_at | IMD4 | YML056C | Inosine monophosphate dehydrogenase, catalyzes the first step of GMP biosynthesis, member of a four-gene family in S. cerevisiae, constitutively expressed | | 1.819671 | 0.863678 | up |
| 1773124_at |  | YDR179W-A |  | | 1.817768 | 0.862168 | up |
| 1771090_at | FRE2 | YKL220C | Ferric reductase and cupric reductase, reduces siderophore-bound iron and oxidized copper prior to uptake by transporters; expression induced by low iron levels but not by low copper levels | | 1.816586 | 0.861229 | up |
| 1770200_at | YTM1 | YOR272W | Constituent of 66S pre-ribosomal particles, forms a complex with Nop7p and Erb1p that is required for maturation of the large ribosomal subunit; has seven C-terminal WD repeats | | 1.803187 | 0.850549 | up |
| 1770876_at | NOP14 | YDL148C | Nucleolar protein, forms a complex with Noc4p that mediates maturation and nuclear export of 40S ribosomal subunits; also present in the small subunit processome complex, which is required for processing of pre-18S rRNA | | 1.799248 | 0.847394 | up |
| 1770404_at | RRT2 | YBR246W | Protein required for last step of diphthamide biosynthesis; deletion leads to accumulation of diphthine, which is the enzymatic product of the second step of the biosynthesis; WD40 domain-containing protein involved in endosomal recycling; forms a complex with Rtt10p that functions in the retromer-mediated pathway for recycling internalized cell-surface proteins; non-essential gene identified in a screen for mutants with increased levels of rDNA transcription | | 1.784912 | 0.835853 | up |
| 1770098_at | PGA3 | YML125C | Putative cytochrome b5 reductase, localized to the plasma membrane; may be involved in regulation of lifespan; required for maturation of Gas1p and Pho8p, proposed to be involved in protein trafficking | | 1.780965 | 0.832659 | up |
| 1777761_at | TOD6 | YBL054W | PAC motif binding protein involved in rRNA and ribosome biogenesis; subunit of the RPD3L histone deacetylase complex; Myb-like HTH transcription factor, similar to Dot6p; hypophosphorylated by rapamycin treatment in a Sch9p-dependent manne | | 1.750509 | 0.807774 | up |
| 1774978_at | PDR17 | YNL264C | Phosphatidylinositol transfer protein (PITP), downregulates Plb1p-mediated turnover of phosphatidylcholine, found in the cytosol and microsomes, homologous to Pdr16p, deletion affects phospholipid composition | | 1.749141 | 0.806646 | up |
| 1773741_at | SEE1 | YIL064W | Probable lysine methyltransferase involved in the dimethylation of eEF1A (Tef1p/Tef2p); sequence similarity to S-adenosylmethionine-dependent methyltransferases of the seven beta-strand family; role in vesicular transport | | 1.730287 | 0.791011 | up |
| 1770109_at | RPL14B | YHL001W | Protein component of the large (60S) ribosomal subunit, nearly identical to Rpl14Ap and has similarity to rat L14 ribosomal protein | | 1.727528 | 0.788709 | up |
| 1769311_at |  | YDL157C |  | | 1.725493 | 0.787009 | up |
| 1776151_at | MTO1 | YGL236C | Mitochondrial protein, forms a heterodimer complex with Mss1p that performs the 5-carboxymethylaminomethyl modification of the wobble uridine base in mitochondrial tRNAs; required for respiration in paromomycin-resistant 15S rRNA mutants | | 1.714587 | 0.777861 | up |
| 1772958_at | FMP48 | YGR052W | Putative protein of unknown function; the authentic, non-tagged protein is detected in highly purified mitochondria in high-throughput studies; induced by treatment with 8-methoxypsoralen and UVA irradiation | | 1.714332 | 0.777647 | up |
| 1771658_at | NIP1 | YMR309C | eIF3c subunit of the eukaryotic translation initiation factor 3 (eIF3), involved in the assembly of preinitiation complex and start codon selection | | 1.710275 | 0.774228 | up |
| 1775425_at | RAD10 | YML095C | Single-stranded DNA endonuclease (with Rad1p), cleaves single-stranded DNA during nucleotide excision repair and double-strand break repair; subunit of Nucleotide Excision Repair Factor 1 (NEF1); homolog of human ERCC1 protein | | 1.699449 | 0.765067 | up |
| 1770144_at | GAS2 | YLR343W | 1,3-beta-glucanosyltransferase, involved with Gas4p in spore wall assembly; has similarity to Gas1p | | 1.697504 | 0.763415 | up |
| 1777903_at | PHO90 | YJL198W | Low-affinity phosphate transporter; deletion of pho84, pho87, pho89, pho90, and pho91 causes synthetic lethality; transcription independent of Pi and Pho4p activity; overexpression results in vigorous growth | | 1.691933 | 0.758673 | up |
| 1770612_at | RPL24B | YGR148C | Ribosomal protein L30 of the large (60S) ribosomal subunit, nearly identical to Rpl24Ap and has similarity to rat L24 ribosomal protein; not essential for translation but may be required for normal translation rate | | 1.679183 | 0.74776 | up |
| 1773050_at | CLG1 | YGL215W | Cyclin-like protein that interacts with Pho85p; has sequence similarity to G1 cyclins PCL1 and PCL2 | | 1.678685 | 0.747332 | up |
| 1772975_at | RPS3 | YNL178W | Protein component of the small (40S) ribosomal subunit, has apurinic/apyrimidinic (AP) endonuclease activity; essential for viability; has similarity to E. coli S3 and rat S3 ribosomal proteins | | 1.677646 | 0.746438 | up |
| 1773889_at |  | YOL131W |  | | 1.674393 | 0.743638 | up |
| 1771390_at | MCM5 | YLR274W | Component of the hexameric MCM complex, which is important for priming origins of DNA replication in G1 and becomes an active ATP-dependent helicase that promotes DNA melting and elongation when activated by Cdc7p-Dbf4p in S-phase | | 1.653338 | 0.725382 | up |
| 1773154_at | RCL1 | YOL010W | Endonuclease that cleaves pre-rRNA at site A2 for 18S rRNA biogenesis; subunit of U3-containing 90S preribosome processome complex involved in small ribosomal subunit assembly; stimulates Bms1p GTPase and U3 binding activity; similar to RNA cyclase-like proteins but no cyclase activity detected | | 1.649713 | 0.722215 | up |
| 1776863_at | SWH1 | YAR042W | Protein similar to mammalian oxysterol-binding protein; contains ankyrin repeats; localizes to the Golgi and the nucleus-vacuole junction | | -1.69311 | -0.75967 | down |
| 1778930_at |  | YOR289W |  | | -1.71144 | -0.77521 | down |
| 1777854_at | PDE1 | YGL248W | Low-affinity cyclic AMP phosphodiesterase, controls glucose and intracellular acidification-induced cAMP signaling, target of the cAMP-protein kinase A (PKA) pathway; glucose induces transcription and inhibits translation | | -1.73936 | -0.79856 | down |
| 1769779_at | ABP1 | YCR088W | Actin-binding protein of the cortical actin cytoskeleton, important for activation of the Arp2/3 complex that plays a key role actin in cytoskeleton organization; phosphorylation within its PRR (Proline-Rich Region), mediated by Cdc28p and Pho85p, protects Abp1p from proteolysis mediated by its own PEST sequences | | -1.75517 | -0.81161 | down |
| 1778966_at | CLC1 | YGR167W | Clathrin light chain, subunit of the major coat protein involved in intracellular protein transport and endocytosis; thought to regulate clathrin function; two Clathrin heavy chains (CHC1) form the clathrin triskelion structural component | | -1.75607 | -0.81235 | down |
| 1771150_at |  | YPL107W |  | | -1.76554 | -0.82011 | down |
| 1770721_at | FCY1 | YPR062W | Cytosine deaminase, zinc metalloenzyme that catalyzes the hydrolytic deamination of cytosine to uracil; of biomedical interest because it also catalyzes the deamination of 5-fluorocytosine (5FC) to form anticancer drug 5-fluorouracil (5FU) | | -1.76863 | -0.82263 | down |
| 1770399_at | ZEO1 | YOL109W | Peripheral membrane protein of the plasma membrane that interacts with Mid2p; regulates the cell integrity pathway mediated by Pkc1p and Slt2p; the authentic protein is detected in a phosphorylated state in highly purified mitochondria | | -1.81687 | -0.86145 | down |
| 1778960_at | TEC1 | YBR083W | Transcription factor targeting filamentation genes and Ty1 expression; Ste12p activation of most filamentation gene promoters depends on Tec1p and Tec1p transcriptional activity is dependent on its association with Ste12p; binds to TCS elements upstream of filamentation genes, which are regulated by Tec1p/Ste12p/Dig1p complex; competes with Dig2p for binding to Ste12p/Dig1p; positive regulator of chronological life span; TEA/ATTS DNA-binding domain family member | | -1.82367 | -0.86684 | down |
| 1779897_at |  | YNR034W-A |  | | -1.82577 | -0.8685 | down |
| 1777366_at | PIG2 | YIL045W | Putative type-1 protein phosphatase targeting subunit that tethers Glc7p type-1 protein phosphatase to Gsy2p glycogen synthase | | -1.82649 | -0.86907 | down |
| 1773666_at | PIH1 | YHR034C | Component of the conserved R2TP complex (Rvb1-Rvb2-Tah1-Pih1); R2TP complex interacts with Hsp90 (Hsp82p and Hsc82p) to mediate assembly large protein complexes such as box C/D snoRNPs and RNA polymerase II | | -1.83796 | -0.87811 | down |
| 1770279_at | LYS1 | YIR034C | Saccharopine dehydrogenase (NAD+, L-lysine-forming), catalyzes the conversion of saccharopine to L-lysine, which is the final step in the lysine biosynthesis pathway; also has mRNA binding activity | | -1.85844 | -0.89409 | down |
| 1773831_at |  | YMR114C |  | | -1.86263 | -0.89734 | down |
| 1777053_at | ATH1 | YPR026W | Acid trehalase required for utilization of extracellular trehalose | | -1.87319 | -0.9055 | down |
| 1770170_at | RAD2 | YGR258C | Single-stranded DNA endonuclease, cleaves single-stranded DNA during nucleotide excision repair to excise damaged DNA; subunit of Nucleotide Excision Repair Factor 3 (NEF3); homolog of human XPG protein | | -1.87906 | -0.91001 | down |
| 1771348_at |  | YLR040C |  | | -1.88645 | -0.91567 | down |
| 1770958_at | UBX7 | YBR273C | UBX (ubiquitin regulatory X) domain-containing protein that interacts with Cdc48p | | -1.88752 | -0.91649 | down |
| 1774489_at |  | YDR248C |  | | -1.88817 | -0.91699 | down |
| 1779010_at | SIC1 | YLR079W | Cyclin-dependent kinase inhibitor (CKI); inhibitor of Cdc28-Clb kinase complexes that controls G1/S phase transition, preventing premature S phase and ensuring genomic integrity; phosphorylation targets Sic1p for SCF(CDC4)-dependent turnover; functional homolog of mammalian Kip1 | | -1.89122 | -0.91931 | down |
| 1770941_at | ELM1 | YKL048C | Serine/threonine protein kinase that regulates cellular morphogenesis, septin behavior, and cytokinesis; required for the regulation of other kinases; forms part of the bud neck ring | | -1.89361 | -0.92114 | down |
| 1779219_at | SNF7 | YLR025W | One of four subunits of the endosomal sorting complex required for transport III (ESCRT-III); involved in the sorting of transmembrane proteins into the multivesicular body (MVB) pathway; recruited from the cytoplasm to endosomal membranes | | -1.91621 | -0.93825 | down |
| 1779924_at |  | YER053C-A |  | | -1.92629 | -0.94583 | down |
| 1772019_at | BNA1 | YJR025C | 3-hydroxyanthranilic acid dioxygenase, required for the de novo biosynthesis of NAD from tryptophan via kynurenine; expression regulated by Hst1p | | -1.97552 | -0.98223 | down |
| 1773109_at | DAL80 | YKR034W | Negative regulator of genes in multiple nitrogen degradation pathways; expression is regulated by nitrogen levels and by Gln3p; member of the GATA-binding family, forms homodimers and heterodimers with Deh1p | | -2.00203 | -1.00146 | down |
| 1779364_at | RTC2 | YBR147W | Protein of unknown function; mutant produces large lipid droplets, is resistant to fluconazole, has decreased levels of rDNA transcription, growth defects on minimal media, and suppresses cdc13-1; detected in highly purified mitochondria; similar to a G-protein coupled receptor from S. pombe | | -2.01225 | -1.00881 | down |
| 1773560_at | RIB1 | YBL033C | GTP cyclohydrolase II; catalyzes the first step of the riboflavin biosynthesis pathway | | -2.01486 | -1.01068 | down |
| 1778437_at | AFR1 | YDR085C | Protein required for pheromone-induced projection (shmoo) formation; regulates septin architecture during mating; has an RVXF motif that mediates targeting of Glc7p to mating projections; interacts with Cdc12p | | -2.02204 | -1.01581 | down |
| 1771265_at | FMP46 | YKR049C | Putative redox protein containing a thioredoxin fold; the authentic, non-tagged protein is detected in highly purified mitochondria in high-throughput studies | | -2.07181 | -1.05089 | down |
| 1771109_at | WSC4 | YHL028W | ER membrane protein involved in the translocation of soluble secretory proteins and insertion of membrane proteins into the ER membrane; may also have a role in the stress response but has only partial functional overlap with WSC1-3 | | -2.09887 | -1.06961 | down |
| 1770010_at | PAR32 | YDL173W | Putative protein of unknown function; hyperphosphorylated upon rapamycin treatment in a Tap42p-dependent manner; green fluorescent protein (GFP)-fusion protein localizes to the cytoplasm; PAR32 is not an essential gene | | -2.10124 | -1.07124 | down |
| 1771188_at |  | YNL217W |  | | -2.12348 | -1.08643 | down |
| 1774070_at | ADH2 | YMR303C | Glucose-repressible alcohol dehydrogenase II, catalyzes the conversion of ethanol to acetaldehyde; involved in the production of certain carboxylate esters; regulated by ADR1 | | -2.14318 | -1.09976 | down |
| 1773931_at | NRG2 | YBR066C | Transcriptional repressor that mediates glucose repression and negatively regulates filamentous growth; has similarity to Nrg1p | | -2.15978 | -1.11089 | down |
| 1772778_at | ATG8 | YBL078C | Component of autophagosomes and Cvt vesicles; undergoes conjugation to phosphatidylethanolamine (PE); Atg8p-PE is anchored to membranes, is involved in phagophore expansion, and may mediate membrane fusion during autophagosome formation | | -2.16938 | -1.11728 | down |
| 1776664_at | DAK2 | YFL053W | Dihydroxyacetone kinase, required for detoxification of dihydroxyacetone (DHA); involved in stress adaptation | | -2.18458 | -1.12735 | down |
| 1776372_at | APS1 | YLR170C | Small subunit of the clathrin-associated adaptor complex AP-1, which is involved in protein sorting at the trans-Golgi network; homolog of the sigma subunit of the mammalian clathrin AP-1 complex | | -2.19571 | -1.13469 | down |
| 1769833_at | OPI1 | YHL020C | Transcriptional regulator of a variety of genes; phosphorylation by protein kinase A stimulates Opi1p function in negative regulation of phospholipid biosynthetic genes; involved in telomere maintenance | | -2.20584 | -1.14133 | down |
| 1780036_at | UBC8 | YEL012W | Ubiquitin-conjugating enzyme that negatively regulates gluconeogenesis by mediating the glucose-induced ubiquitination of fructose-1,6-bisphosphatase (FBPase); cytoplasmic enzyme that catalyzes the ubiquitination of histones in vitro | | -2.20777 | -1.14259 | down |
| 1777966_at | PNG1 | YPL096W | Conserved peptide N-glycanase required for deglycosylation of misfolded glycoproteins during proteasome-dependent degradation; localizes to the cytoplasm and nucleus; activity is enhanced by interaction with Rad23p | | -2.22485 | -1.15371 | down |
| 1774929_at | CHS5 | YLR330W | Component of the exomer complex, which also contains Csh6p, Bch1p, Bch2p, and Bud7p and is involved in export of selected proteins, such as chitin synthase Chs3p, from the Golgi to the plasma membrane | | -2.25696 | -1.17438 | down |
| 1770150_at | YAF9 | YNL107W | Subunit of both the NuA4 histone H4 acetyltransferase complex and the SWR1 complex, may function to antagonize silencing near telomeres; interacts directly with Swc4p, has homology to human leukemogenic protein AF9, contains a YEATS domain | | -2.26194 | -1.17756 | down |
| 1773745_at | SLA2 | YNL243W | Transmembrane actin-binding protein involved in membrane cytoskeleton assembly and cell polarization; adaptor protein that links actin to clathrin and endocytosis; present in the actin cortical patch of the emerging bud tip; dimer in vivo | | -2.29588 | -1.19904 | down |
| 1772972_at | ESC1 | YMR219W | Protein localized to the nuclear periphery, involved in telomeric silencing; interacts with PAD4-domain of Sir4p | | -2.30049 | -1.20194 | down |
| 1775918_at | BUR6 | YER159C | Subunit of a heterodimeric NC2 transcription regulator complex with Ncb2p; complex binds to TBP and can repress transcription by preventing preinitiation complex assembly or stimulate activated transcription; homologous to human NC2alpha | | -2.30791 | -1.20659 | down |
| 1775350_at | SUB1 | YMR039C | Transcriptional coactivator, facilitates elongation through factors that modify RNAP II; role in peroxide resistance involving Rad2p; role in the hyperosmotic stress response through polymerase recruitment at RNAP II and RNAP III genes | | -2.33329 | -1.22237 | down |
| 1776528_at | GPM2 | YDL021W | Homolog of Gpm1p phosphoglycerate mutase, which converts 3-phosphoglycerate to 2-phosphoglycerate in glycolysis; may be non-functional derivative of a gene duplication event | | -2.34326 | -1.22852 | down |
| 1769474_at |  | YPL264C |  | | -2.36388 | -1.24116 | down |
| 1772443_at | PEP12 | YOR036W | Target membrane receptor (t-SNARE) for vesicular intermediates traveling between the Golgi apparatus and the vacuole; controls entry of biosynthetic, endocytic, and retrograde traffic into the prevacuolar compartment; syntaxin | | -2.39427 | -1.25958 | down |
| 1773893_at | RHO5 | YNL180C | Non-essential small GTPase of the Rho/Rac subfamily of Ras-like proteins, likely involved in protein kinase C (Pkc1p)-dependent signal transduction pathway that controls cell integrity | | -2.40635 | -1.26684 | down |
| 1769657_at | DSE3 | YOR264W | Daughter cell-specific protein, may help establish daughter fate | | -2.41477 | -1.27189 | down |
| 1774749_at | AAD10 | YJR155W | Putative aryl-alcohol dehydrogenase; similar to P. chrysosporium aryl-alcohol dehydrogenase; mutational analysis has not yet revealed a physiological role | | -2.45041 | -1.29302 | down |
| 1775392_at | YPI1 | YFR003C | Regulatory subunit of the type I protein phosphatase (PP1) Glc7p; Glc7p participates in the regulation of a variety of metabolic processes including mitosis and glycogen metabolism; in vitro evidence suggests Ypi1p is an inhibitor of Glc7p while in vivo evidence suggests it is an activator; overproduction causes decreased cellular content of glycogen; partial depletion causes lithium sensitivity, while overproduction confers lithium-tolerance | | -2.48814 | -1.31507 | down |
| 1774060_at | SCS22 | YBL091C-A | Protein involved in regulation of phospholipid metabolism; homolog of Scs2p; similar to D. melanogaster inturned protein | | -2.4992 | -1.32147 | down |
| 1770660_at | NCB2 | YDR397C | Subunit of a heterodimeric NC2 transcription regulator complex with Bur6p; complex binds to TBP and can repress transcription by preventing preinitiation complex assembly or stimulate activated transcription; homologous to human NC2beta | | -2.54655 | -1.34855 | down |
| 1777623_at | NHA1 | YLR138W | Na+/H+ antiporter involved in sodium and potassium efflux through the plasma membrane; required for alkali cation tolerance at acidic pH | | -2.60178 | -1.3795 | down |
| 1777654_at | BNI1 | YNL271C | Formin, nucleates the formation of linear actin filaments, involved in cell processes such as budding and mitotic spindle orientation which require the formation of polarized actin cables, functionally redundant with BNR1 | | -2.73886 | -1.45358 | down |
| 1778218_at | TMA10 | YLR327C | Protein of unknown function that associates with ribosomes; putative homolog of the F1F0-ATPase synthase regulator Stf2p | | -2.75066 | -1.45978 | down |
| 1778283_at |  | YFL034W |  | | -2.84344 | -1.50764 | down |
| 1774122_at | FDH1 | YOR388C | NAD(+)-dependent formate dehydrogenase, may protect cells from exogenous formate | | -2.856 | -1.514 | down |
| 1776473_at | SEC9 | YGR009C | t-SNARE protein important for fusion of secretory vesicles with the plasma membrane; similar to but not functionally redundant with Spo20p; SNAP-25 homolog | | -2.87076 | -1.52143 | down |
| 1772327_at | PTK2 | YJR059W | Putative serine/threonine protein kinase involved in regulation of ion transport across plasma membrane; enhances spermine uptake | | -3.06611 | -1.61641 | down |
| 1775057_at | MF(ALPHA)1 | YPL187W | Mating pheromone alpha-factor, made by alpha cells; interacts with mating type a cells to induce cell cycle arrest and other responses leading to mating; also encoded by MF(ALPHA)2, although MF(ALPHA)1 produces most alpha-factor | | -3.07189 | -1.61913 | down |
| 1771645_at | SUE1 | YPR151C | Mitochondrial protein required for degradation of unstable forms of cytochrome c | | -3.10776 | -1.63588 | down |
| 1777753_at | RGI1 | YER067W | Protein of unknown function involved in energy metabolism under respiratory conditions; protein abundance is increased upon intracellular iron depletion | | -3.17026 | -1.6646 | down |
| 1772452_at | VHS3 | YOR054C | Negative regulatory subunit of protein phosphatase 1 Ppz1p and also a subunit of the phosphopantothenoylcysteine decarboxylase (PPCDC; Cab3p, Sis2p, Vhs3p) complex, which catalyzes the third step of coenzyme A biosynthesis | | -3.25639 | -1.70328 | down |
| 1775713_at | MET4 | YNL103W | Leucine-zipper transcriptional activator, responsible for the regulation of the sulfur amino acid pathway, requires different combinations of the auxiliary factors Cbf1p, Met28p, Met31p and Met32p | | -3.26856 | -1.70866 | down |
| 1778541_at | PCL9 | YDL179W | Cyclin, forms a functional kinase complex with Pho85p cyclin-dependent kinase (Cdk), expressed in late M/early G1 phase, activated by Swi5p | | -3.53035 | -1.81981 | down |
| 1774263_at | EGT2 | YNL327W | Glycosylphosphatidylinositol (GPI)-anchored cell wall endoglucanase required for proper cell separation after cytokinesis, expression is activated by Swi5p and tightly regulated in a cell cycle-dependent manner | | -3.74818 | -1.90619 | down |
| 1776820_at |  | YHR080C |  | | -3.99588 | -1.99851 | down |
| 1770650_at | IRC4 | YDR540C | Putative protein of unknown function; null mutant displays increased levels of spontaneous Rad52p foci; green fluorescent protein (GFP)-fusion protein localizes to the cytoplasm and nucleus | | -4.10316 | -2.03673 | down |
| 1769988_at | DSE4 | YNR067C | Daughter cell-specific secreted protein with similarity to glucanases, degrades cell wall from the daughter side causing daughter to separate from mother | | -4.15488 | -2.05481 | down |
| 1772199_at | BIO3 | YNR058W | 7,8-diamino-pelargonic acid aminotransferase (DAPA), catalyzes the second step in the biotin biosynthesis pathway; BIO3 is in a cluster of 3 genes (BIO3, BIO4, and BIO5) that mediate biotin synthesis | | -4.56965 | -2.19208 | down |
| 1778564_at | AIM44 | YPL158C | Protein of unknown function; GFP-fusion protein localizes to the bud neck; transcription is regulated by Swi5p; null mutant displays elevated frequency of mitochondrial genome loss | | -5.98778 | -2.58202 | down |
| 1772916_at | BIO4 | YNR057C | Dethiobiotin synthetase, catalyzes the third step in the biotin biosynthesis pathway; BIO4 is in a cluster of 3 genes (BIO3, BIO4, and BIO5) that mediate biotin synthesis; expression appears to be repressed at low iron levels | | -9.44853 | -3.24009 | down |
| 1777152_at | BIO5 | YNR056C | Putative transmembrane protein involved in the biotin biosynthesis pathway; responsible for uptake of 7-keto 8-aminopelargonic acid; BIO5 is in a cluster of 3 genes (BIO3, BIO4, and BIO5) that mediate biotin synthesis | | -18.4179 | -4.20303 | down |
