# Supplementary material for: A systematic assessment of chemical, genetic, and epigenetic factors influencing the activity of anticancer drug KP1019 (FFC14A)
Source: Oncotarget. 2017 Sep 30;8(58):98426–54. doi: 10.18632/oncotarget.21416 (PMC5716741; doi:10.18632/oncotarget.21416)
Supplement: Supplementary file 4 [file oncotarget-08-98426-s004.docx]

**Supplementary Table 3: Complete list of significantly (p<0.1) over-represented MIPS (The Munich Information Center for Protein Sequences) functional categories in the dataset of KP1019 induced transcriptome (>1.5 fold) obtained by FunSpec bioinformatics tool**

| **Category** | **p-value** | **In Category from Cluster** | **k** | **f** |
| --- | --- | --- | --- | --- |
| ribosomal proteins [12.01.01] | 1.00E-14 | RPS8A RPL23A RPL32 RPS11B RPL19A RPS9B RPL21A RPS29B RPL13A RPS16B RPS11A RPL12B RPS17B RPL27B RPL37B RPL12A RPL34A RPS24A RPS8B RPL23B RPL2A RPL24A RPL7A RPL28 RPL9A RPS26A RPL24B NSR1 RPS0A RPL14B RPS20 RPL8A RPL27A RPL42B RPS4B RPL2B RPL34B RPS24B RPL16A RPS21B RPL17B RPS22A RPS14B RPS5 RPL14A RPS21A RPL40B RPS0B RPL22A RPL37A RPS28B RPL38 RPL26A RPS22B MRPL4 RPS1A RPL6B RPS17A RPS18B RPS1B RPL6A RPL13B RPS16A RPL20A RPL9B RPL16B RPS7B RPS3 RPL18B RPS19B DBP6 RPS15 RPL18A RPL3 RPS7A RPL33B RPS10A RPL20B RPS12 RPL21B RPS9A RPL5 RPL33A NIP7 RPS23B | 85 | 246 |
| RNA binding [16.03.03] | 0.000148 | NOP14 RPL24A RPL28 SMD1 UTP8 RPL24B NSR1 RPL14B RPL16A MRS1 RPS14B RPL14A RRN5 RPL26A UTP21 RPL6B RPL6A RPL16B RPL5 NOC4 | 20 | 189 |
| DNA repair [10.01.05.01] | 0.003132 | RFA1 MSH3 CDC9 MSH6 DIN7 PES4 RAD54 RNR3 NSE5 HUG1 RAD10 PMS1 POL2 RFA2 RAD53 | 15 | 159 |
| extension/ polymerization activity [10.01.03.05] | 0.004373 | RFA1 POL12 CDC9 CDC45 POL2 RFA2 | 6 | 37 |
| DNA damage response [32.01.09] | 0.005239 | RFA1 MSH3 EBS1 RAD54 RFX1 HUG1 RAD10 RPS3 POL2 | 9 | 77 |
| translational control [12.07] | 0.008413 | RPS11B RPS9B RPS11A MTO1 RPS9A TIF5 RPS23B | 7 | 55 |
| ribosome biogenesis [12.01] | 0.018628 | NOP14 ARX1 URB2 NCS2 YTM1 TIF5 NOC4 | 7 | 64 |
| ori recognition and priming complex formation [10.01.03.03] | 0.020278 | POL12 CDC6 CDC45 MCM5 | 4 | 25 |
| conjunction of sulfate [01.02.03.04] | 0.042708 | MET3 | 1 | 1 |
| homeostasis of anions [34.01.03] | 0.042708 | DUR3 | 1 | 1 |
| nucleotide/nucleoside/nucleobase transport [20.01.17] | 0.051319 | FUI1 FUR4 RIM2 | 3 | 20 |
| somatic / mitotic recombination [10.01.05.03.03] | 0.058045 | MSH3 RAD54 RAD10 | 3 | 21 |
| cell cycle checkpoints (checkpoints of morphogenesis, DNA-damage,-replication, mitotic phase and spindle) [10.03.01.03] | 0.065337 | RFA1 MSH3 HUG1 POL2 TOF1 RAD53 | 6 | 67 |
| DNA topology [10.01.02] | 0.079289 | RFA1 RAD54 MCM5 TOF1 RFA2 | 5 | 54 |
| C-compound binding [16.13] | 0.083598 | MNL1 | 1 | 2 |
| METABOLISM [01] | 0.083598 | DIT1 | 1 | 2 |
| toxins [32.05.05.01] | 0.083598 | DPH1 | 1 | 2 |
| translation initiation [12.04.01] | 0.089539 | CLU1 NIP1 GCD1 TIF5 | 4 | 40 |
| deoxyribonucleotide metabolism [01.03.07] | 0.090477 | RNR1 RNR3 | 2 | 12 |
| posttranslational modification of amino acids (e.g. hydroxylation, methylation) [14.07.09] | 0.097294 | HSL7 DPH1 RKM1 | 3 | 26 |

‘k’ indicates the number of genes from input cluster in given category whereas ‘f’ indicates the total number of genes in given category.
