# Supplementary material for: A systematic assessment of chemical, genetic, and epigenetic factors influencing the activity of anticancer drug KP1019 (FFC14A)
Source: Oncotarget. 2017 Sep 30;8(58):98426–54. doi: 10.18632/oncotarget.21416 (PMC5716741; doi:10.18632/oncotarget.21416)
Supplement: Supplementary file 5 [file oncotarget-08-98426-s005.docx]

**Supplementary Table 4: Complete list of significantly (p<0.1) over-represented MIPS (The Munich Information Center for Protein Sequences) functional categories in the dataset of KP1019 repressed transcriptome (>1.5 fold) obtained by FunSpec bioinformatics tool**

| **Category** | **p-value** | **In Category from Cluster** | **k** | **f** |
| --- | --- | --- | --- | --- |
| regulator of transcription factor [18.02.09] | 5.78E-05 | NCB2 BUR6 OPI1 SUB1 MET4 | 5 | 37 |
| regulation of phosphate metabolism [01.04.04] | 0.001056 | YPI1 PIG2 VHS3 | 3 | 18 |
| cell growth / morphogenesis [40.01] | 0.001386 | SCS22 ABP1 CLC1 ELM1 RHO5 SLA2 BNI1 EGT2 | 8 | 189 |
| biosynthesis of vitamins, cofactors, and prosthetic groups [01.07.01] | 0.001582 | RIB1 BNA1 BIO5 BIO4 BIO3 VHS3 | 6 | 110 |
| small GTPase mediated signal transduction [30.01.05.05.01] | 0.004298 | WSC4 RHO5 BNI1 ZEO1 | 4 | 58 |
| enzyme inhibitor [18.02.01.02] | 0.005542 | YPI1 VHS3 | 2 | 10 |
| G1/S transition of mitotic cell cycle [10.03.01.01.03] | 0.008598 | PTK2 SIC1 VHS3 | 3 | 37 |
| cytokinesis (cell division) /septum formation and hydrolysis [10.03.03] | 0.0088 | ELM1 BNI1 EGT2 DSE4 | 4 | 71 |
| budding, cell polarity and filament formation [43.01.03.05] | 0.009158 | NRG2 TEC1 ABP1 AFR1 SEC9 ELM1 CHS5 SLA2 BNI1 | 9 | 312 |
| extracellular polysaccharide degradation [01.25.01] | 0.01151 | CHS5 | 1 | 1 |
| metabolism of cyclic and unusual nucleotides [01.03.10] | 0.015901 | RIB1 PDE1 | 2 | 17 |
| regulation of glycolysis and gluconeogenesis [02.01.03] | 0.019699 | NRG2 UBC8 | 2 | 19 |
| actin cytoskeleton [42.04.03] | 0.024281 | ABP1 WSC4 SLA2 BNI1 | 4 | 96 |
| general transcription activities [11.02.03.01] | 0.025991 | TEC1 NCB2 BUR6 OPI1 MET4 | 5 | 146 |
| metabolism of energy reserves (e.g. glycogen, trehalose) [02.19] | 0.026269 | YPI1 PIG2 ATH1 | 3 | 56 |
| fermentation [02.16] | 0.030643 | AAD10 FDH1 | 2 | 24 |
| vacuolar/lysosomal transport [20.09.13] | 0.03099 | ATG8 CLC1 SNF7 APS1 PEP12 | 5 | 153 |
| bud / growth tip [42.29] | 0.04904 | ELM1 BNI1 | 2 | 31 |
| vesicle fusion [20.09.07.27] | 0.054876 | SEC9 PEP12 | 2 | 33 |
| degradation of lysine [01.01.06.06.02] | 0.056257 | LYS1 | 1 | 5 |
| C-1 compound catabolism [01.05.05.07] | 0.056257 | FDH1 | 1 | 5 |
| cell cycle [10.03] | 0.056257 | PCL9 | 1 | 5 |
| polysaccharide metabolism [01.05.03] | 0.060943 | CHS5 EGT2 | 2 | 35 |
| pheromone response, mating-type determination, sex-specific proteins [34.11.03.07] | 0.06593 | AFR1 CHS5 SLA2 BNI1 MF(ALPHA)1 | 5 | 189 |
| sugar, glucoside, polyol and carboxylate catabolism [01.05.02.07] | 0.066083 | GPM2 DSE4 ATH1 | 3 | 81 |
| receptor enzyme mediated signalling [30.05.01] | 0.067127 | WSC4 | 1 | 6 |
| eukaryotic plasma membrane [42.02] | 0.067127 | ZEO1 | 1 | 6 |
| antiporter [20.03.02.03] | 0.077874 | NHA1 | 1 | 7 |
| G-protein coupled receptor signalling pathway [30.05.02.24] | 0.077874 | AFR1 | 1 | 7 |
| assembly of protein complexes [14.10] | 0.078439 | ABP1 UBC8 APS1 TMA10 SLA2 | 5 | 199 |
| protein binding [16.01] | 0.079134 | ATG8 NRG2 ABP1 UBC8 SIC1 APS1 SLA2 BNI1 | 8 | 391 |
| regulation of nitrogen metabolism [01.02.07.01] | 0.088499 | DAL80 | 1 | 8 |
| metabolism of cysteine [01.01.09.03] | 0.099003 | MET4 | 1 | 9 |
| aminoadipic acid pathway [01.01.06.06.01.03] | 0.099003 | LYS1 | 1 | 9 |
| C-2 compound and organic acid catabolism [01.05.06.07] | 0.099003 | ADH2 | 1 | 9 |

‘k’ indicates the number of genes from input cluster in given category whereas ‘f’ indicates the total number of genes in given category.
