# Supplementary material for: A systematic assessment of chemical, genetic, and epigenetic factors influencing the activity of anticancer drug KP1019 (FFC14A)
Source: Oncotarget. 2017 Sep 30;8(58):98426–54. doi: 10.18632/oncotarget.21416 (PMC5716741; doi:10.18632/oncotarget.21416)
Supplement: Supplementary file 6 [file oncotarget-08-98426-s006.docx]

**Supplementary Table 5: Functional phenotypes associated with the KP1019 sensitive histone H3/H4 library mutants**

| **Mutant** | **Domain** | **Score**  **for KP1019** | **PTM** | **Ribosomal Silencing** | **Telomeric Silencing** | **Mating Efficiency** | **Growth Rate** | **DNA Damage** | | | | | **DNA Damage**  **Summary** | **Spt- phenotype** | **Transcription elongation defect** | **K56 Hyperacetylation Suppression** | **HMR Silencing** |
| --- | --- | --- | --- | --- | --- | --- | --- | --- | --- | --- | --- | --- | --- | --- | --- | --- | --- |
|  |  |  |  |  |  |  |  | **Benomyl** | **Camptothecin sensitivity** | **HU sensitivity** | **MMS sensitivity** | **UV irradiation** |  |  |  |  |  |
| H3-R2A | tail | -2 |  | -1 |  |  |  |  |  |  |  |  |  |  |  |  |  |
| H3-Q5A | tail | -3 |  |  |  |  |  |  |  |  |  |  |  |  |  |  |  |
| H3-R8A | tail | -3 |  | -2 |  |  |  |  |  |  |  |  |  |  |  |  |  |
| H3-K14A | tail | -2 | Ac,Me2,Me | -1 |  |  |  |  |  |  |  |  |  |  |  | -1 |  |
| H3-S28A | tail | -2 |  |  |  |  |  |  |  |  |  |  |  |  |  |  |  |
| H3-K42A | lateral | -4 |  | 1 |  |  | -0.29 |  |  | -2 | -2 |  | -0.57 |  |  |  | -1 |
| H3-G44A | lateral | -2 |  | 2 |  |  |  |  | -1 | -2 | -2 | -1 | -1.43 | 2 | -0.5 |  |  |
| H3-R49A | lateral | -3 |  | -2 | -2 |  | -0.29 | -1 |  | -2 | -2 | -1 | -1.43 | 2 | -0.5 |  |  |
| H3-K56A | lateral | -4 | Ac | -2 |  |  |  |  | -2 | -2 | -2 | -1 | -1.4 | 1 | -0.5 | 1 |  |
| H3-E59A | disk | -3 |  |  |  |  |  |  | -1 | -1 | -1 |  | -0.43 | 1 |  |  |  |
| H3-L60A | disk | -4 |  | -2 |  |  | -0.14 |  |  | -1 | -1 |  | -0.29 |  |  |  |  |
| H3-R63A | lateral | -4 |  |  |  |  |  |  |  |  |  |  |  |  |  |  |  |
| H3-Q68A | disk | -3 |  |  |  |  |  |  |  |  |  |  |  | 2 |  |  |  |
| H3-T80A | disk | -3 |  |  |  |  | -0.29 |  |  |  |  | -1 | -0.14 |  |  |  | -2 |
| H3-I89A | disk | -3 |  |  | -2 |  | -0.33 |  |  |  |  |  |  |  |  |  | -1 |
| H3-A98S | disk | -2 |  |  |  |  |  |  |  |  |  |  |  |  |  |  |  |
| H3-Y99A | buried | -3 |  |  |  |  | -0.33 |  |  |  |  |  |  |  |  |  |  |
| H3-L100A | buried | -2 |  |  |  |  |  |  |  |  |  |  |  |  |  |  | -1 |
| H3-F104A | buried | -4 |  | -1 | -1 |  | -0.67 |  | -1 | -1 |  |  | -0.4 |  | -1 |  | -1 |
| H3-D106A | disk | -2 |  | 1 |  |  | -0.17 |  |  |  |  |  |  |  |  |  |  |
| H3-A110S | buried | -2 |  |  | -1 |  | -0.2 |  |  |  |  |  |  |  |  |  | -1 |
| H3-A114S | disk | -3 |  |  | -1 |  | -0.2 |  |  |  |  |  |  |  |  |  |  |
| H3-V117A | lateral | -3 |  |  | -1 |  | -0.14 |  |  |  |  |  |  | 2 |  |  |  |
| H3-Q120A | lateral | -3 |  |  | -1 |  |  |  |  |  |  |  |  | 2 |  |  | -1 |
| H3-E133A | disk | -2 |  |  |  |  |  |  |  |  |  |  |  |  |  |  |  |
| H3-R134A | disk | -2 |  |  |  |  |  |  |  |  |  |  |  |  |  |  |  |
| H3-S135A | tail | -2 |  |  |  |  |  |  |  |  |  |  |  |  |  |  |  |
| H3-K18R | tail | -3 | Ac,Me |  |  |  |  |  |  |  |  |  |  |  |  |  |  |
| H3-K27R | tail | -2 | Ac,Me3,Me2,Me |  | 1 |  |  |  |  |  |  |  |  |  |  |  |  |
| H3-K56R | lateral | -4 | Ac | -1.5 | 0.5 |  |  |  | -2 | -2 |  | -1 | -1.12 |  |  | 1.67 |  |
| H3-K115R | lateral | -3 |  |  |  |  |  |  |  |  |  |  |  |  |  |  |  |
| H3-K122R | disk | -3 |  |  | -1.5 |  | -0.2 |  |  |  |  |  |  |  |  | -0.33 | -1 |
| H3-K18Q | tail | -4 | Ac,Me | -1 |  |  |  |  |  |  |  |  |  |  |  |  |  |
| H3-K36Q | tail | -3 | Ac,Me3,Me2,Me | 1 |  |  |  |  |  |  |  |  |  |  |  | 0.67 | -1 |
| H3-K37Q | tail | -2 |  | 1 | -1 |  |  |  |  |  |  |  |  |  |  |  |  |
| H3-K56Q | lateral | -4 | Ac |  | -0.5 |  |  |  | -1 | -1 |  |  | -0.25 |  |  | 2 | -1 |
| H3-K122Q | disk | -3 |  |  | -1 |  |  |  |  |  |  |  |  |  |  | -2 |  |
| H3-R69K | lateral | -4 |  |  |  |  |  |  |  |  |  |  |  |  |  |  |  |
| H3-D81N | disk | -2 |  |  | 1 |  |  |  | -1 |  |  |  | -0.2 |  |  |  | -1 |
| H3-E105Q | disk | -3 |  | 2 |  |  | -0.4 |  |  |  |  |  |  |  |  |  |  |
| H3-T58D | disk | -4 |  | -2 | -2 |  | -0.4 |  | -1 | -2 |  |  | -0.6 |  |  |  |  |
| H3-S86D | lateral | -2 |  |  |  |  | -0.4 |  |  | -1 |  |  | -0.2 |  |  |  |  |
| H3-S135D | tail | -2 |  |  |  |  |  |  |  |  |  |  |  |  |  |  |  |
| H3-Y99E | buried | -3 |  |  | -1 |  | -0.6 |  |  |  | -1 |  | -0.2 |  |  |  | -1 |
| H3-P30V | tail | -3 |  |  |  |  |  |  |  |  |  |  |  |  |  |  |  |
| H3-K9,14,18,23A |  | -4 |  | -2 |  |  |  |  |  |  |  |  |  |  |  |  |  |
| H3-∆1-4 |  | -4 |  |  |  |  | -0.2 |  |  |  |  |  |  |  |  |  |  |
| H3-∆1-24 |  | -4 |  | -2 | -1 |  |  |  |  |  | -1 |  | -0.2 |  | -1 |  |  |
| H3-∆1-28 |  | -4 |  | -2 | -1 |  | -0.4 |  |  | -1 | -2 |  | -0.6 |  | -1 |  |  |
| H3-∆5-8 |  | -3 |  | -1 |  |  |  |  |  |  | -2 |  | -0.4 |  |  |  |  |
| H3-∆5-16 |  | -4 |  | -1 |  |  |  |  |  |  | -1 |  | -0.2 |  |  |  |  |
| H3-∆5-24 |  | -4 |  | -2 |  |  |  |  |  |  | -1 |  | -0.2 |  | -1 |  |  |
| H3-∆5-32 |  | -4 |  | -2 |  |  | -0.2 |  |  | -1 |  |  | -0.2 |  | -1 |  |  |
| H3-∆9-16 |  | -4 |  | -2 |  |  |  |  |  |  |  |  |  |  |  |  |  |
| H3-∆9-20 |  | -4 |  | -1 |  |  |  |  |  |  | -1 |  | -0.2 |  |  |  |  |
| H3-∆9-24 |  | -4 |  | -2 |  |  |  |  |  |  | -1 |  | -0.2 |  | -1 |  |  |
| H3-∆13-28 |  | -4 |  | -1 |  |  |  |  |  |  | -1 |  | -0.2 |  |  |  |  |
| H3-∆17-28 |  | -3 |  | -2 |  |  | -0.2 |  |  |  |  |  |  |  | -1 |  |  |
| H3-∆21-36 |  | -3 |  | -2 | -1 |  | -0.4 |  |  |  |  |  |  |  | -1 |  |  |
| H3-∆29-36 |  | -4 |  |  |  |  |  |  |  |  |  | -1 | -0.2 |  |  |  |  |
| H3-∆1-20 |  | -4 |  | -2 | -2 |  | -0.6 |  |  | -1 | -1 |  | -0.4 |  | -1 |  | -1 |
| H3-∆1-28 |  | -4 |  | -2 | -1 |  | -0.4 |  |  | -1 | -2 |  | -0.6 |  | -1 |  |  |
| H3-∆4-30 |  | -4 |  | -2 | -1 |  |  |  |  | -1 | -2 |  | -0.6 |  | -1 |  | -1 |
| H3-∆4-35 |  | -4 |  |  | -2 |  | -0.2 |  | -1 | -2 | -2 | -1 | -1.2 |  |  |  |  |
| H4-K16A | tail | -3 | Ac |  | -2 | -2 |  |  |  |  |  |  |  |  |  | 1 | -2 |
| H4-K44A | lateral | -3 |  | 1 |  |  |  |  |  |  |  |  |  |  |  |  | -1 |
| H4-A56S | disk | -3 |  |  |  |  |  |  |  |  |  |  |  |  |  |  |  |
| H4-S64A | disk | -2 | Ph |  |  |  |  |  |  |  |  |  |  |  |  |  |  |
| H4-V81A | buried | -2 |  | -1.5 | -1.5 |  | -0.17 |  | -1 |  |  |  | -0.2 |  |  | -0.67 | -1 |
| H4-D85A | buried | -3 |  | -1 | -1 |  | -0.33 |  |  |  |  |  |  |  |  |  |  |
| H4-Y88A | disk | -4 |  |  |  |  |  |  |  |  |  |  |  |  |  |  |  |
| H4-L97A | buried | -2 |  |  |  |  |  |  |  | -2 | -2 |  | -0.57 |  | -0.5 |  |  |
| H4-K8Q | tail | -2 | Ac | -1 |  |  |  |  |  |  |  |  |  |  |  | -0.67 |  |
| H4-K12Q | tail | -2 | Ac |  |  |  |  |  |  |  |  |  |  |  |  |  |  |
| H4-R3K | tail | -3 |  |  |  |  |  |  |  |  |  |  |  |  |  |  | -1 |
| H4-R36K | lateral | -3 |  | -1 |  |  | -0.4 |  |  |  | -2 |  | -0.4 |  |  |  |  |
| H4-R78K | lateral | -2 |  |  | -1 |  | -0.2 |  |  | -1 |  |  | -0.2 |  |  |  |  |
| H4-T96D | disk | -3 |  |  | 1 |  |  |  |  |  |  |  |  |  |  |  |  |
| H4-Y88E | disk | -3 |  |  |  |  | -0.4 |  |  |  |  |  |  |  |  |  |  |
| H4-Y51F | disk | -2 |  |  |  |  |  |  |  |  |  |  |  |  |  |  |  |
| H4-∆1-4 |  | -3 |  | -2 |  |  |  |  |  |  | -1 |  | -0.2 |  |  |  | -2 |
| H4-∆5-8 |  | -2 |  | -1 |  |  |  |  |  |  |  |  |  |  |  |  |  |
| H4-∆5-12 |  | -2 |  |  | 1 |  |  |  |  |  |  |  |  |  |  |  |  |

The domain, PTM, and phenotype information along with their respective scores (except for KP1019) for each of H3/H4 mutants was extracted from the HistoneHits database, which can be accessed at <http://54.235.254.95/histonehits/>. The scores for KP1019 sensitive mutants were given as described in the ‘Materials and Methods’ section.
