# Supplementary material for: A systematic assessment of chemical, genetic, and epigenetic factors influencing the activity of anticancer drug KP1019 (FFC14A)
Source: Oncotarget. 2017 Sep 30;8(58):98426–54. doi: 10.18632/oncotarget.21416 (PMC5716741; doi:10.18632/oncotarget.21416)
Supplement: Supplementary file 7 [file oncotarget-08-98426-s007.docx]

**Supplementary Table 6: Functional phenotypes associated with the KP1019 resistant histone H3/H4 library mutants**

| **Mutant** | **Domain** | **Score**  **for KP1019** | **PTM** | **Ribosomal Silencing** | **Telomeric Silencing** | **Mating Efficiency** | **Growth Rate** | **DNA Damage agents** | | | | | **DNA Damage**  **Summary** | **Spt- phenotype** | **Transcription elongation defect** | **K56 Hyperacetylation Suppression** | **HMR Silencing** |
| --- | --- | --- | --- | --- | --- | --- | --- | --- | --- | --- | --- | --- | --- | --- | --- | --- | --- |
|  |  |  |  |  |  |  |  | **Benomyl** | **Camptothecin sensitivity** | **HU sensitivity** | **MMS sensitivity** | **UV irradiation** |  |  |  |  |  |
| H3-K4A | tail | 2 | Me3,Me2,Me | -1 |  |  |  |  |  |  |  |  |  |  |  |  |  |
| H3-K23A | tail | 4 | Ac,Me |  |  |  |  |  |  |  |  |  |  |  |  |  |  |
| H3-G34A | tail | 4 |  |  |  |  |  |  |  |  |  |  |  |  |  |  |  |
| H3-R69A | lateral | 2 |  | -1 | -2 |  |  |  |  |  |  |  |  | 2 |  |  |  |
| H3-L70A | buried | 4 |  |  | -2 |  |  |  |  |  |  |  |  |  |  |  | -1 |
| H3-E73A | disk | 4 |  | 2 | -2 | -1 |  |  |  |  |  |  |  |  |  |  | -2 |
| H3-A75S | buried | 4 |  |  |  |  |  |  |  |  |  |  |  |  |  |  |  |
| H3-R83A | lateral | 3 |  |  | -1 |  |  |  |  |  | -1 |  | -0.14 |  |  |  |  |
| H3-A88S | buried | 4 |  |  |  |  | -0.2 |  |  |  |  |  |  |  |  |  |  |
| H3-G90A | disk | 4 |  | -1 |  |  |  |  |  |  |  |  |  |  |  |  |  |
| H3-E94A | disk | 2 |  |  | 1 |  |  |  |  | -1 | -1 |  | -0.29 |  |  |  |  |
| H3-V96A | buried | 4 |  |  |  |  |  |  |  |  |  |  |  |  |  |  |  |
| H3-K122A | disk | 4 |  |  | -1 |  | -0.14 |  |  |  | -1 | -1 | -0.2 |  |  | -2 |  |
| H3-K4R | tail | 4 | Me3,Me2,Me | -2 | -1 |  | -0.2 |  |  |  |  |  |  |  |  |  |  |
| H3-R2K | tail | 3 |  |  | 1 |  |  |  |  |  |  |  |  |  |  | -0.33 |  |
| H3-Q76E | disk | 3 |  |  | -2 |  |  |  |  |  |  |  |  |  |  |  |  |
| H3-T3D | tail | 3 |  |  |  |  |  |  |  |  |  |  |  |  |  |  |  |
| H3-H39Q | lateral | 3 |  |  |  |  |  |  |  |  |  |  |  |  |  |  | -1 |
| H3-∆13-36 |  | 3 |  | -2 | -1 |  |  |  |  | -1 | -1 |  | -0.4 |  | -1 |  |  |
| H3-∆17-32 |  | 3 |  | -2 | -1 |  |  |  |  |  |  |  |  |  | -1 |  |  |
| H3-∆21-24 |  | 2 |  |  |  |  |  |  |  |  |  |  |  |  |  |  |  |
| H3-∆4-20 |  | 4 |  | -1 |  |  |  |  |  |  |  |  |  |  |  |  |  |
| H4-G4A | tail | 2 |  |  |  |  |  |  |  |  |  |  |  |  |  |  |  |
| H4-K5A | tail | 2 | Ac | -1 | -2 | -2 |  |  |  |  |  |  |  |  |  |  |  |
| H4-G6A | tail | 4 |  |  |  |  |  |  |  |  |  |  |  |  |  |  |  |
| H4-K8A | tail | 2 | Ac | -1 |  |  |  |  |  |  |  |  |  |  |  | -0.67 |  |
| H4-L10A | tail | 4 |  |  |  |  |  |  |  |  |  |  |  |  |  |  |  |
| H4-G13A | tail | 4 |  | -2 | 1 |  |  |  |  |  |  |  |  |  |  |  |  |
| H4-R17A | tail | 3 |  |  | -2 |  |  |  |  |  |  |  |  |  |  |  |  |
| H4-H18A | disk | 4 |  | -1 | -2 | -2 |  |  |  |  |  |  |  |  |  |  | -2 |
| H4-R19A | disk | 2 |  |  | -2 |  |  |  |  |  |  |  |  |  |  |  | -2 |
| H4-K20A | disk | 2 | Me |  | -1 |  |  |  |  |  |  |  |  |  |  | 1 |  |
| H4-R23A | lateral | 3 |  | -1 | -2 |  |  |  |  |  |  |  |  |  |  |  | -2 |
| H4-I26A | disk | 4 |  |  | -2 |  |  |  |  |  |  |  |  |  |  |  | -1 |
| H4-I29A | buried | 3 |  |  |  |  | -0.29 |  |  |  |  |  |  |  |  |  | -2 |
| H4-G48A | lateral | 2 |  |  |  |  |  |  |  |  |  |  |  | 1 |  |  |  |
| H4-R55A | disk | 2 |  | 1 | -2 |  |  |  |  |  |  |  |  |  |  |  | -1 |
| H4-V57A | buried | 2 |  | -1 |  |  |  |  |  |  |  |  |  |  |  |  |  |
| H4-S60A | disk | 2 |  |  |  |  |  |  |  |  |  |  |  |  |  |  | -1 |
| H4-V65A | buried | 3 |  |  |  |  |  |  |  |  |  |  |  |  |  |  | -1 |
| H4-T71A | disk | 2 |  |  |  |  |  |  |  |  |  |  |  |  |  |  |  |
| H4-K79A | lateral | 2 | Me | -2 | -2 |  |  |  |  |  |  |  |  |  |  | 0.33 |  |
| H4-V87A | buried | 3 |  |  |  |  |  |  |  |  |  |  |  |  |  |  |  |
| H4-K91A | disk | 4 | Ac |  | -0.5 |  | -0.5 |  |  | -1 | -1 |  | -0.25 |  |  | -0.33 | -2 |
| H4-Q93A | disk | 2 |  | -1 |  |  |  |  |  |  |  |  |  |  |  |  |  |
| H4-Y98A | buried | 4 |  |  |  |  | -0.29 |  |  | -2 | -2 |  | -0.57 |  |  |  | -2 |
| H4-F100A | disk | 3 |  |  | 1 |  |  |  |  | -1 | -1 |  | -0.29 |  |  |  |  |
| H4-G101A | disk | 4 |  |  |  |  |  |  |  |  |  |  |  |  |  |  |  |
| H4-G102A | disk | 4 |  | -1 |  |  |  |  |  |  |  |  |  |  |  |  |  |
| H4-K5R | tail | 2 | Ac |  |  |  |  |  |  |  |  |  |  |  |  |  |  |
| H4-K16R | tail | 2 | Ac |  |  |  |  |  |  |  |  |  |  |  |  | 1.33 | -2 |
| H4-K20R | disk | 4 | Me |  |  |  |  |  |  |  |  |  |  |  |  |  |  |
| H4-K44R | lateral | 3 |  |  |  |  | -0.2 |  |  | -1 |  |  | -0.2 |  | -1 |  |  |
| H4-K59R | disk | 4 | Me |  | -2 |  |  |  |  |  |  |  |  |  |  | -0.33 |  |
| H4-K79R | lateral | 2 | Me | -1 |  |  |  |  |  |  |  |  |  |  |  | 0.33 |  |
| H4-K5,8,12,16R |  | 3 |  |  |  |  | -0.75 | -1 |  | -1 |  |  | -0.4 |  |  |  |  |
| H4-K5Q | tail | 2 | Ac | -1 |  |  |  |  |  |  |  |  |  |  |  | -0.67 |  |
| H4-K16Q | tail | 4 | Ac |  | -2 | -2 |  |  |  |  |  |  |  |  |  | 0.67 | -2 |
| H4-H18Q | disk | 4 |  |  | -2 |  |  |  |  |  |  |  |  |  |  |  | -2 |
| H4-K20Q | disk | 2 | Me | -1 | -2 |  |  |  |  |  |  |  |  |  |  | 0.67 | -1 |
| H4-K59Q | disk | 2 | Me | -2 | -2 |  |  |  |  | -1 |  |  | -0.12 |  |  | -0.33 | -1 |
| H4-H75Q | buried | 2 |  | -1 | -2 |  |  |  |  |  |  |  |  |  |  |  |  |
| H4-K5,8,12,16Q |  | 4 |  |  | -2 | -2 | -0.4 |  | -1 | -1 | -2 |  | -0.8 |  |  |  | -2 |
| H4-N25D | disk | 4 |  | 1 | -2 | -1 |  |  |  |  |  |  |  |  |  |  | -2 |
| H4-T30D | lateral | 4 |  |  | -2 |  |  |  |  |  |  |  |  |  |  |  | -1 |
| H4-S47D | lateral | 4 | Ph |  |  |  |  |  |  |  |  |  |  |  |  |  |  |
| H4-R19K | disk | 4 |  |  |  |  |  |  |  |  |  |  |  |  |  |  |  |
| H4-R23K | lateral | 4 |  |  | -2 |  |  |  |  |  |  |  |  |  |  |  | -1 |
| H4-D68N | disk | 3 |  | -1 |  |  |  |  |  |  |  |  |  |  |  |  |  |
| H4-∆1-8 |  | 4 |  | -2 | -1 |  |  |  |  |  |  |  |  |  |  |  |  |
| H4-∆1-12 |  | 4 |  |  | -1 |  |  |  |  | -1 |  |  | -0.2 |  |  |  | -1 |
| H4-∆1-24 |  | 4 |  |  |  |  | -0.8 |  |  |  |  |  |  |  |  |  |  |
| H4-∆9-12 |  | 4 |  |  | 1 |  |  |  |  |  |  |  |  |  |  |  |  |
| H4-∆9-16 |  | 3 |  |  |  |  |  |  |  |  |  |  |  |  |  |  | -1 |
| H4-∆9-20 |  | 4 |  |  | -2 | -2 |  |  |  |  |  |  |  |  | -1 |  | -2 |
| H4-∆13-16 |  | 3 |  |  | 2 | -2 |  |  |  |  |  |  |  |  |  |  |  |
| H4-∆13-20 |  | 4 |  |  | -2 | -2 |  |  |  |  |  |  |  |  |  |  | -2 |
| H4-∆13-24 |  | 4 |  | -1 | -2 |  |  |  |  | -1 |  |  | -0.2 |  | -1 |  | -2 |
| H4-∆17-20 |  | 4 |  |  | -2 | -2 |  |  |  |  |  |  |  |  |  |  | -2 |
| H4-∆17-24 |  | 2 |  |  | -2 | -2 |  |  |  | -1 |  |  | -0.2 |  |  |  | -2 |
| H4-∆20-23 |  | 4 |  | 1 | -2 | -2 |  |  |  |  |  |  |  |  |  |  | -2 |
| H4-∆21-24 |  | 3 |  | 1 | -2 | -2 |  |  |  |  |  |  |  |  |  |  | -2 |

The domain, PTM, and phenotype information along with their respective scores (except for KP1019) for each of H3/H4 mutants was extracted from the HistoneHits database, which can be accessed at <http://54.235.254.95/histonehits/>. The scores for KP1019 resistant mutants were given as described in the ‘Materials and Methods’ section.
